# Neoadjuvant camrelizumab (an anti-PD-1 antibody) plus chemotherapy or apatinib (a VEGFR-2 inhibitor) for initially unresectable stage II–III non-small-cell lung cancer: a multicentre, two-arm, phase 2 exploratory study

**DOI:** 10.1038/s41392-024-01861-w

**Published:** 2024-06-14

**Authors:** Haoran Xia, Han Zhang, Zheng Ruan, Huibiao Zhang, Liangdong Sun, Hezhong Chen, Yongxin Zhou, Lele Zhang, Dongliang Bian, Xinsheng Zhu, Jing Zhang, Fenghuan Sun, Huansha Yu, Nan Song, Xiaogang Liu, Yuming Zhu, Haiping Zhang, Wenxin He, Jian Chen, Jie Yang, Guohan Chen, Shiliang Xie, Dongfang Tang, Xiaomiao Zhang, Liang Duan, Deping Zhao, Qinchuan Li, Peng Zhang, Gening Jiang

**Affiliations:** 1 Department of Thoracic Surgery, Shanghai Pulmonary Hospital, Tongji University School of Medicine, Shanghai, China; 2 Department of Thoracic Surgery, Shanghai General Hospital, Shanghai Jiao Tong University School of Medicine, Shanghai, China; 3 Department of Thoracic Surgery, Huadong Hospital Affiliated to Fudan University, Shanghai, China; 4 Department of Thoracic Surgery, Changhai Hospital, Naval Medical University, Shanghai, China; 5 Department of Thoracic-Cardiovascular Surgery, Tongji Hospital, Tongji University School of Medicine, Shanghai, China; 6 Central Laboratory, Shanghai Pulmonary Hospital, Tongji University School of Medicine, Shanghai, China; 7 Experimental Animal Center, Shanghai Pulmonary Hospital, Tongji University School of Medicine, Shanghai, China; 8 Department of Oncology, Shanghai Pulmonary Hospital, Tongji University School of Medicine, Shanghai, China; 9 Department of Thoracic Surgery, Shanghai East Hospital, Tongji University School of Medicine, Shanghai, China

**Keywords:** Lung cancer, Clinical trials

## Abstract

This multicentre, two-arm, phase 2 study aimed to explore the efficacy and safety of neoadjuvant camrelizumab plus chemotherapy or apatinib in patients with initially unresectable stage II–III non-small-cell lung cancer (NSCLC). Eligible patients regardless of PD-L1 expression received neoadjuvant camrelizumab 200 mg and platinum-doublet chemotherapy every 3 weeks (arm A) or those with PD-L1-positive tumors received neoadjuvant camrelizumab and apatinib 250 mg once daily (arm B), for 2–4 cycles, followed by surgery. The primary endpoint was major pathological response (MPR) rate. Thirty patients in arm A and 21 in arm B were enrolled. Surgery rates were 50.0% (15/30) in arm A and 42.9% (9/21) in arm B, with all patients achieving R0 resections. Of these patients, the MPR and pathological complete response rates were both 20.0% (95% CI 4.3–48.1) in arm A and were 55.6% (95% CI 21.2–86.3) and 11.1% (95% CI 0.3–48.2) in arm B, respectively. The corresponding objective response rates were 33.3% (95% CI 11.8–61.6) and 55.6% (95% CI 21.2–86.3). With a median follow-up of 22.4 months (95% CI 19.0–26.0), the median event-free survival was not reached (NR; 95% CI 13.6-NR) in arm A and 16.8 months (95% CI 8.6-NR) in arm B. Grade 3 or above treatment-related adverse events occurred in eight (26.7%) patients in arm A and three (14.3%) in arm B. Biomarker analysis showed baseline TYROBP expression was predictive of treatment response in arm B. Neoadjuvant camrelizumab plus chemotherapy or apatinib exhibits preliminary efficacy and manageable toxicity in patients with initially unresectable stage II–III NSCLC.

## Introduction

Lung cancer is the second most common malignancy and the leading cause of cancer-related death, with approximately 2.2 million new incidences and 1.8 million deaths worldwide in 2020.^1^ In China, it is estimated that lung cancer continues to be the most prevalent malignant disease with the highest incidence (1.06 million cases) and mortality (0.73 million deaths) in 2022 according to the latest statistics from the National Cancer Center of China.^2^ Non-small-cell lung cancer (NSCLC), which is the predominant pathological subtype of lung cancer, constitutes 85 to 90% of all cases.^3^ Nearly half of patients are initially diagnosed with early-stage, localized or regional NSCLC, which can be further classified as resectable, potentially resectable or initially unresectable disease according to the status of the tumor and lymph nodes.^4,5^ For these three disease classifications, treatment options vary.

Over the last decade, immunotherapy with anti-programmed cell death-(ligand) 1 (PD-[L]1) antibodies have made breakthroughs and changed the comprehensive therapeutic paradigm for advanced driver-negative NSCLC. Given the outstanding efficacy of immunotherapy in advanced disease and the association between high tumor burden and limited antigenic heterogeneity with improved efficacy of anti-PD-(L)1 antibodies, which indicates an optimal timing for immunotherapy possibly in the neoadjuvant setting,^6^ immunotherapy-based neoadjuvant strategies have been investigated in resectable or potentially resectable NSCLC. Three randomized phase 3 trials evaluated the anti-PD-1 antibodies nivolumab (CheckMate 816),^7^ pembrolizumab (KEYNOTE-671)^8^ and toripalimab (Neotorch)^9^ and a fourth trial evaluated the anti-PD-L1 antibody durvalumab (AEGEAN)^10^ in combination with chemotherapy as neoadjuvant therapy for resectable or potentially resectable NSCLC, and showed substantial improvements in both event-free survival (EFS) and pathological complete response (pCR) when compared with neoadjuvant chemotherapy alone. Currently, this neoadjuvant strategy of immunotherapy combined with chemotherapy has been recommended for resectable or potentially resectable tumors without driver-gene alterations. In addition to this strategy, immunotherapy combined with antiangiogenic therapy are being explored in this population, under the premise of the remodeling of immunosuppressive tumor microenvironment and enhancement antitumor immunity with antiangiogenic agents, reflecting synergistic antitumor effects with immunotherapy.^11,12^ The EAST ENERGY study evaluated neoadjuvant pembrolizumab plus ramucirumab (an anti-vascular endothelial growth factor receptor-2 [VEGFR-2] monoclonal antibody) for patients with resectable PD-L1-positive stage IB-IIIA NSCLC and showed encouraging pathological results, with a major pathological response (MPR) rate of 50.0% and a pCR rate of 25.0%.^13^ Of note, although these trials stated the inclusion of “resectable tumors”, the definition of resectability was ambiguous. In several studies, such as NADIM II and AEGEAN, patients with multiple-station N2 diseases were also enrolled,^10,14^ whereas this subpopulation is traditionally considered unresectable.

In 2017, PACIFIC study ushered in a new era for consolidation immunotherapy following definitive concurrent chemoradiotherapy in patients with initially unresectable NSCLC, which showed that durvalumab after chemoradiotherapy resulted in a significant improvement in progression-free survival (PFS), reducing the risk of progression or death by 48%.^15^ In light of the impressive data, it also established consolidation immunotherapy after concurrent chemoradiotherapy as the standard of care in initially unresectable stage II/III NSCLC. However, the optimal modality for this patient subsets has been controversial, particularly with regard to the issue of whether unresectable stage II/III NSCLC could be converted to resectable disease after downstaging with neoadjuvant immunotherapy. To date, only a few data from retrospective studies showed that neoadjuvant immunotherapy followed by surgery might be safe and feasible in initially unresectable NSCLC.^16–18^ For example, Deng and his colleagues evaluated 51 patients with initially unresectable stage IIIB NSCLC who received anti-PD-1 antibodies combined with platinum-based chemotherapy, and found that patients who underwent surgery after chemoimmunotherapy had a median disease-free survival (DFS) or PFS of 27.5 months, which was relatively longer than those who did not (4.7 months for non-responders and 16.7 months for responders).^18^ However, prospective evidence remains scarce in this setting.

Camrelizumab, a humanized high-affinity IgG4-kappa anti-PD-1 monoclonal antibody, has been approved as a first-line treatment in combination with chemotherapy for advanced NSCLC in China,^19,20^ and has also been reported to exert promising pathological outcomes when combined with chemotherapy or antiangiogenic agent apatinib (a tyrosine kinase inhibitor targeting VEGFR-2) as neoadjuvant therapy for resectable NSCLC.^21,22^ The TD-FOREKNOW trial revealed that the addition of camrelizumab to neoadjuvant platinum-based chemotherapy significantly improved the pCR rate in patients with resectable stage III NSCLC compared with neoadjuvant chemotherapy alone (32.6% vs 8.9%; odds ratio 4.95; 95% confidence interval [CI] 1.35–22.37).^21^ Meanwhile, a phase 2 study conducted by Zhao et al. reported that neoadjuvant camrelizumab combined with apatinib yielded an MPR rate of 57% and a pCR rate of 23% in patients with resectable stage IIA-IIIB (T3N2 only) NSCLC.^22^ In this context, whether patients with unresectable NSCLC could also benefit from camrelizumab-based neoadjuvant therapy deserves to be explored. Herein, we report the results from a multicentre, two-arm, phase 2 study assessing the efficacy and safety of camrelizumab plus platinum-doublet chemotherapy (arm A) or plus apatinib (arm B) as neoadjuvant therapy in patients with initially unresectable stage II–III NSCLC.

## Results

### Patients and treatment

**Fig. 1:**
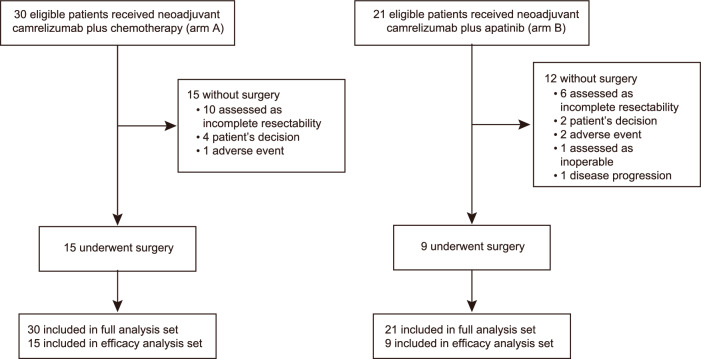
Patient flowchart

Between September 29, 2020, and June 29, 2022, 30 eligible Chinese patients in arm A and 21 in arm B were enrolled (Fig. 1). All these patients were treated with the pre-specified neoadjuvant regimen and included in the full analysis population. Baseline characteristics of the patients are shown in Table 1. The majority of patients were male, with 93.3% (28/30) in arm A and 81.0% (17/21) in arm B. In arm A, 23 (76.7%) patients had an Eastern Cooperative Oncology Group performance status (ECOG PS) of 0, while half of the patients (52.4%) had an ECOG PS of 1 in arm B. Additionally, 26 (86.7%) patients had stage IIIA disease and 14 (46.7%) had N2 disease in arm A; the numbers were 18 (85.7%) and 12 (57.1%) in arm B.

**Table 1:** Baseline characteristics

Characteristics	Arm A (*n* = 30)	Arm B (*n* = 21)
Age (years), median (range)	65 (19–74)	67 (53–73)
Sex, *n* (%)		
Male	28 (93.3)	17 (81.0)
Female	2 (6.7)	4 (19.0)
ECOG performance status, *n* (%)		
0	23 (76.7)	10 (47.6)
1	7 (23.3)	11 (52.4)
Smoking, *n* (%)		
Non-smoker	5 (16.7)	6 (28.6)
Former/current smoker	25 (83.3)	15 (71.4)
Clinical stage, *n* (%)		
IIB	1 (3.3)	1 (4.8)
IIIA	26 (86.7)	18 (85.7)
IIIB	3 (10.0)	2 (9.5)
Clinical nodal status, *n* (%)		
N0	9 (30.0)	4 (19.0)
N1	7 (23.3)	5 (23.8)
N2	14 (46.7)	12 (57.1)
Histological type, *n* (%)		
Squamous cell carcinoma	20 (66.7)	16 (76.2)
Non-squamous cell carcinoma	10 (33.3)	5 (23.8)
PD-L1 expression, *n* (%)		
<1%	12 (40.0)	0 (0)
1%–49%	7 (23.3)	12 (57.1)
≥50%	0 (0)	9 (42.9)
Unknown	11 (36.7)	0 (0)

Four (13.3%) of the 30 patients received only one cycle of neoadjuvant treatment in arm A, including two due to the COVID-19 pandemic, and one each due to patient’s decision and adverse events; while in arm B, all (100%) patients completed the neoadjuvant treatment cycles as planned. The median number of neoadjuvant treatment cycles was 3 in both arms. The median interval between the last dose of neoadjuvant therapy and surgery was 47 days (range 32–113) in arm A and 42 days (range 30–139) in arm B, respectively. Adjuvant regimens were available in 10 and seven patients in arms A and B, respectively. In arm A, eight (80.0%) patients received adjuvant platinum-doublet chemotherapy and one each (10.0%) received subsequent herbal medicine and camrelizumab monotherapy; while in arm B, four (57.1%) patients received adjuvant platinum-doublet chemotherapy, two (28.6%) received adjuvant chemoimmunotherapy and one (14.3%) received sintilimab monotherapy.

### Surgery

In arm A, 15 (50.0%) of the 30 patients underwent surgery, and the R0 resection rate was 100.0 (95% CI 78.2–100.0; Supplementary Table 1). In arm B, nine (42.9%) of the 21 patients underwent surgical resection, all of which (95% CI 66.4–100.0) had R0 resections. All these patients who underwent surgery (15 in arm A and nine in arm B) were included in the efficacy analysis population. The most common reason for failure to perform surgery was incomplete resectability in both arms (66.7% [10/15] in arm A and 50.0% [6/12] in arm B). The detailed reasons for each patient’s potential unresectable are shown in Supplementary information. Delays in surgery occurred in three (20.0%) patients in arm A and two (22.2%) patients in arm B. No neoadjuvant treatment-related surgical delays occurred.

In both arms, the use of video-assisted thoracoscopic surgery was more common (60.0% in arm A and 77.8% in arm B, respectively). The most frequent surgical procedure in both arms was lobectomy (46.7% and 44.4%, respectively; Supplementary Table 1 and Supplementary Fig. 1). Surgical complications were reported in three (20.0%) patients in arm A, two with postoperative infections and one with pulmonary embolism; while none in arm B. No patients died within 90 days after surgery in this study.

### Efficacy

In arm A, three (20.0%, 95% CI 4.3–48.1) of the 15 patients who underwent surgery had an MPR, all of which had a pCR (Table 2 and Supplementary Fig. 2). According to Response Evaluation Criteria in Solid Tumors (RECIST) version 1.1, five (33.3%) patients had a partial response (PR) and eight (53.3%) patients had stable disease (SD). The radiographic objective response rate (ORR) was 33.3% (95% CI 11.8–61.6). In arm B of nine patients who proceeded to surgery, five (55.6%, 95% CI 21.2–86.3) patients had an MPR, of whom one (11.1%, 95% CI 0.3–48.2) had a pCR (Table 2). According to the preoperative radiographic response evaluation, a PR was achieved in five (55.6%) patients, with the corresponding ORR of 55.6% (95% CI 21.2–86.3). SD was observed in four (44.4%) patients.

**Table 2:** Response rates in patients who underwent surgery

Efficacy outcomes	Arm A (*n* = 15)	Arm B (*n* = 9)
Pathological response, *n* (%, 95% CI)		
Major pathological response	3 (20.0, 4.3–48.1)	5 (55.6, 21.2–86.3)
Pathological complete response	3 (20.0, 4.3–48.1)	1 (11.1, 0.3–48.2)
Best overall response, *n* (%)		
Complete response	0	0
Partial response	5 (33.3)	5 (55.6)
Stable disease	8 (53.3)	4 (44.4)
Not evaluable	2 (13.3)	0
Objective response rate, % (95% CI)	33.3 (11.8–61.6)	55.6 (21.2–86.3)

For intention to treat patients regardless of surgery, the MPR and pCR rates were both 10% (3 of 30, 95% CI 2.1–26.5) and the ORR was 26.7% (8 of 30, 95% CI 12.3–45.9) in arm A; and in arm B, the MPR rate, pCR rate, and ORR were 23.8% (5 of 21, 95% CI 8.2–47.2), 4.8% (1 of 21, 95% CI 0.1–23.8) and 33.3% (7 of 21, 95% CI 14.6–57.0), respectively (Supplementary Table 2).

**Fig. 2:**
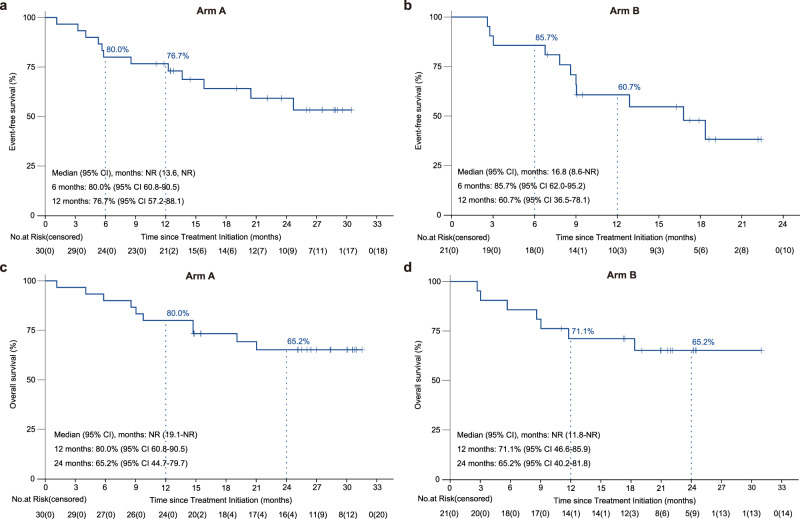
Kaplan–Meier curves for event-free survival (EFS) and overall survival (OS). EFS and OS were assessed in arm A (**a**, **c**) and arm B (**b**, **d**) in the full analysis population

As of June 1, 2023, the median follow-up duration was 22.4 months (95% CI 19.0–26.0). As shown in Fig. 2a, b, the median EFS was not reached (NR) (95% CI 13.6-NR), with the 6- and 12-month rates of 80.0% (95% CI 60.8–90.5) and 76.7% (95% CI 57.2–88.1) in arm A; in arm B, the corresponding values were 16.8 months (95% CI 8.6-NR), 85.7% (95% CI 62.0–95.2) and 60.7% (95% CI 36.5–78.1). At the data cutoff date, the median overall survival (OS) was immature, with respective 10 and 7 deaths in arms A and B (Fig. 2c, d). The 12- and 24-month OS rates were 80.0% (95% CI 60.8–90.5) and 65.2% (95% CI 44.7–79.7) in arm A as well as 71.1% (95% CI 46.6–85.9) and 65.2% (95% CI 40.2–81.8) in arm B. Furthermore, post-hoc analyses showed that a trend toward improved EFS and OS was observed in the surgical population when compared with the non-surgical population in both arms (Supplementary Figs. 3 and 4).

### Safety

The neoadjuvant treatment-related adverse events (TRAEs) are listed in Table 3. In arm A, 24 (80.0%) of the 30 patients experienced at least one TRAEs of any grade, of which TRAEs of grade 3 or higher were observed in eight (26.7%) patients, mainly neutrophil count decreased (five [16.7%]), platelet count decreased (three [10.0%]), anemia and white blood cell count decreased (two [6.7%] each). In arm B, TRAEs occurred in 18 (85.7%) of the 21 patients, and the TRAEs of grade 3 or higher were reported in three (14.3%) patients, including alanine aminotransferase increased (two [9.5%]), aspartate aminotransferase increased and blood bilirubin increased (one [4.8%] each). Immune-related AEs (irAEs) occurred in eight (26.7%) patients in arm A and seven (33.3%) patients in arm B, of which most events were grade 1–2 and grade 3 or higher irAEs were reported in one (3.3%) and two (9.5%) patients, respectively (Supplementary Table 3). Reactive cutaneous capillary endothelial proliferation, as a common irAE associated with camrelizumab, was observed in one (3.3%) patient in arm A and five (23.8%) patients in arm B, respectively, all of which were of grade 1/2 in severity.

**Table 3:** Treatment-related adverse events

Events, *n* (%)^a^	Arm A (*n* = 30)		Arm B (*n* = 21)	
	Any grade	Grade ≥ 3	Any grade	Grade ≥ 3
Any	24 (80.0)	8 (26.7)	18 (85.7)	3 (14.3)
White blood cell count decreased	10 (33.3)	2 (6.7)	0	0
Anemia	9 (30.0)	2 (6.7)	2 (9.5)	0
Alanine aminotransferase increased	9 (30.0)	1 (3.3)	3 (14.3)	2 (9.5)
Neutrophil count decreased	8 (26.7)	5 (16.7)	1 (4.8)	0
Platelet count decreased	8 (26.7)	3 (10.0)	0	0
Gamma-glutamyltransferase increased	8 (26.7)	1 (3.3)	3 (14.3)	0
Aspartate aminotransferase increased	5 (16.7)	0	3 (14.3)	1 (4.8)
Alkaline phosphatase increased	3 (10.0)	1 (3.3)	1 (4.8)	0
Pruritus	2 (6.7)	0	1 (4.8)	0
Proteinuria	2 (6.7)	0	2 (9.5)	0
Lymphocyte count decreased	2 (6.7)	0	0	0
Reactive cutaneous capillary endothelial proliferation	1 (3.3)	0	5 (23.8)	0
Blood bilirubin increased	1 (3.3)	0	1 (4.8)	1 (4.8)
Autoimmune hepatitis	1 (3.3)	1 (3.3)	0	0
Hyperuricemia	0	0	3 (14.3)	0
Blood glucose increased	0	0	3 (14.3)	0
Blood lactate dehydrogenase increased	0	0	2 (9.5)	0

^a^Data presented for treatment-related adverse events (TRAEs) occurring in at least 5% of patients, or any TRAEs of grade 3 or above in either arm

Two (6.7%) patients in arm A discontinued any treatment component due to AEs: one discontinued camrelizumab because of grade 4 autoimmune hepatitis and one discontinued gemcitabine due to grade 4 platelet count decreased. In arm B, one patient who experienced a second occurrence of hemoptysis (grade 1) discontinued neoadjuvant treatment because of concern for increased risk of bleeding. No treatment-related deaths occurred in either arm.

### Exploratory biomarker analyses

**Fig. 3:**
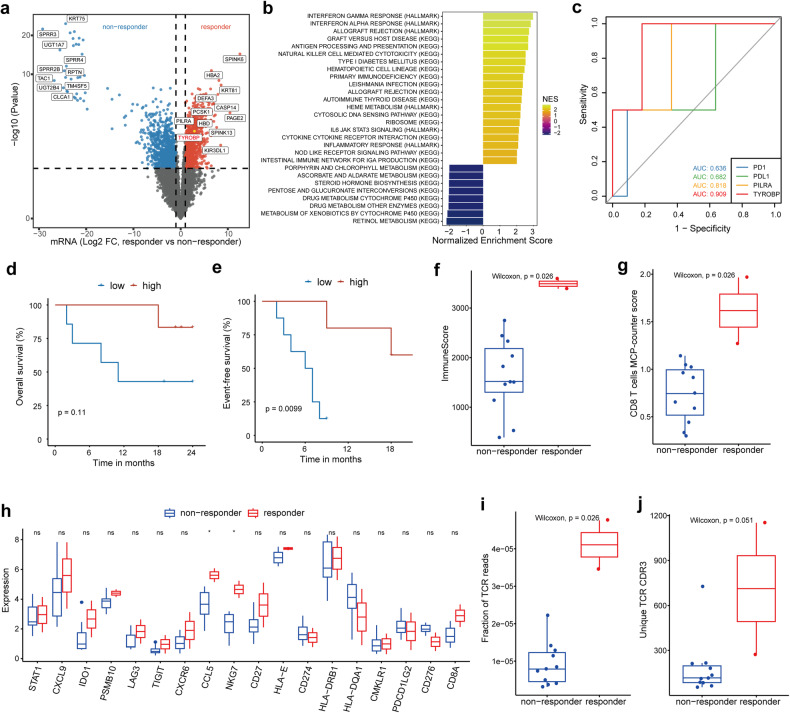
Transcriptomic analysis of baseline tumor samples in arm B. Differential expression analysis (**a**) and GSEA (**b**) in tumor samples from responders (*n* = 2) and non-responders (*n* = 11). **c** Receiver operating characteristic plots of the predictive values of TYROBP and PILRA expression for response to neoadjuvant treatment. Patients with high TYROBP expression presented with better OS (**d**) and EFS (**e**). **f** Boxplot showing immunescore in baseline tumor samples from responders versus non-responders. **g** Increasing CD8 + T cells in baseline tumor samples from responders. **h** Increasing T cell effectors’ expression in baseline tumor samples from responders. Boxplots showing fraction of TCR reads (**i**) and unique TCR CDR3 (**j**) in baseline tumor samples from responders versus non-responders. Two-sided Wilcoxon rank sum test was used for comparison in figure (**f**–**j**). *P < 0.05; **P < 0.01; ***P < 0.001. ns, not significant

There were 13 patients in arm B who had baseline tumor specimens. We performed differentially expressed gene analysis in arm B and found 2250 genes to be differentially expressed (1103 higher in responders, and 1147 in non-responders) between responders (*n* = 2) and non-responders (*n* = 11) (Fig. 3a). Of these, TYROBP and PILRA were significantly over-expressed (*P* < 0.001) in responders (Fig. 3a), with good predictive power for treatment response (area under the curve [AUC] = 0.909 and 0.818, respectively) compared with PD-1 and PD-L1 (Fig. 3c). According to the AUC value, TYROBP was selected for further analysis, and it was found that patients with higher TYROBP expression also showed a better OS (*P* = 0.11) and EFS (*P* = 0.0099) in arm B (Fig. 3d, e). Furthermore, when validated in another immunotherapeutic datasets for lung cancer, baseline TYROBP expression was higher in responders and could predict immunotherapy efficiency in GSE207422 (Supplementary Fig. 5a, b), GSE111414 (Supplementary Fig. 5c, d), GSE126044 (Supplementary Fig. 5e, f) and GSE135222 (Supplementary Fig. 5g, h).^23–26^ What’s more, patients with higher TYROBP expression showed better PFS (*P* = 0.0085) in GSE135222 (Supplementary Fig. 5i). In a recent large cohort for NSCLC receiving immune checkpoint blockades,^27^ higher TYROBP expression tendency was also observed in responders (Supplementary Fig. 5j). Meanwhile, patients with higher TYROBP expression also showed better OS (*P* = 0.0016) and PFS (*P* = 0.00076) in Patil’s cohort (Supplementary Fig. 5k, l).

Gene set enrichment analysis (GSEA) identified several immune-related processes, including “interferon gamma response”, “NK cell-mediated cytotoxicity”, and “antigen processing and presentation”, which were significantly enriched specifically in responders in arm B (Fig. 3b). In addition, high immune score was also observed and CD8 + T cells showed increasing infiltration in tumor samples from the responders (Fig. 3f, g). As for multiple T cell related genes, we found that CCL5 and NKG7 were highly expressed in responders (Fig. 3h). In the analysis of immune repertoire, we found fraction of TCR reads, and unique TCR complementarity-determining region 3 (CDR3) were higher in responders (Fig. 3i, j).

## Discussion

At present, there are no uniform boundaries for potentially resectable and unresectable NSCLC, and the diagnosis often highly relies on the imaging and the experience of the local thoracic physicians, with the consequence of possible variation in therapeutic recommendations between hospitals.^28,29^ The substantial benefits of neoadjuvant chemoimmunotherapy for resectable stage III NSCLC have triggered clinicians to consider whether neoadjuvant combination immunotherapy could convert initially unresectable NSCLC into resectable, thereby providing further benefit to patients.^14,30,31^ In this study, camrelizumab combined with platinum-doublet chemotherapy resulted in 50.0% of patients with initially unresectable stage II–III NSCLC irrespective of PD-L1 expression becoming amenable to surgical resection, with the MPR and pCR rates of 20.0% (95% CI 4.3–48.1). Additionally, camrelizumab plus apatinib led to 42.9% of patients with PD-L1 positive disease undergoing surgery, with the MPR and pCR rates of 55.6% (95% CI 21.2–86.3) and 11.1% (95% CI 0.3–48.2), respectively. To our knowledge, this was the first prospective trial aiming to assess immunotherapy combined with chemotherapy or antiangiogenic agents as neoadjuvant therapy for patients with initially unresectable stage II–III NSCLC and providing preliminary evidence for the application of neoadjuvant camrelizumab combined with platinum-doublet chemotherapy or apatinib in this population.

Previous neoadjuvant immunotherapy-based trials mainly targeted resectable NSCLC, and also changed the paradigm of neoadjuvant treatment for resectable disease. In this context, Liang et al. suggested that neoadjuvant immunotherapy could be considered for initially unresectable locally advanced NSCLC, and that the possibility of surgery could be reassessed following downgrading of tumor staging.^32^ However, the available evidence to support this is extremely scarce. Deng et al. retrospectively reported that 31 patients with initially unresectable NSCLC successfully achieved tumor downstaging with neoadjuvant chemoimmunotherapy and underwent subsequent surgery, with 67.7% of patients achieving an MPR.^18^ In LungMate 002 study, of the 38 patients with initially unresectable stage II–III NSCLC, 24 (63.2%) were successfully converted to resectable disease after neoadjuvant toripalimab plus chemotherapy and ultimately underwent surgery, with all achieving R0 resection.^33^ Unfortunately, this study did not report pathological outcomes in this population. In our study, 50.0% of the patients with initially unresectable disease in arm A and 42.9% of such patients in arm B successfully underwent surgery, and all also had an R0 resection. Additionally, our neoadjuvant combination therapy for this population achieved both MPR and pCR rates of 20.0% in arm A and the corresponding values of 55.6% and 11.1% in arm B, respectively. These findings preliminarily demonstrated the feasibility of neoadjuvant camrelizumab plus chemotherapy or apatinib in an initially unresectable population, but we should acknowledge that the patient population was too small to warrant sensitive findings.

It has been controversial whether surgery should serve as a therapeutic modality for unresectable stage III NSCLC after down-staging with initial treatment. For patients with unresectable stage II/III NSCLC, durvalumab after definitive concurrent chemoradiotherapy as a standard consolidation therapy yields satisfactory efficacy, with a median PFS of 16.8 months and the 12-month PFS rate of 55.9%, as reported by the PACIFIC study.^15^ Based on these results, Moghanaki et al. believed that in the context of favorable outcomes with nonsurgical management, patients with unresectable NSCLC should not be recommended neoadjuvant immunotherapy followed by surgery for safety concerns.^30^ Due to a higher burden of unresectable disease, even though neoadjuvant immunotherapy may decrease the tumor burden, it does not usually attenuate the demand for aggressive surgery (e.g., right total pneumonectomy) and may lead to a higher risk of major surgical complications.^30^ On the contrary, Dickhoff and his colleagues suggested that unresectable stage III NSCLC are often refractory to treatment in the event of local recurrence, whereas conversion to resectable disease may be possible with effective initial (neoadjuvant) treatment, and surgery may further improve patient survival by increasing local control rates.^31^ In our study, promising survival results were observed in patients with initially unresectable stage II–III, with the median EFS of NR in arm A and 16.8 months in arm B as well as the 12-month EFS rates of 76.7% and 60.7%. Additionally, only two patients in arm A and one in arm B developed pneumonectomy, and the incidence of postoperative complications was also low (20.0 and 0%, respectively), with no patients experiencing death within 90 days of surgery. These suggested that patients with initially unresectable stage II–III NSCLC appear to safely undergo surgery after neoadjuvant camrelizumab plus platinum-doublet chemotherapy or apatinib.

Regarding potential predictive markers for the efficacy of camrelizumab plus apatinib, we identified TYROBP as a promising marker for predicting treatment response. Currently, TYROBP, also known as DAP12, has been studied in various cancers.^34–36^ Functionally, TYROBP encodes a transmembrane signaling polypeptide on the surface of a variety of immune cells that contains an immunoreceptor tyrosine-based activation motif in its cytoplasmic domain and mediates signaling transductions.^37,38^ It has been reported that TYROBP expression is associated with CD8 T cell infiltration in gastric cancer and clear cell renal cell carcinoma.^34,35^ Previous studies also revealed the crucial role of TYROBP in NK effector responses, and its dysregulation could critically impact NK cell function.^38,39^ TYROBP was significantly over-expressed in tumor samples from responders and its expression presented the potential to predict the efficacy of immunotherapy in our discovery and another five immunotherapy-treated lung cancer cohorts. Overall, TYROBP might be a promising predictive marker for immunotherapy efficiency.

In this study, a combination of either camrelizumab with chemotherapy or with apatinib was safe and well tolerated, and the safety profile was mostly in line with previously reported data on single drug in NSCLC and other solid tumors.^21,22,40^ Most AEs related to camrelizumab were grade 1 or 2 in both arms, and the incidence of its common AE reactive cutaneous capillary endothelial proliferation was similar to that of other neoadjuvant studies with camrelizumab-based therapy.^40,41^ No new safety signals were identified.

This study had several limitations. Firstly, due to the exploratory nature of this study, the number of patients enrolled was small, especially in arm B, and there was a lack of a control group, which might diminish the certainty of the effects observed. Secondly, the imbalance of the enrolled population by sex, smoking, and histological type may introduce bias. Thirdly, the available pre- and post-treatment tumor samples were insufficient for the analysis of potential predictive biomarkers. Further comprehensive exploration of the molecular characterization is warranted in future studies with larger samples.

In conclusion, neoadjuvant camrelizumab plus platinum-doublet chemotherapy or apatinib showed preliminary efficacy and manageable toxicity in patients with initially unresectable stage II–III NSCLC, and this strategy may represent a novel opportunity to convert initially unresectable into resectable disease. Future larger sample studies are needed to confirm our findings.

## Methods

### Study design and participants

This was a prospective, multi-center, two-arm, non-randomized, phase 2 exploratory study done at six sites in China (NCT04379739). This study was conducted in accordance with the Declaration of Helsinki and Good Clinical Practices and was approved by the ethics committee at each site. Written informed consent was obtained from each patient before study initiation.

Patients were eligible if they were aged 18–75 years; had histologically or cytologically confirmed treatment-naïve initially unresectable stage II–III NSCLC (according to the eighth edition of the American Joint Committee on Cancer TNM staging system for lung cancer); had ECOG PS of 0 or 1; had measurable disease as per RECIST criteria version 1.1, and had adequate organ function. Initially unresectable disease was evaluated by a multidisciplinary clinical team (MDT), and was defined as (1) tumor invading vital structures, such as large blood vessels, the trachea or primary bronchus, but curative resection after tumor downgrading by neoadjuvant therapy was possible as judged by a preoperative assessment; (2) clinically confirmed lymph nodes with multistation metastasis or bulky fusion, and patients could tolerate hilar and mediastinal lymph node dissection after tumor downgrading by neoadjuvant therapy as determined by a preoperative evaluation, or (3) according to preoperative evaluation, even if pneumonectomy was performed, especially right pneumonectomy, R0 resection may not be achieved. Patients regardless of PD-L1 expression were enrolled to arm A. Patients with a baseline PD-L1 expression of 1% or higher, no uncontrolled hypertension, no thrombotic events, and no obvious bleeding tendency were enrolled to arm B. Key exclusion criteria in both arms included known EGFR mutations or ALK fusion, a history of autoimmune disease, a history of other malignancies within the past 5 years, and receiving systemic therapy with corticosteroids (>10 mg/day prednisone equivalent) or other immunosuppressive agents within 2 weeks prior to study treatment.

### Procedures

Patients in arm A received 2–4 cycles of neoadjuvant camrelizumab (200 mg on day 1) and platinum-doublet chemotherapy (squamous NSCLC: carboplatin AUC 5 on day 1 and gemcitabine 1000 mg/m^2^ on days 1 and 8 [or paclitaxel 135–175 mg/m^2^, or docetaxel 60–75 mg/m^2^, or nab-paclitaxel 260 mg/m^2^, on day 1]; non-squamous NSCLC: carboplatin AUC 5 and pemetrexed 500 mg/m^2^ on day 1) intravenously every 3 weeks. In arm B, patients received 2–4 cycles of neoadjuvant camrelizumab and apatinib (250 mg, orally, once daily) every 3 weeks.

After the first two treatment cycles, an enhanced computed tomography (CT) scan of the chest was performed. If a tumor response (complete response [CR] or PR) was achieved according to RECIST version 1.1 and surgery was assessed as safe and feasible by an MDT, surgery was planned within 30 days of tumor assessment; otherwise, the cycle 3 of neoadjuvant treatment was continued. The determination of whether to continue the cycle 4 of treatment was similar to that of the previous cycle. If patients could not tolerate all four cycles of neoadjuvant therapy, they could also undergo surgery in advance. Before surgery, a radiographic evaluation with enhanced CT and magnetic resonance imaging was performed 3–4 weeks after the last dose of treatment; and then, the MDT conducted a comprehensive assessment based on the location of the tumor, the extent of tumor invasion, lymph node status and the physical conditions to identify the resectability of the tumor. Pathological response was evaluated in accordance with the previous description.^42^ Patients had a postoperative evaluation with a chest CT scan at 30 days after surgery, and then again every 3 months for the first two years and every 6 months for the third to fifth years. AEs were continuously monitored throughout the trial and graded according to the National Cancer Institute Common Terminology Criteria for Adverse Events version 5.0. The PD-L1 tumor proportion score at baseline was measured by the 22C3 pharmDx assay (Agilent Technologies, CA, USA).

### Endpoints

The primary endpoint was MPR rate, defined as the proportion of patients with 10% or less residual viable tumor cells at resected primary tumor. Secondary endpoints included pCR rate (defined as the proportion of patients with no residual viable tumor cells at resected primary tumor), ORR (defined as the proportion of patients with CR or PR as per RECIST version 1.1), EFS (defined as the time from the initiation of neoadjuvant treatment to the first occurrence of disease progression that precluded surgery disease, local or distant recurrence, or death from any cause), 1- and 2-year EFS rate, DFS (defined as the time from surgery to disease recurrence or death from any cause), 1- and 2-year DFS rate, OS (defined as the time from the initiation of neoadjuvant treatment to death from any cause), R0 resection rate (defined as the proportion of patients with margin-negative resection), and safety.

### RNA sequencing

Total RNA from fresh frozen tissues was extracted with TRIzol. Sequencing libraries were generated using a NEBNext Ultra RNA Library Prep Kit for Illumina, and index codes were added to attribute sequences to each sample. The libraries were pooled, and paired-end sequencing (2 × 150 bp reads) was performed using an Illumina NovaSeq 6000. After RNAseq sequencing, raw fastq files were trimmed via fastp and aligned to GRCh38 reference genome by the Spliced Transcripts Alignment to a Reference (STAR) with default settings.^43,44^ After obtaining the BAM files, read counts were summarized by featureCounts, and TPMs (Trans Per Million) were generated using Salmon.^45,46^ Batch effects were adjusted using “combat” function in sva package.

### Differentially expressed gene analysis

We used DESeq2 to calculate differential gene expression between sample groups.^47^ The DESeq2 profiles genes according to model gene count expression data and calculates log2-fold change, which estimates the effect size and represents gene changes between comparison groups. The two-sided Wald-test statistics were computed to exam the differential expression across the comparison groups. Genes with |log2-fold change | > 1 and Wald-test *P* < 0.05 were defined as differentially expressed genes. We used volcano plots to visualize the differential gene expression results.

### Biomarker selection

We focused on genes up-regulated in responder group (log2FC > 1 & *P* < 0.05) via deseq2 and with prognostic value (HR < 1 & Log rank *P* < 0.05) in the discovery dataset. Multiple immunotherapy-treated lung cancer datasets were then applied to assist the analysis, focusing on genes up-regulated in responder group in GSE207422, GSE126044, and GSE135222. The results from the discovery dataset and from published datasets were combined, with two genes “TYROBP” and “PILRA” selected. The AUC values for these two genes in the discovery dataset were calculated, and the final biomarker for analysis was selected based on the highest AUC value.

### Gene set enrichment analysis

For GSEA, results for all protein coding genes were ranked by log2-fold change and evaluated with the “GSEA” algorithm. “Hallmark” and “KEGG” gene sets were acquired from MSigDb. We filtered GSEA results based on the criterion of *P* < 0.05 and visualized the candidate pathways based on the normalized enrichment score.

### Tumor micro-environment estimation

The immunescore of each sample was calculated via the “Estimation of STromal and Immune cells in MAlignant Tumors using Expression data (ESTIMATE)” R package.^48^ The infiltration of immune cells was evaluated by “Microenvironment Cell Populations-counter (MCP-counter)”.^49^ For immune repertoire analysis, TRUST4 algorithm was applied to evaluate the immune repertoire and to extract T and B cell receptors (TCR and BCR, respectively) CDR3 sequences.^50^

### Statistical analysis

Since the pathological response of patients with initially unresectable NSCLC was unknown, no formal sample size calculation was performed for our study. Regarding the exploratory nature of this study, a minimum sample size of 20 patients per arm were determined.

Response-related endpoints were assessed in the efficacy analysis population, which consisted of all patients who received at least one dose of neoadjuvant treatment and underwent surgery. Survival and safety analyses were done in the full analysis population, which comprised all patients who received at least one dose of neoadjuvant treatment. The 95% CIs of MPR rate, pCR rate, ORR, and R0 resection rate were estimated by means of the Clopper-Pearson method. Time-to-event analyses were estimated with the Kaplan–Meier method, with median survival time presented with 95% CIs based on the Brookmeyer-Crowley method and survival rates presented along with 95% CIs based on the complementary log-log transformation method. TRAEs were summarized with frequencies (percentages). Additionally, EFS and OS stratified by surgery were explored by post-hoc analyses. Wilcoxon rank sum test was applied to compare the expression differences between sample groups. To separate patients into low- or high- TYROBP groups, the cutoff was generated based on the association between TYROBP expression and survival data using the survminer package. All reported *P* values were two-sided, with *P* < 0.05 considered statistically significant. Statistical analyses were done using SAS software (version 9.4; SAS Institute, Inc., Cary, North Carolina) and R 4.1.0.

### Supplementary information

Supplementary Materials

Supplementary Information for Resectability Before and After Neoadjuvant Therapy

Protocol

## Data Availability

We deposited the RNA-seq data in the Genome Sequence Archive database under accession number HRA005439. Only open-source software was used for this study, and no custom codes were generated for RNA-seq analysis. Deidentified individual data are available from the corresponding author on reasonable request.
