# Supplementary material for: Neoadjuvant camrelizumab (an anti-PD-1 antibody) plus chemotherapy or apatinib (a VEGFR-2 inhibitor) for initially unresectable stage II–III non-small-cell lung cancer: a multicentre, two-arm, phase 2 exploratory study
Source: Signal Transduct Target Ther. 2024 Jun 14;9:145. doi: 10.1038/s41392-024-01861-w (PMC11176298; doi:10.1038/s41392-024-01861-w)
Supplement: Supplementary file 1 — Supplementary Materials [file 41392_2024_1861_MOESM1_ESM.docx]

Supplementary Materials for

Neoadjuvant Camrelizumab Plus Chemotherapy or Apatinib for Initially Unresectable Stage II-III Non-small-cell Lung Cancer: A Multicentre, Two-arm, Phase 2 Exploratory Study

Haoran Xia, Han Zhang, Zheng Ruan, Huibiao Zhang, Liangdong Sun, Hezhong Chen, Yongxin Zhou, Lele Zhang, Dongliang Bian, Xinsheng Zhu, Jing Zhang, Fenghuan Sun, Huansha Yu, Nan Song, Xiaogang Liu, Yuming Zhu, Haiping Zhang, Wenxin He, Jian Chen, Jie Yang, Guohan Chen, Shiliang Xie, Dongfang Tang, Xiaomiao Zhang, Liang Duan, Deping Zhao, Qinchuan Li, Peng Zhang, Gening Jiang

Correspondence to: Deping Zhao ([zdp1992@163.com](mailto:zdp1992@163.com)), Qinchuan Li ([li.qinchuan@163.com](mailto:li.qinchuan@163.com)), Peng Zhang ([zhangpeng1121@tongji.edu.cn](mailto:zhangpeng1121@tongji.edu.cn)) and Gening Jiang ([jgnwp@aliyun.com](mailto:jgnwp@aliyun.com))

**This file includes:**

Figures S1 to S5

Tables S1 to S3


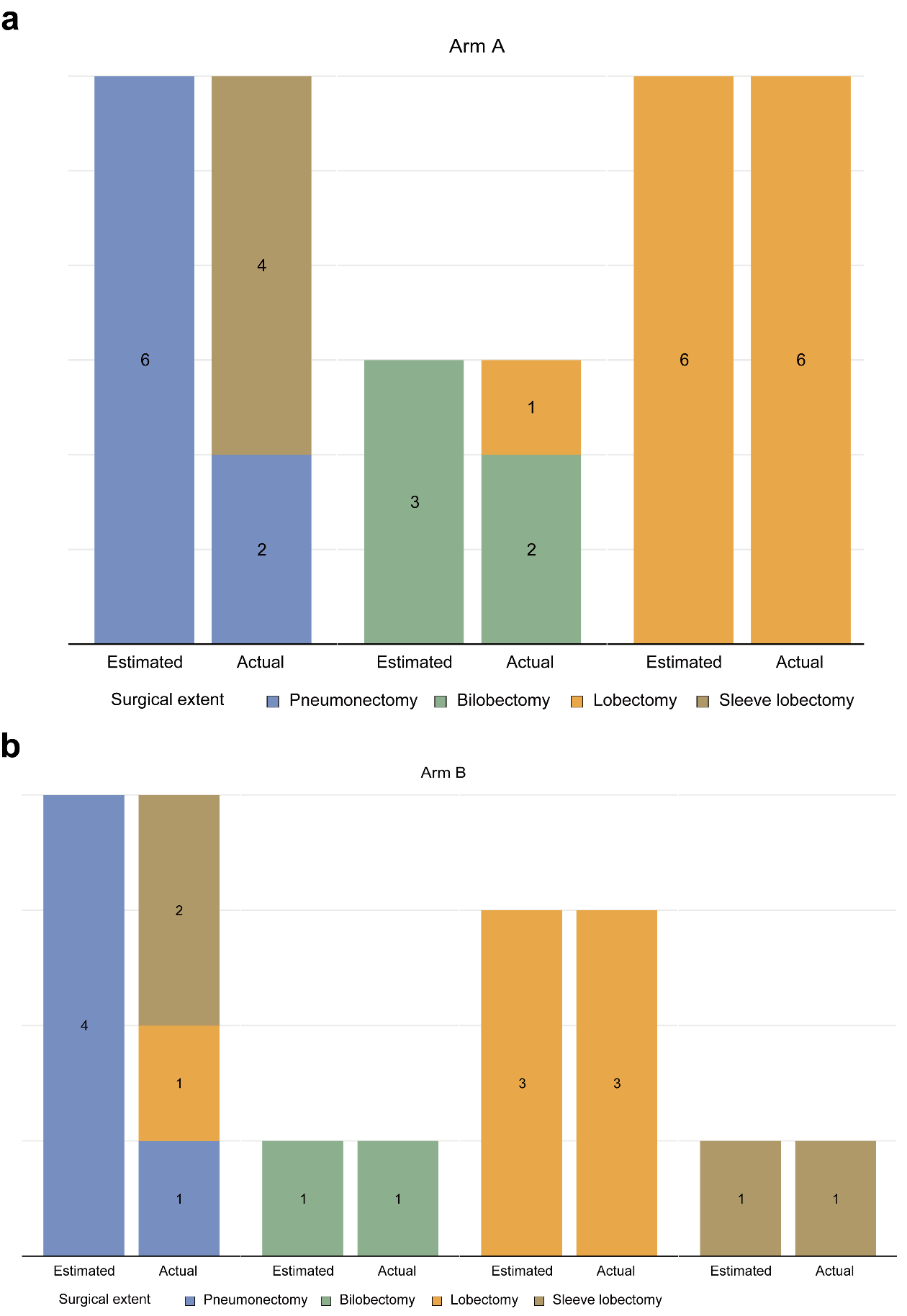


**Figure S1. Estimated surgical method at baseline and actual surgical method after neoadjuvant therapy** **in arm A (a) and arm B (b).**

**
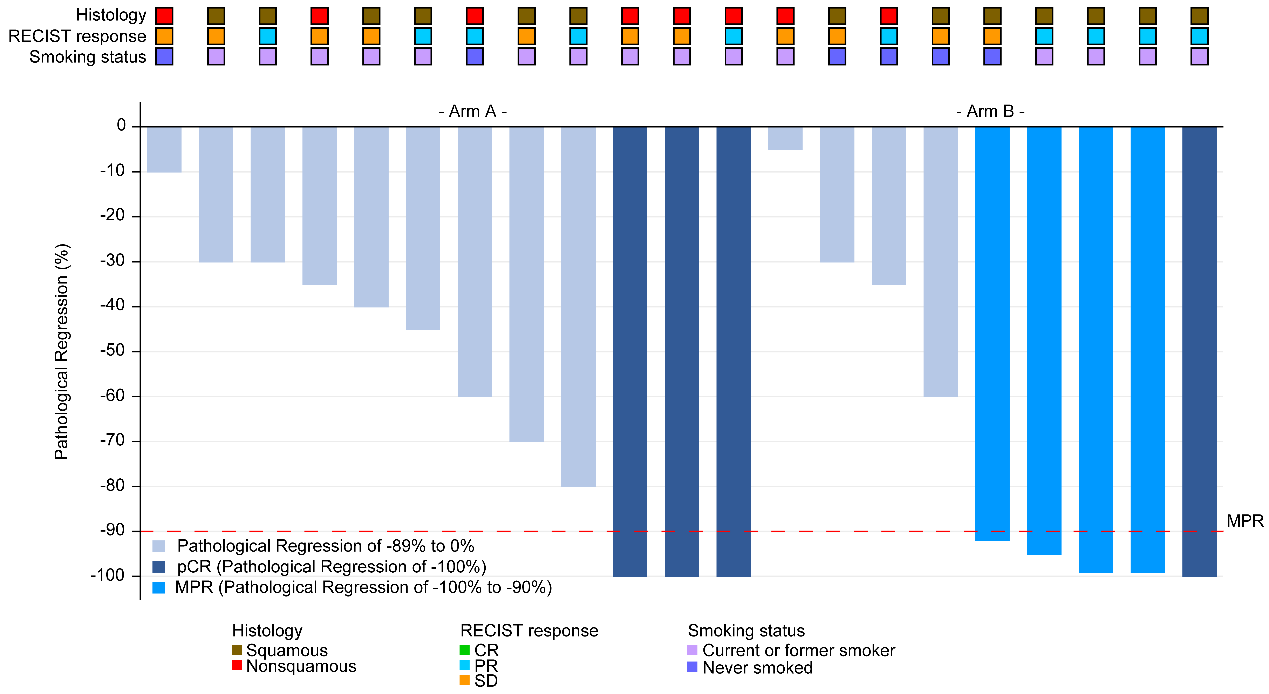
**

**Figure S2. Waterfall plot.** Three patients from other places in arm A were unable to return to our hospital for surgery due to the COVID-19 pandemic and opted for surgery locally instead, resulting in unavailable pathological regression results. CR, complete response; MPR, major pathological response; pCR, pathological complete response; PR, partial response; RECIST, Response Evaluation Criteria in Solid Tumors; SD, stable disease.

**
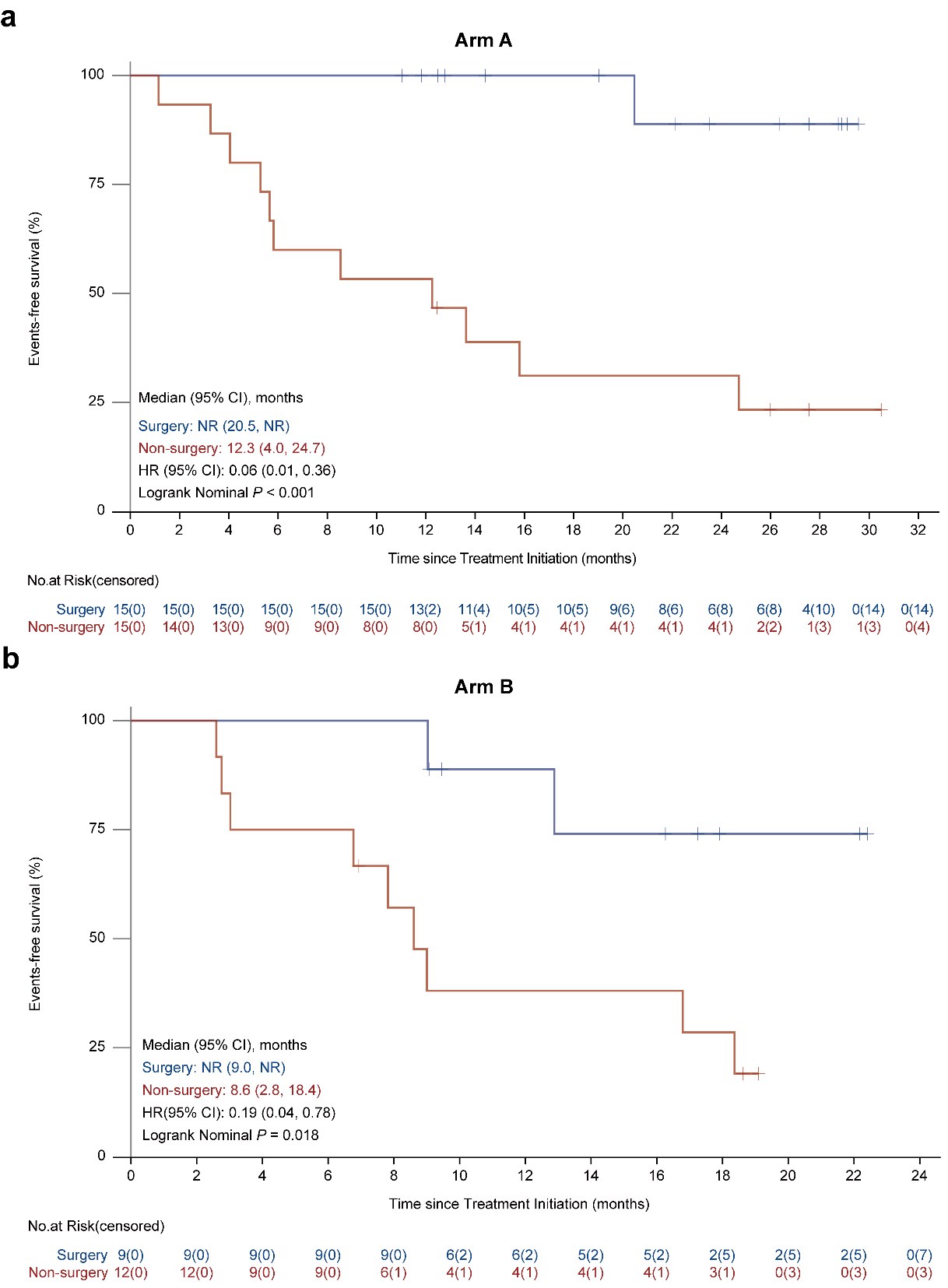
**

**Figure S3: Kaplan-Meier curves for event-free survival according to surgery in arm A (a) and arm B (b).** CI, confidence interval; HR, hazard ratio; NR, not reached.


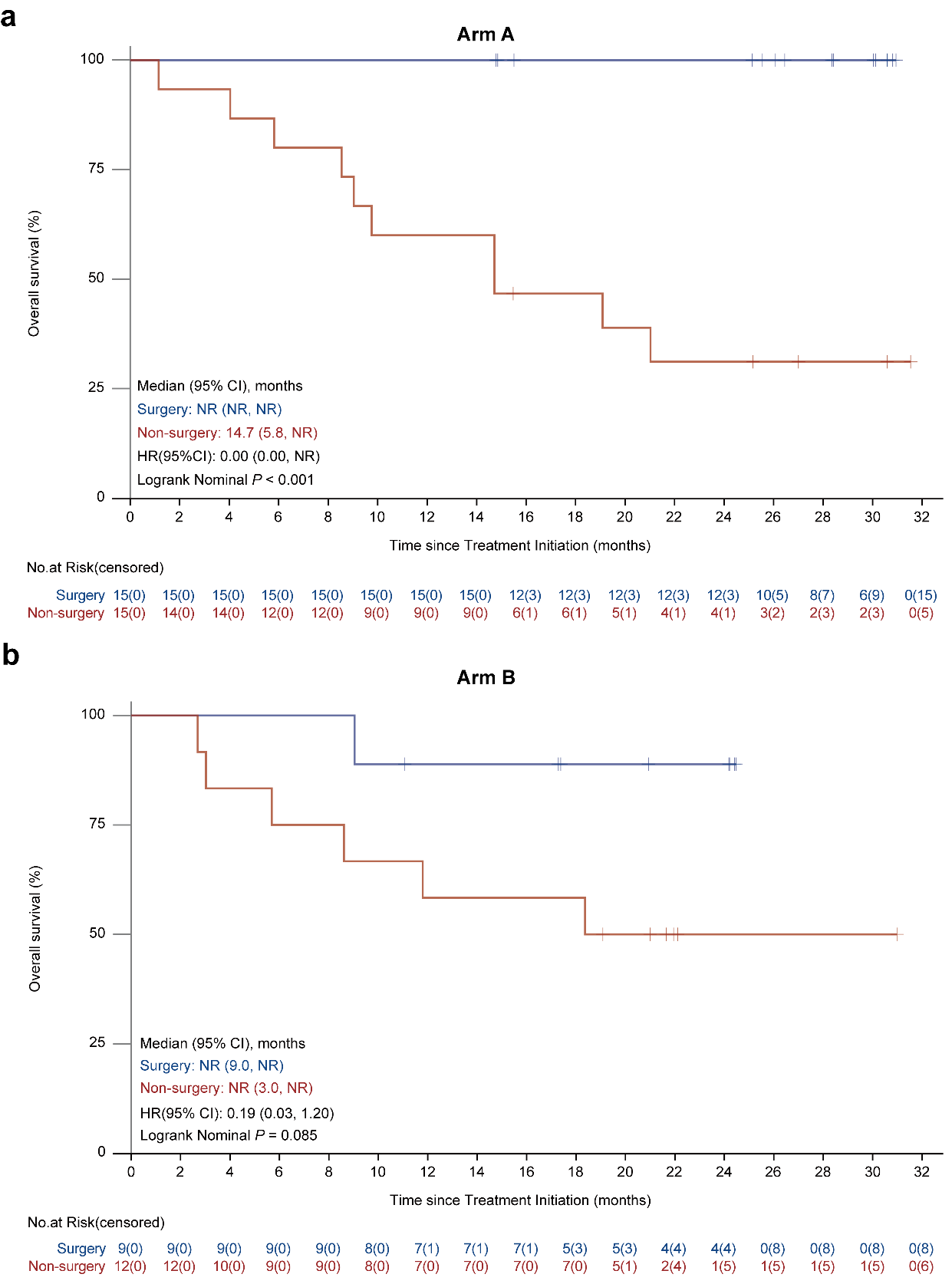


**Figure S4: Kaplan-Meier curves for overall survival according to surgery in arm A (a) and arm B (b).** CI, confidence interval; HR, hazard ratio; NR, not reached.

**
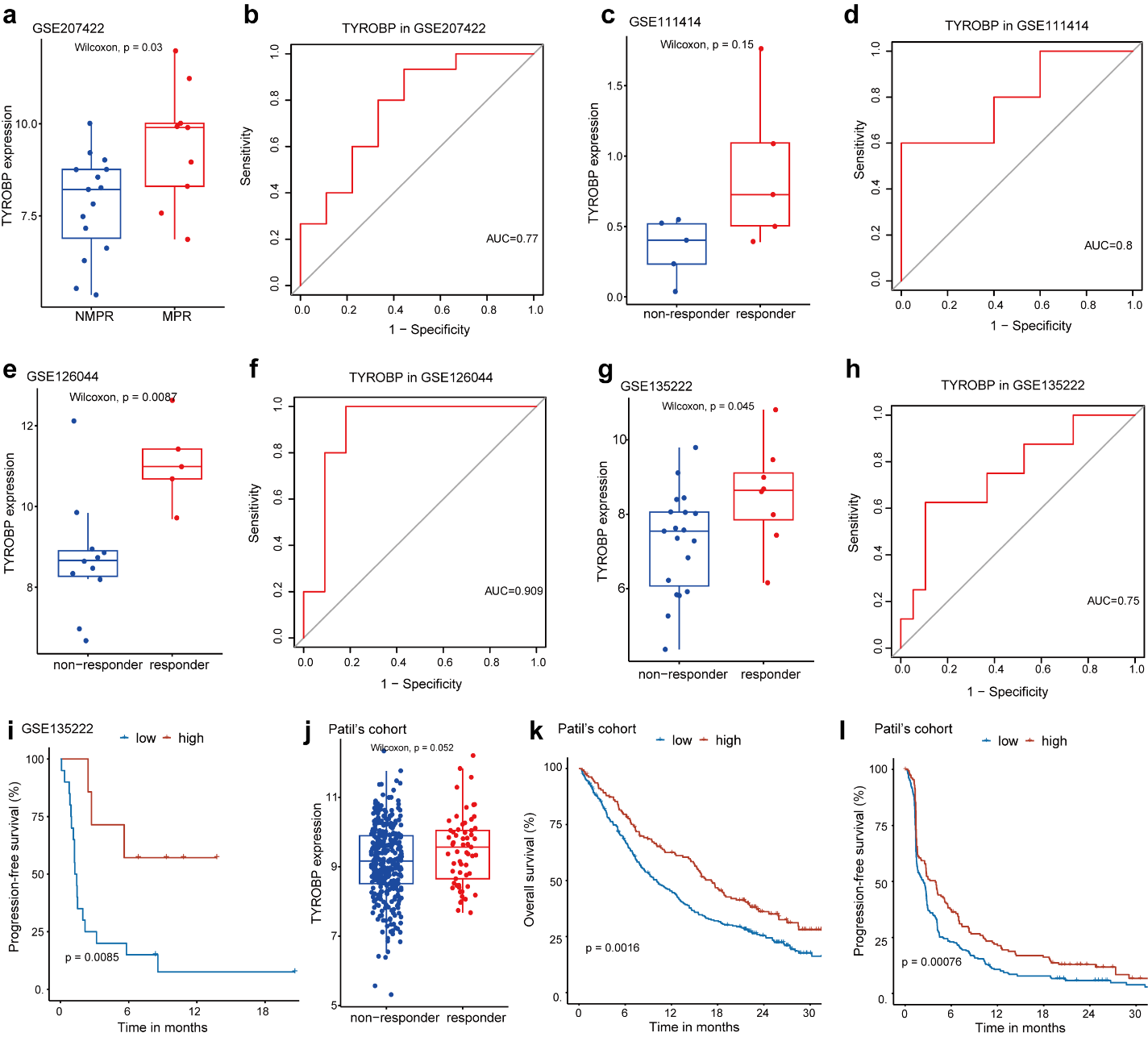
**

**Figure S5. Validation of TYROBP in another immune checkpoint blockade cohorts for lung cancer.** **(a, c, e, g, j)** Boxplots showing TYROBP expression level in baseline tumor samples from responders versus non-responders in GSE207422 (**a**), GSE111414 (**c**), GSE126044 (**e**), GSE135222 (**g**), and Patil’s cohort (**j**). **(b, d, f, h**) The predictive values of TYROBP expression for response to immunotherapy (AUC) were 0.77 in GSE207422 (**b**), 0.8 in GSE111414 (**d**), 0.909 in GSE126044 (**f**), and 0.75 in GSE135222 (**h**), respectively. (**i**) The difference in PFS between high - and low- TYROBP expression groups in GSE135222. The difference in OS (**k**) and PFS (**l**) between high - and low- TYROBP expression groups in Patil’s cohort. PFS, progression-free survival; OS, overall survival.

**Table S1. Surgical outcomes**

|  | **Arm A**  **(*n* = 15)** | **Arm B**  **(*n* = 9)** |
| --- | --- | --- |
| Surgery rate, *n*/N (%) | 15/30 (50.0) | 9/21 (42.9) |
| R0 resection rate, % (95% CI) | 100.0 (78.2-100.0) | 100.0 (66.4-100.0) |
| Delayed surgery, *n* (%) | 3 (20.0) | 2 (22.2) |
| Surgical approach, *n* (%) |  |  |
| Video-assisted thoracoscopic surgery | 9 (60.0) | 7 (77.8) |
| Thoracotomy | 6 (40.0) | 2 (22.2) |
| Type of surgery, *n* (%) |  |  |
| Lobectomy | 7 (46.7) | 4 (44.4) |
| Sleeve lobectomy | 4 (26.7) | 3 (33.3) |
| Pneumonectomy | 2 (13.3) | 1 (11.1) |
| Bilobectomy | 2 (13.3) | 1 (11.1) |
| Operative time (hours), median (range) | 2.5 (1.7-4.7) | 3.0 (1.5-4.0) |
| Estimated blood loss (mL), median (range) | 75 (30-1300) | 100 (20-800) |
| Intraoperative blood transfusion, *n* (%) | 1 (6.7) | 1 (11.1) |
| Drainage tube removal time (days), median (range) | 12.5 (5-31) | 7 (4-12) |
| Length of hospital stay after surgery (days), median (range) | 12 (6-51) | 10 (6-14) |
| Downstaging of nodal status in patients with N2 at baseline, *n* (%) |  |  |
| N2 to N0 | 2 (13.3) | 1 (11.1) |
| N2 to N1 | 1 (6.7) | 1 (11.1) |
| N2 to N2 | 3 (20.0) | 4 (44.4) |
| Surgical complications, *n* (%) | 3 (20.0) | 0 |

**Table S2. Response rates in intention to treat patients**

| **Efficacy outcomes** | **Arm A (*n* = 30)** | **Arm B (*n* = 21)** |
| --- | --- | --- |
| **Pathological response, *n* (%, 95% CI)** |  |  |
| Major pathological response | 3 (10.0, 2.1-26.5) | 5 (23.8, 8.2-47.2) |
| Pathological complete response | 3 (10.0, 2.1-26.5) | 1 (4.8, 0.1-23.8) |
| **Best overall response, *n* (%)** |  |  |
| Complete response | 0 | 0 |
| Partial response | 8 (26.7) | 7 (33.3) |
| Stable disease | 18 (60.0) | 13 (61.9) |
| Progressive disease | 0 | 1 (4.8) |
| Not evaluable | 4 (13.3) | 0 |
| **Objective response rate, % (95% CI)** | 26.7 (12.3-45.9) | 33.3 (14.6-57.0) |

**Table S3. Immune-related adverse events**

| **Events, *n* (%)** | **Arm A (*n* = 30)** | | **Arm B (*n* = 21)** | |
| --- | --- | --- | --- | --- |
|  | **Any grade** | **Grade ≥3** | **Any grade** | **Grade ≥3** |
| Any | 8 (26.7) | 1 (3.3) | 7 (33.3) | 2 (9.5) |
| Reactive cutaneous capillary endothelial proliferation | 1 (3.3) | 0 | 5 (23.8) | 0 |
| Aspartate aminotransferase increased | 3 (10.0) | 0 | 2 (9.5) | 1 (4.8) |
| Alanine aminotransferase increased* | 2 (6.7) | 1 (3.3) | 2 (9.5) | 2 (9.5) |
| Gamma-glutamyltransferase increased* | 2 (6.7) | 1 (3.3) | 1 (4.8) | 0 |
| Alkaline phosphatase increased* | 2 (6.7) | 1 (3.3) | 0 | 0 |
| Autoimmune hepatitis* | 1 (3.3) | 1 (3.3) | 0 | 0 |
| Hypothyroidism | 1 (3.3) | 0 | 0 | 0 |
| Hyperthyroidism | 1 (3.3) | 0 | 0 | 0 |
| Blood bilirubin increased | 1 (3.3) | 0 | 0 | 0 |

*One patient experienced grade 3 or above of aspartate aminotransferase increased, gamma-glutamyltransferase increased, and alkaline phosphatase increased, thus was diagnosed as autoimmune hepatitis.
