# Supplementary material for: Neoadjuvant camrelizumab (an anti-PD-1 antibody) plus chemotherapy or apatinib (a VEGFR-2 inhibitor) for initially unresectable stage II–III non-small-cell lung cancer: a multicentre, two-arm, phase 2 exploratory study
Source: Signal Transduct Target Ther. 2024 Jun 14;9:145. doi: 10.1038/s41392-024-01861-w (PMC11176298; doi:10.1038/s41392-024-01861-w)
Supplement: Supplementary file 2 — Supplementary Information for Resectability Before and After Neoadjuvant Therapy [file 41392_2024_1861_MOESM2_ESM.docx]

**Supplementary Information 1: The specific reasons for each patient's potential unresectable**

| **Patient Number** | **Treatment arm** | **Reasons for initial unresectability** | **Reasons for unresectability after neoadjuvant treatment** |
| --- | --- | --- | --- |
| 001 | A | Tumor invading the trachea | Assessed as incomplete resectability: tumor still invading the trachea. |
| **002** | A | Tumor invading vital structures, such as left primary bronchus and pulmonary artery | Assessed as incomplete resectability: tumor still invading the left primary bronchus. |
| 003 | A | Tumor invading the superior vena cava | - (Surgery performed) |
| **004** | A | Tumor invading the thoracic aorta | - (Surgery performed) |
| 005 | A | Tumor invading the right inferior pulmonary vein | - (Surgery performed) |
| 006 | A | Giant tumor with the invasion of the right primary bronchus and right middle and inferior pulmonary arteries, which may require a right pneumonectomy that was not tolerated by the patient and may still fail to achieve R0 resection | Assessed as incomplete resectability: tumor still invading the right superior and inferior lobar bronchus, as well as the right middle and inferior pulmonary arteries. |
| 007 | A | Multistation metastasis in mediastinal lymph nodes | Patient's decision (refusal of surgery) |
| 008 | A | Tumor invading the right primary bronchus | - (Surgery performed) |
| 009 | A | Multistation metastasis in mediastinal lymph nodes | - (Surgery performed) |
| **010** | A | Tumor invading the left primary bronchus and the left pulmonary artery, with the possibility of failure to achieve R0 resection even with left pneumonectomy. | - (Surgery performed) |
| 011 | A | Bulky structures of metastatic mediastinal lymph nodes | Assessed as incomplete resectability: giant lymph nodes. |
| 012 | A | Tumor invading the left pulmonary artery | Assessed as incomplete resectability: tumor still invading the left pulmonary artery. |
| 013 | A | Tumor invading the right pulmonary artery, with the possibility of failure to achieve R0 resection even with right pneumonectomy | Assessed as incomplete resectability: tumor still invading the right pulmonary artery. |
| 014 | A | Multistation metastasis in mediastinal lymph nodes | - (Surgery performed) |
| 015 | A | Tumor invading the right inferior pulmonary vein, with the possibility of failure to achieve R0 resection even with right pneumonectomy | Withdrawal for serious adverse events |
| 016 | A | Tumor invading the trachea and carina | - (Surgery performed) |
| 017 | A | Tumor invading the carina and right primary bronchus, was well as the left primary bronchus | Assessed as incomplete resectability: tumor still invading the carina. |
| 018 | A | Tumor invading the carina and the right primary bronchus | Assessed as incomplete resectability: tumor still invading the carina and the right primary bronchus. |
| 019 | A | Tumor invading the left pulmonary artery | Assessed as incomplete resectability: tumor still invading the left pulmonary artery. |
| 020 | A | Tumor invading trachea, carina and the right primary bronchus, with the possibility of failure to achieve R0 resection even with right pneumonectomy | Patient's decision (refusal of surgery) |
| 021 | A | Multistation metastasis in mediastinal lymph nodes with bulky structures | - (Surgery performed) |
| **022** | A | Tumor invading the left pulmonary artery | Assessed as incomplete resectability: tumor still invading the left pulmonary artery. |
| 023 | A | Tumor invading the thoracic aorta | - (Surgery performed) |
| **024** | A | Multistation metastasis in mediastinal lymph nodes with bulky structures | - (Surgery performed) |
| 025 | A | Tumor invading the left primary bronchus | - (Surgery performed) |
| 026 | B | Tumor invading the thoracic aorta, with invasion of the left primary bronchus and the left inferior pulmonary artery as well as the left superior pulmonary vein | - (Surgery performed) |
| **027** | B | The enlarged hilar lymph nodes invading left superior and inferior lobar bronchus, as well as the left superior pulmonary vein, with the possibility of failure to achieve R0 resection even with left pneumonectomy. | - (Surgery performed) |
| 028 | B | Tumor invading the left primary bronchus | - (Surgery performed) |
| 029 | B | Multistation metastasis in mediastinal lymph nodes | - (Surgery performed) |
| 030 | B | Tumor invading the left pulmonary artery | Assessed as incomplete resectability: tumor still invading the left pulmonary artery. |
| 031 | B | Tumor invading the right primary bronchus and the right pulmonary artery | Assessed as incomplete resectability: tumor still invading the right primary bronchus and the right pulmonary artery. |
| 032 | B | Tumor invading the vena cava, right primary bronchus, with the possibility of failure to achieve R0 resection even with right pneumonectomy | Withdrawal for adverse event |
| 033 | B | Tumor invading the left pulmonary artery | Inoperable for physical conditions |
| 034 | B | Tumor invading the thoracic aorta | Patient's decision (refusal of surgery) |
| **035** | B | Tumor invading the carina and the right primary bronchus | - (Surgery performed) |
| 036 | B | Tumor fused with metastatic hilar lymph node, with invasion of the right primary bronchus, with the possibility of failure to achieve R0 resection even with right pneumonectomy. | Withdrawal for adverse event |
| 037 | B | Tumor invading the primary bronchus and the vena cava | - (Surgery performed) |
| 038 | B | Tumor invading the right primary bronchus and the superior vena cava | Patient's decision (refusal of therapy) |
| 039 | B | Tumor invading the carina, with the possibility of failure to achieve R0 resection even with right pneumonectomy | Assessed as incomplete resectability: tumor still invading the carina. |
| **040** | B | Tumor invading the left pulmonary artery | Assessed as incomplete resectability: tumor still invading the left pulmonary artery. |
| 041 | B | Tumor invading the pulmonary trunk | Disease progression |
| 042 | B | Tumor invading the right primary bronchus and the superior vena cava | Assessed as incomplete resectability: tumor still invading the superior vena cava. |
| 043 | B | Multistation metastasis in mediastinal lymph nodes with bulky structures | - (Surgery performed) |
| **044** | B | Tumor invading the carina and the superior vena cava | Assessed as incomplete resectability: tumor still invading the carina. |
| **045** | B | Multistation metastasis in mediastinal lymph nodes with bulky structures | - (Surgery performed) |
| 046 | A | Tumor invading the right primary bronchus and the carina | Patient's decision (refusal of therapy) |
| 047 | A | Bulky structures of right hilar lymph nodes, with invasion of the right middle and inferior pulmonary vein | - (Surgery performed) |
| 048 | A | Tumor invading the carina and left primary bronchus | Patient's decision (refusal of surgery) |
| 049 | A | Tumor invading the left pulmonary artery | - (Surgery performed) |
| 050 | A | Multistation metastasis in mediastinal lymph nodes with bulky structures | - (Surgery performed) |
| 051 | B | Multistation metastasis in mediastinal lymph nodes with bulky structures | - (Surgery performed) |

**Supplementary Information 2: Resectability before and after neoadjuvant therapy in 10 typical cases**

| Patient Number | 002 |
| --- | --- |
| Treatment arm | A |
| PD-L1 expression | < 1% |
| Clinical stage | IIIA (cT4N0M0) |
| An enhanced computed tomography scan of the chest at baseline | 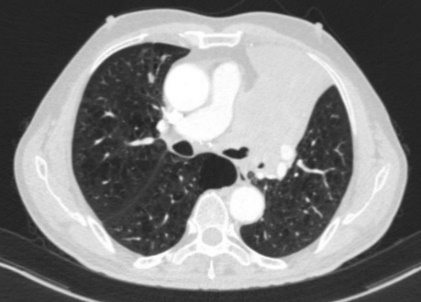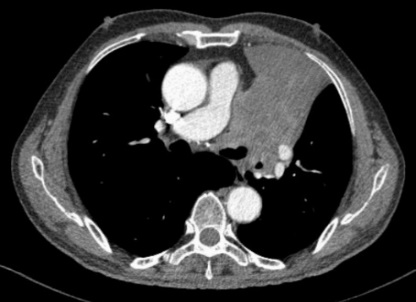 |
| Reasons for initial unresectability | Tumor invading vital structures, such as left primary bronchus and pulmonary artery |
| Number of neoadjuvant treatment cycles | 4 |
| An enhanced computed tomography scan of the chest after neoadjuvant treatment | 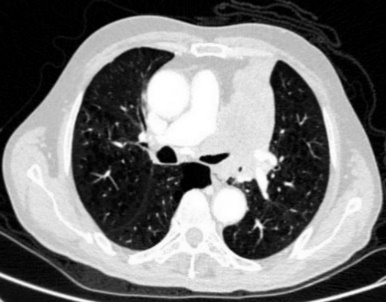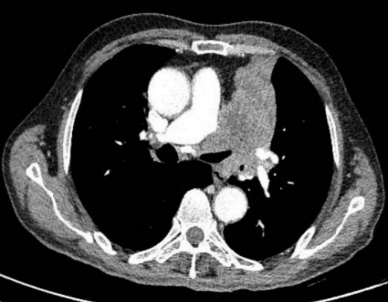 |
| Surgery | No |
| Reasons for unresectability after neoadjuvant treatment | Assessed as incomplete resectability: tumor still invading the left primary bronchus. |

| Patient Number | 004 |
| --- | --- |
| Treatment arm | A |
| PD-L1 expression | 1%-49% |
| Clinical stage | IIIA (cT4N1M0) |
| An enhanced computed tomography scan of the chest at baseline | 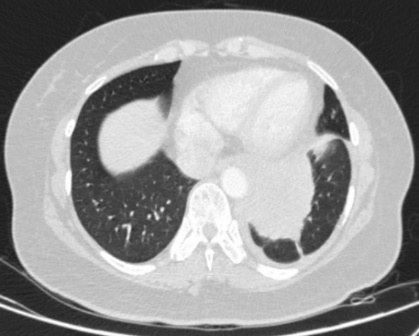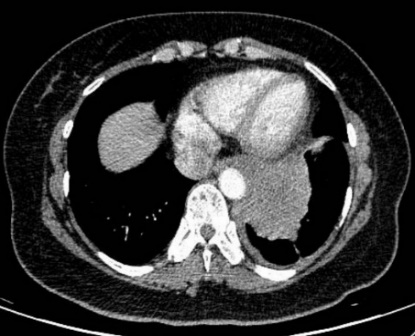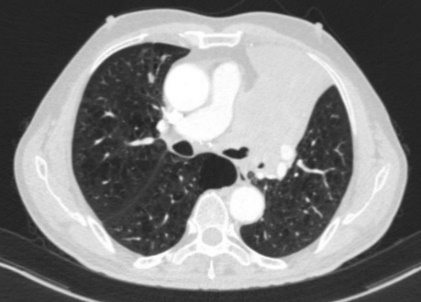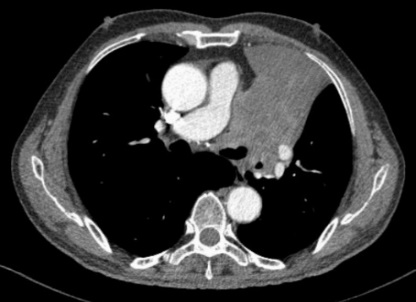 |
| Reasons for initial unresectability | Tumor invading the thoracic aorta. |
| Number of neoadjuvant treatment cycles | 3 |
| An enhanced computed tomography scan of the chest after neoadjuvant treatment | 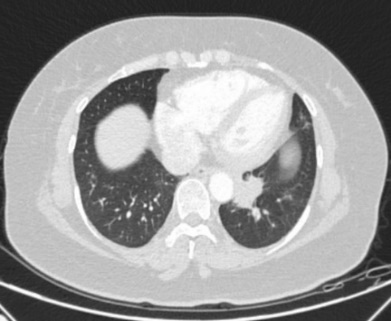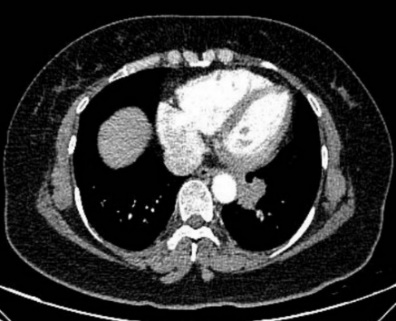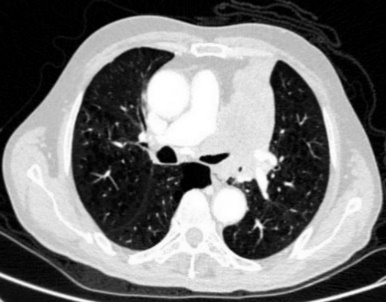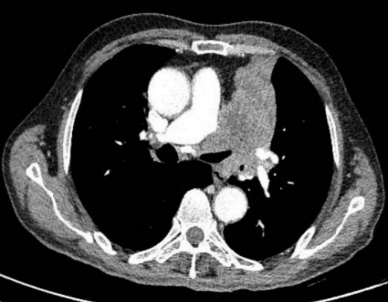 |
| Surgery | Yes |
| Reasons for unresectability after neoadjuvant treatment | - |

| Patient Number | 010 |
| --- | --- |
| Treatment arm | A |
| PD-L1 expression | < 1% |
| Clinical stage | IIIA (cT4N0M0) |
| An enhanced computed tomography scan of the chest at baseline | 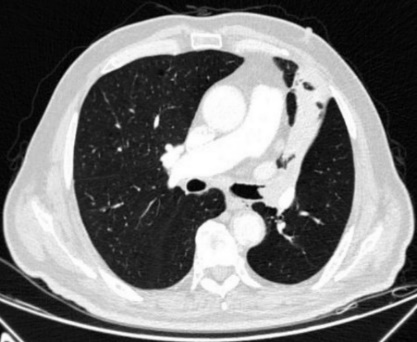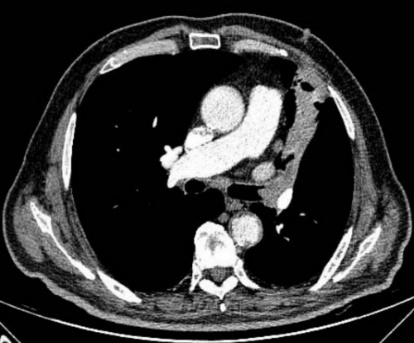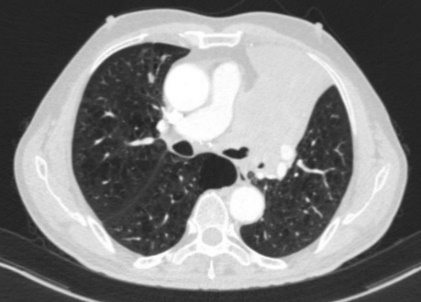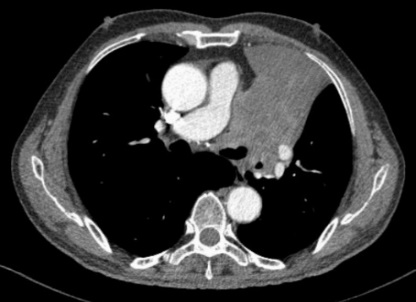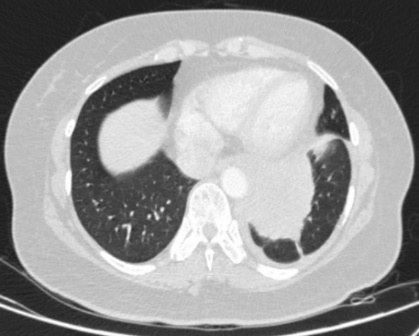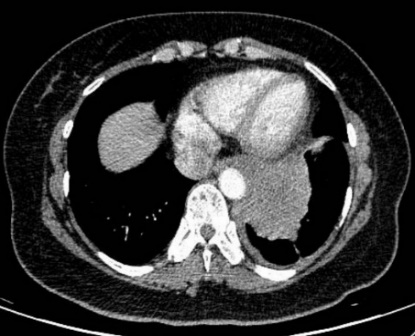  \|  \| \| --- \| |
| Reasons for initial unresectability | Tumor invading the left primary bronchus and the left pulmonary artery, with the possibility of failure to achieve R0 resection even with left pneumonectomy. |
| Number of neoadjuvant treatment cycles | 4 |
| An enhanced computed tomography scan of the chest after neoadjuvant treatment | 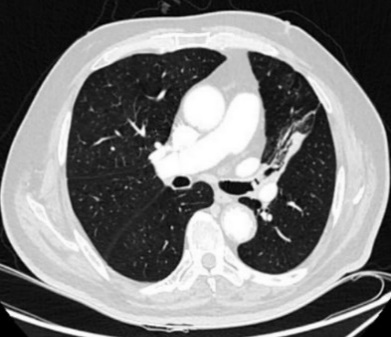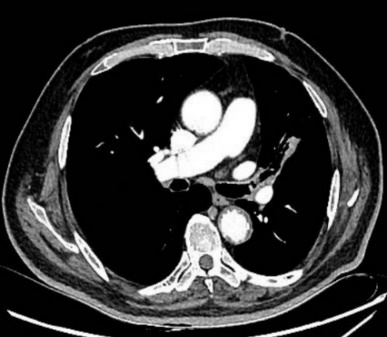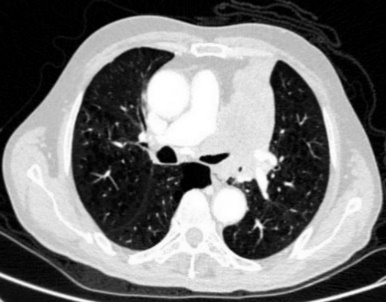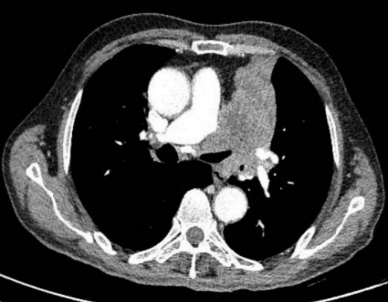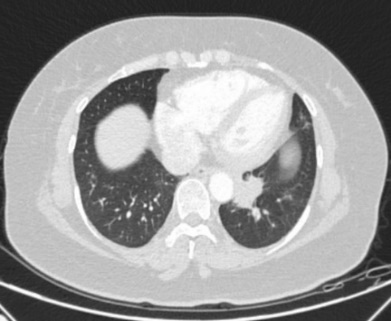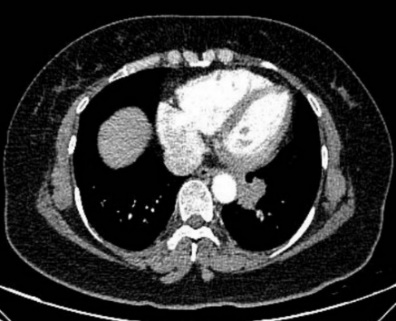 |
| Surgery | Yes |
| Reasons for unresectability after neoadjuvant treatment | - |

| Patient Number | 022 |
| --- | --- |
| Treatment arm | A |
| PD-L1 expression | Unknown |
| Clinical stage | IIIA (cT2aN2M0) |
| An enhanced computed tomography scan of the chest at baseline | 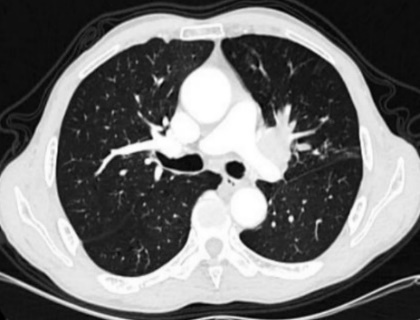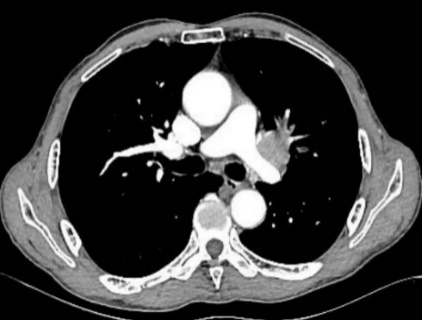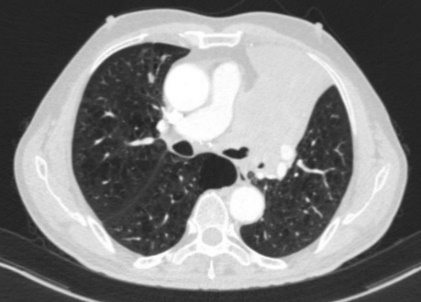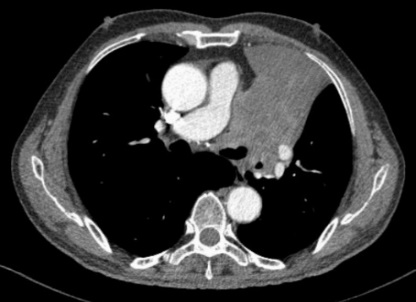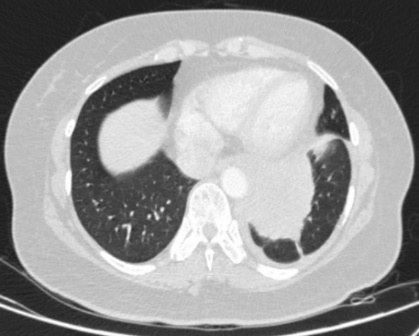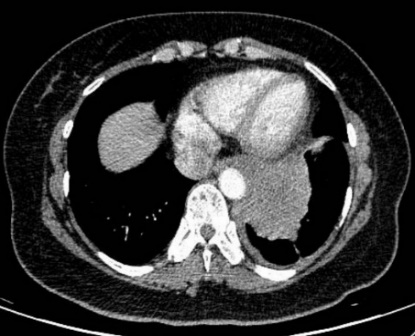 |
| Reasons for initial unresectability | Tumor invading the left pulmonary artery |
| Number of neoadjuvant treatment cycles | 3 |
| An enhanced computed tomography scan of the chest after neoadjuvant treatment | 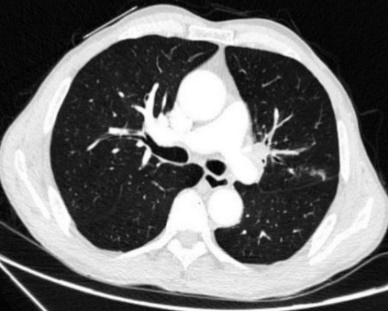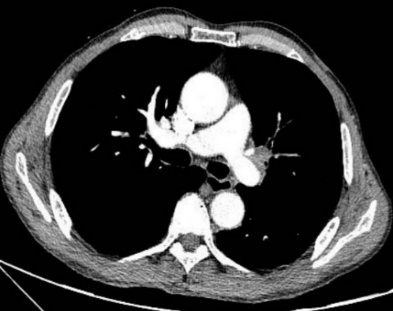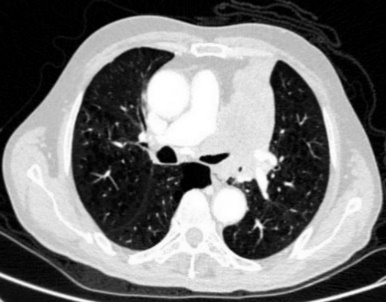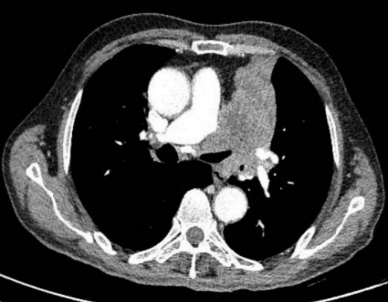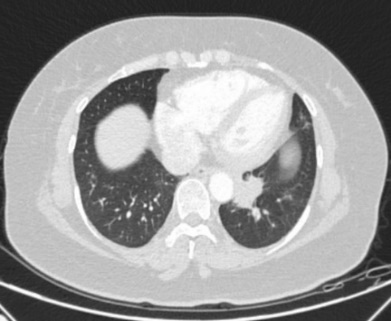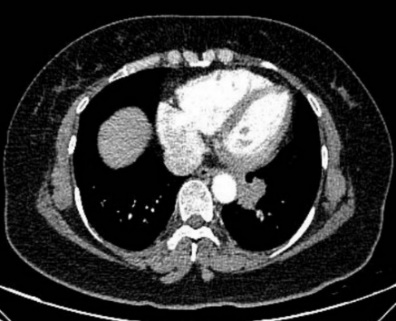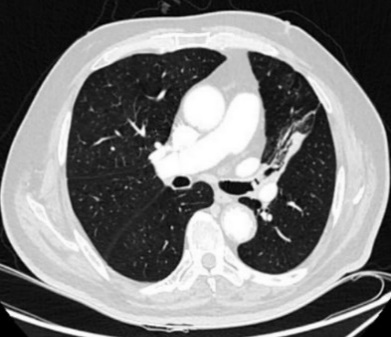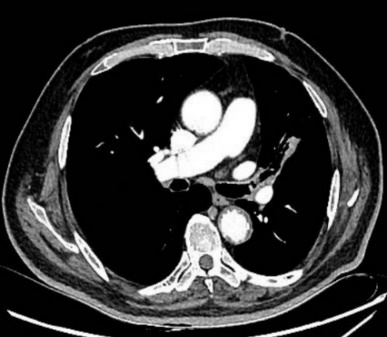 |
| Surgery | No |
| Reasons for unresectability after neoadjuvant treatment | Assessed as incomplete resectability: tumor still invading the left pulmonary artery. |

| Patient Number | 024 |
| --- | --- |
| Treatment arm | A |
| PD-L1 expression | < 1% |
| Clinical stage | IIIA (cT1cN2M0) |
| An enhanced computed tomography scan of the chest at baseline | 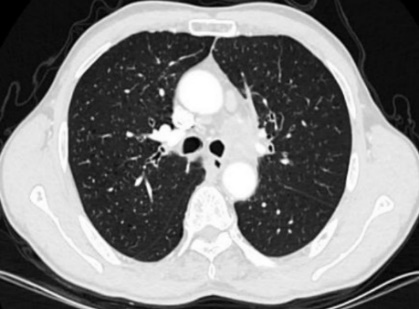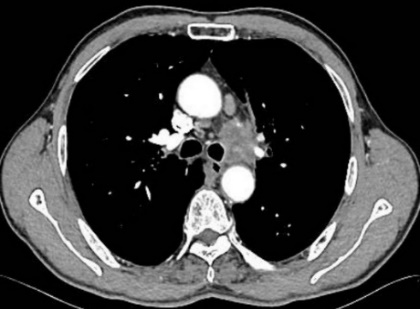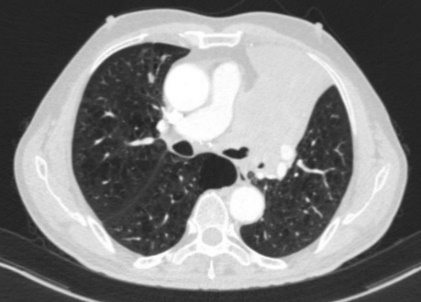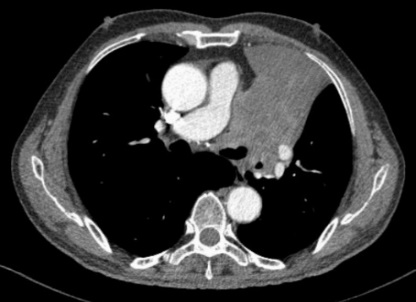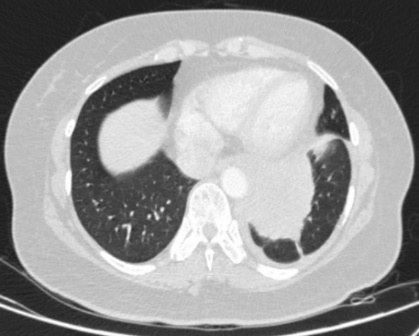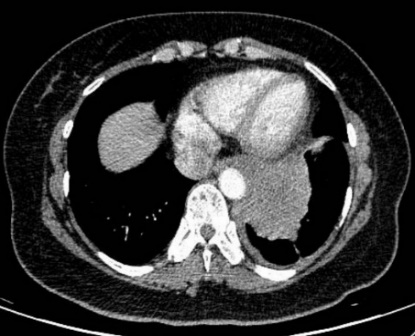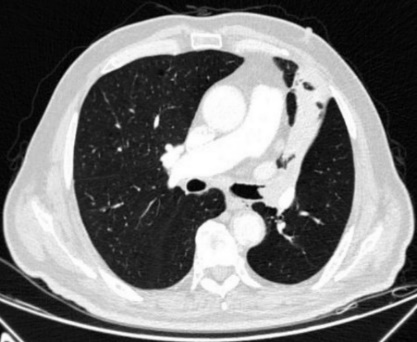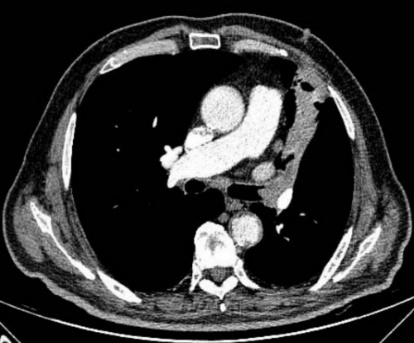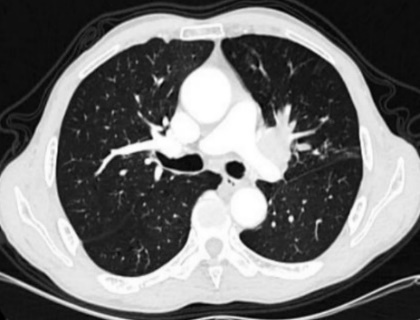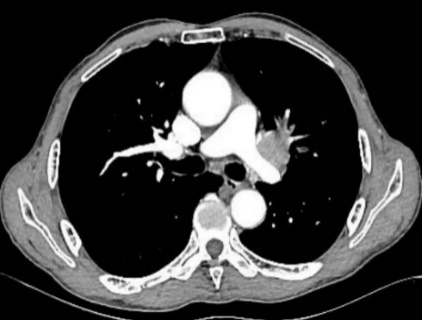 |
| Reasons for initial unresectability | Multistation metastasis in mediastinal lymph nodes with bulky structures. |
| Number of neoadjuvant treatment cycles | 2 |
| An enhanced computed tomography scan of the chest after neoadjuvant treatment | 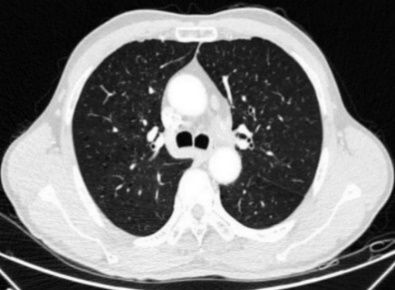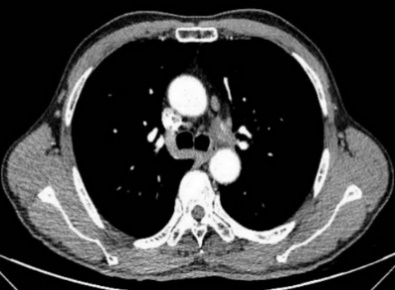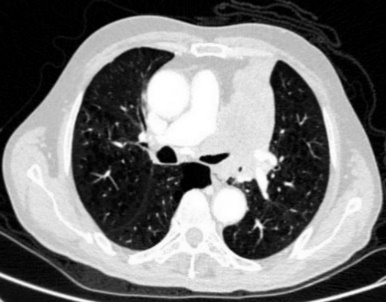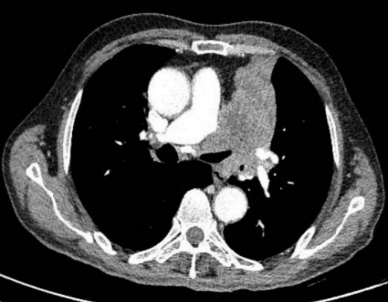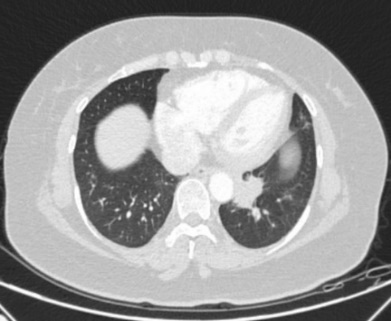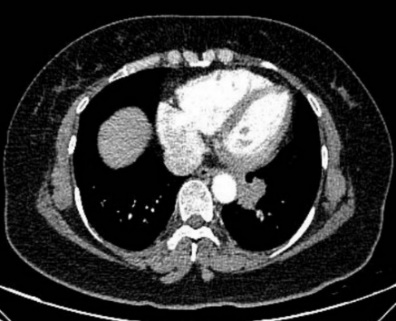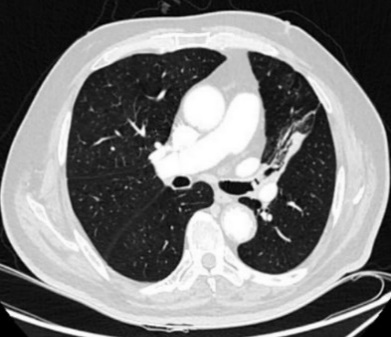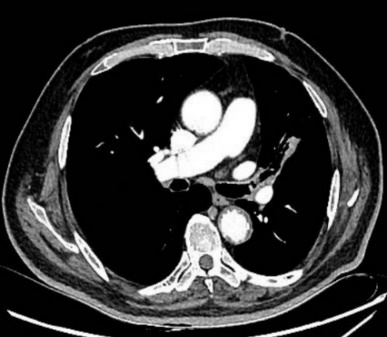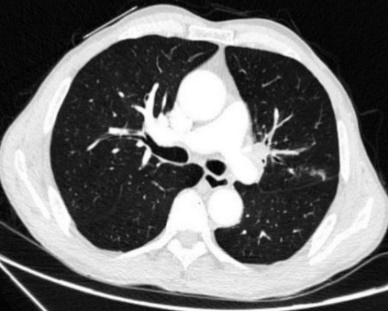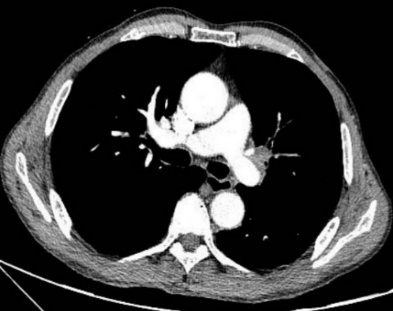 |
| Surgery | Yes |
| Reasons for unresectability after neoadjuvant treatment | - |

| Patient Number | 027 |
| --- | --- |
| Treatment arm | B |
| PD-L1 expression | 1%-49% |
| Clinical stage | IIIA (cT2bN2M0) |
| An enhanced computed tomography scan of the chest at baseline | 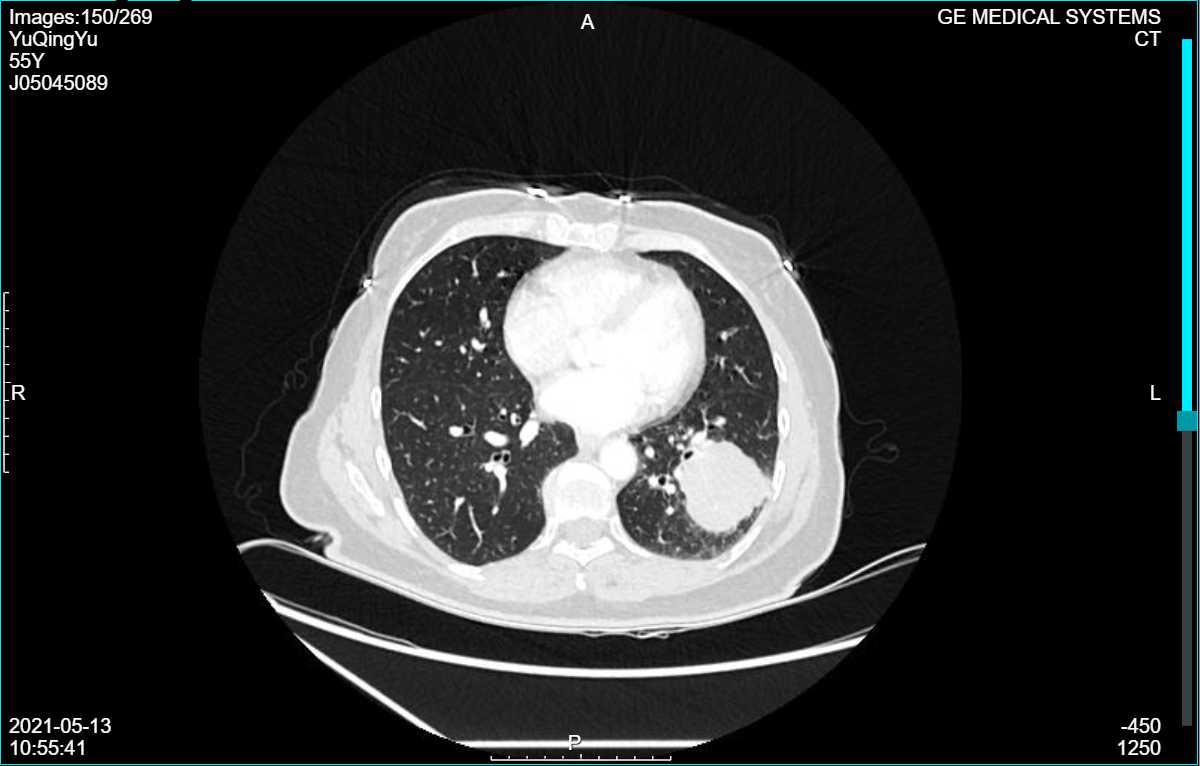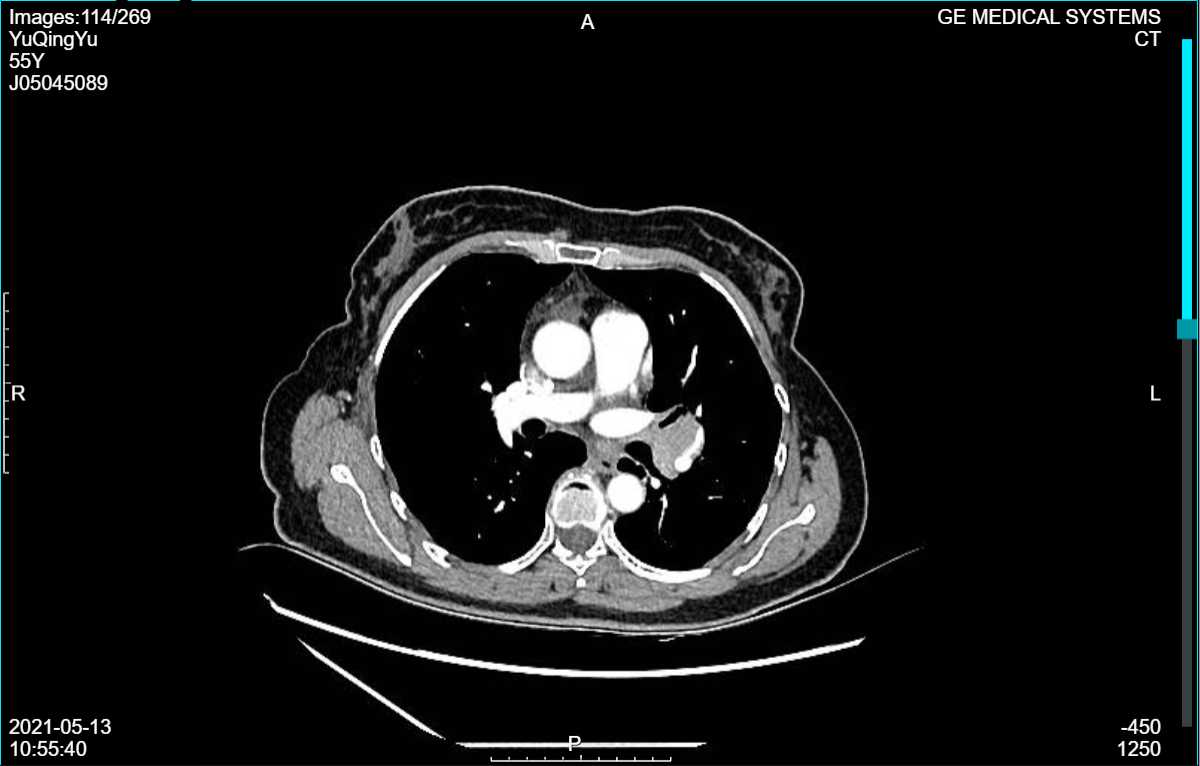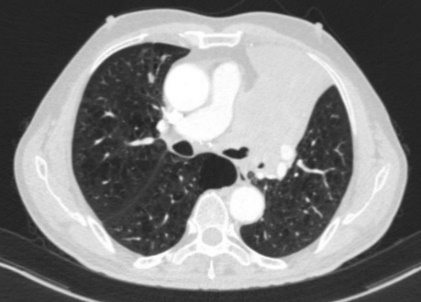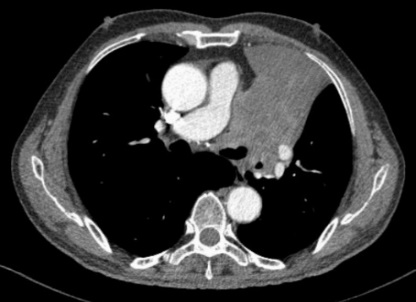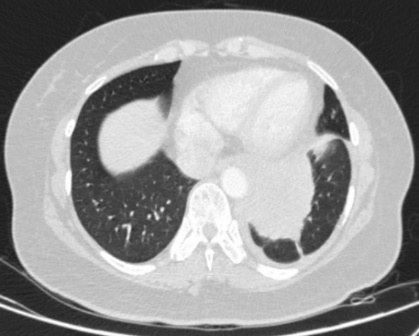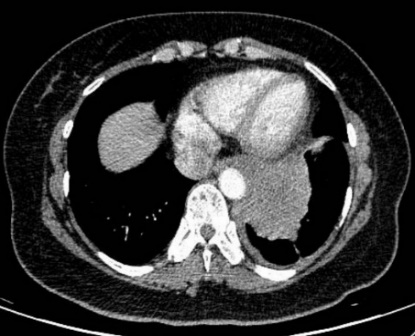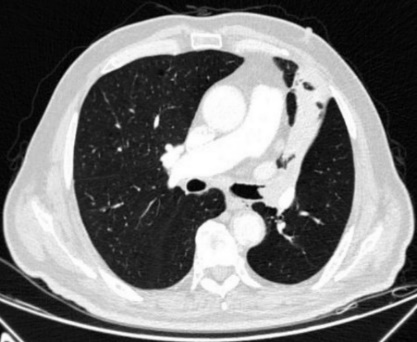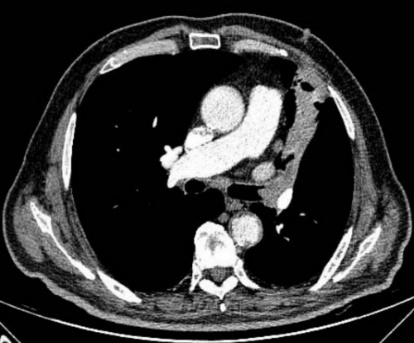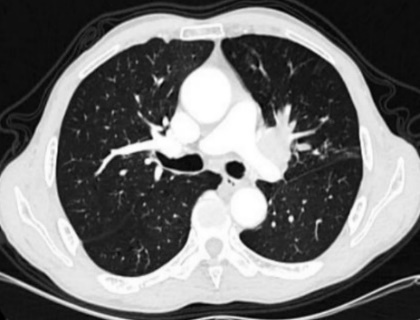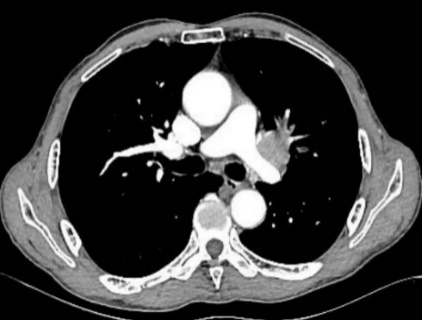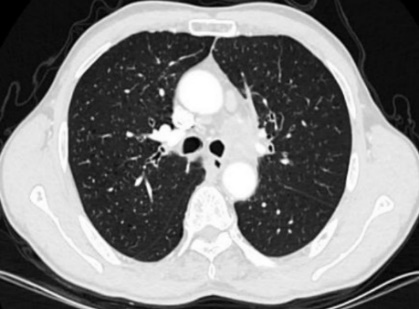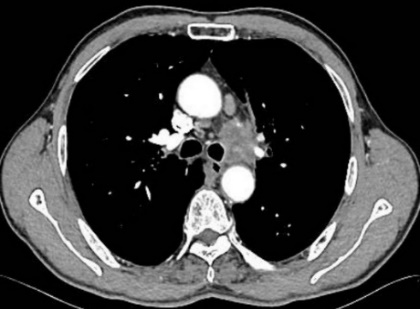 |
| Reasons for initial unresectability | The enlarged hilar lymph nodes invading left superior and inferior lobar bronchus, as well as the left superior pulmonary vein, with the possibility of failure to achieve R0 resection even with left pneumonectomy. |
| Number of neoadjuvant treatment cycles | 2 |
| An enhanced computed tomography scan of the chest after neoadjuvant treatment | 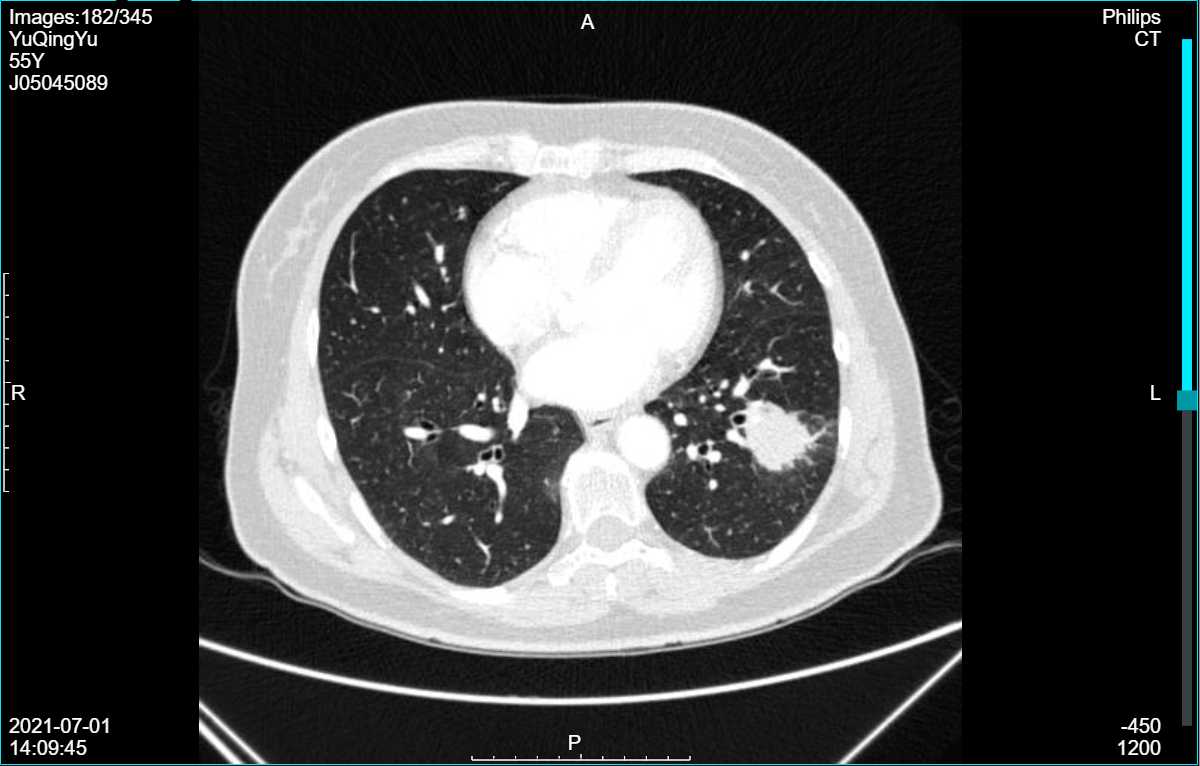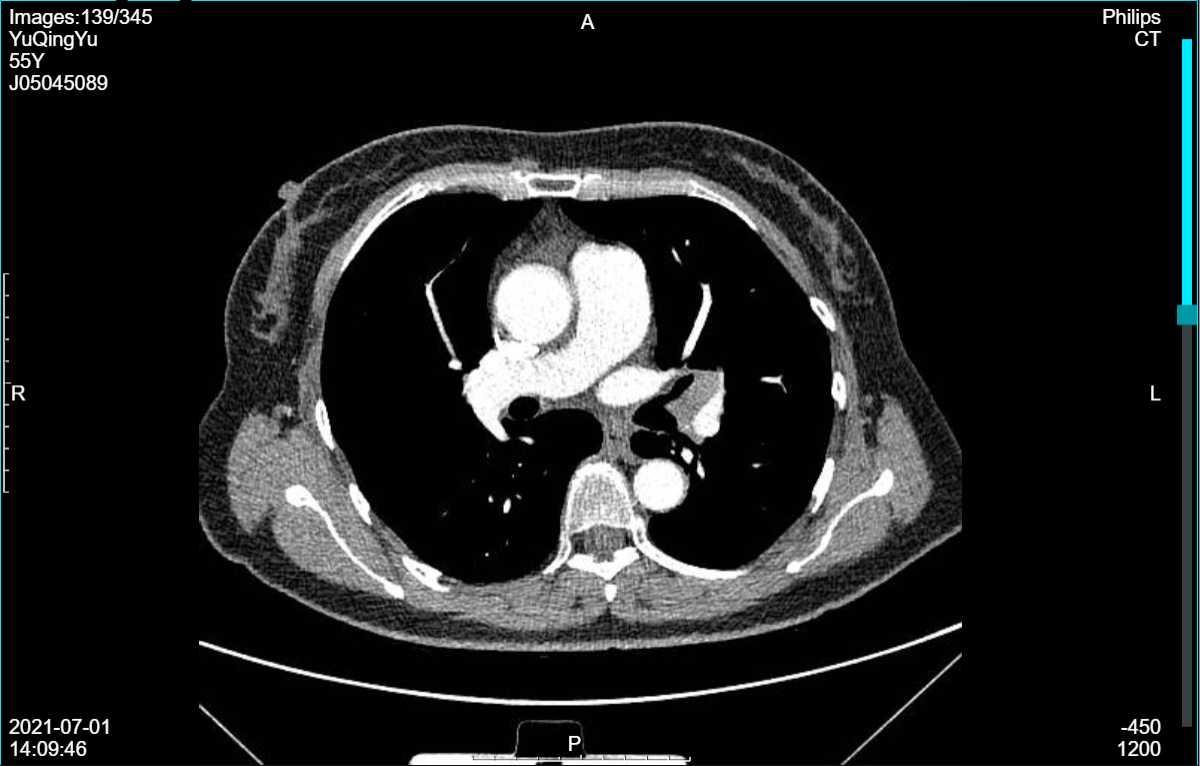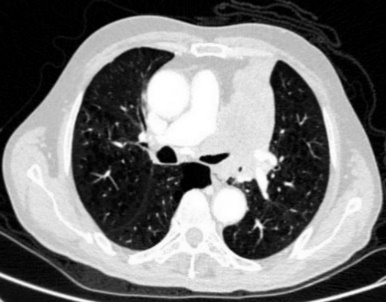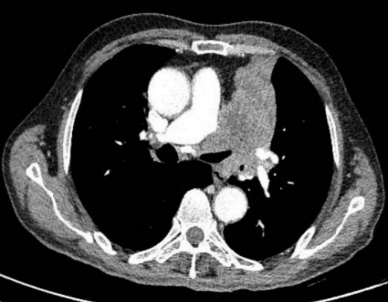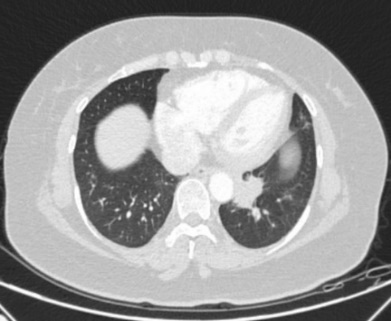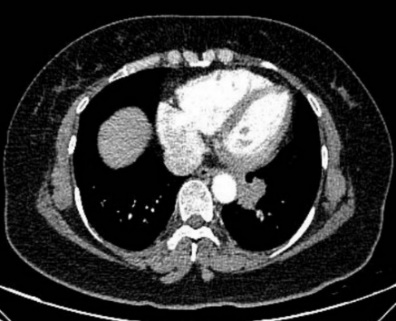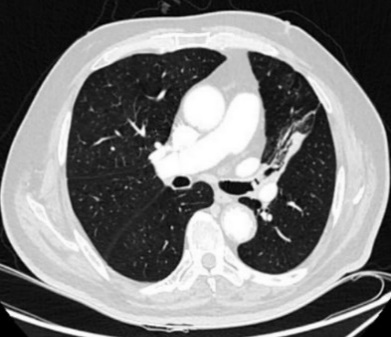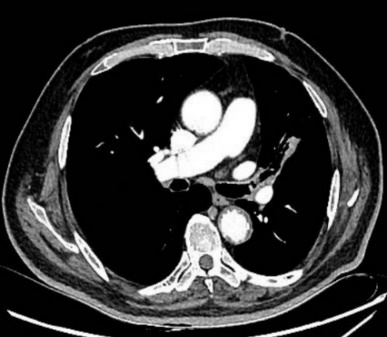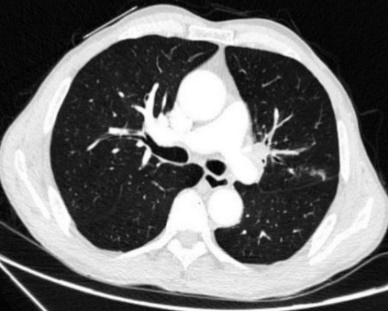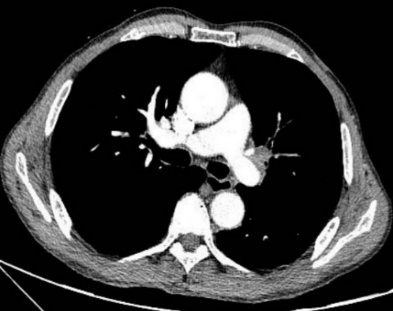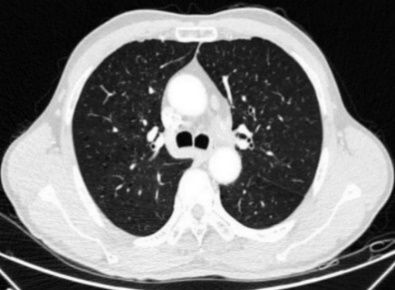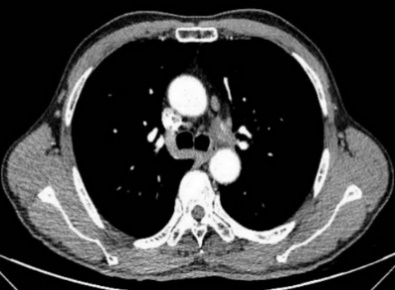 |
| Surgery | Yes |
| Reasons for unresectability after neoadjuvant treatment | - |

| Patient Number | 035 |
| --- | --- |
| Treatment arm | B |
| PD-L1 expression | ≥ 50% |
| Clinical stage | IIIA (cT4N0M0) |
| An enhanced computed tomography scan of the chest at baseline | 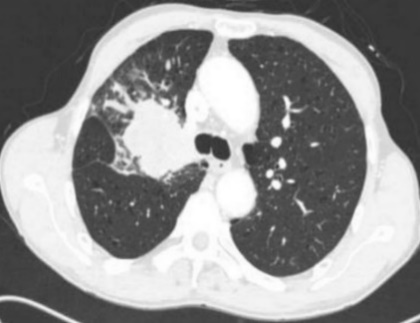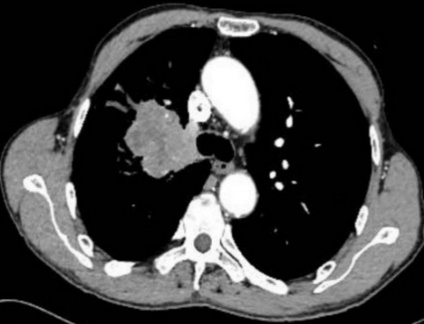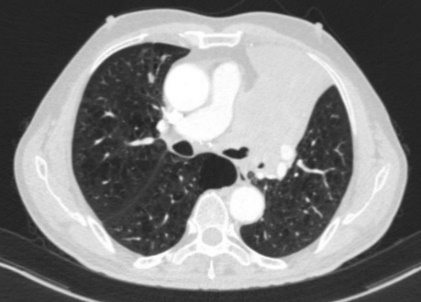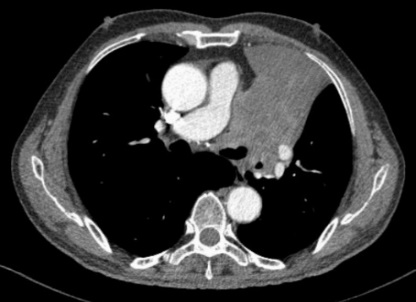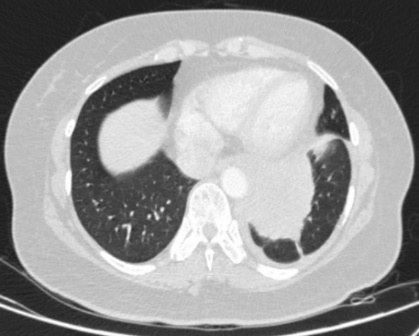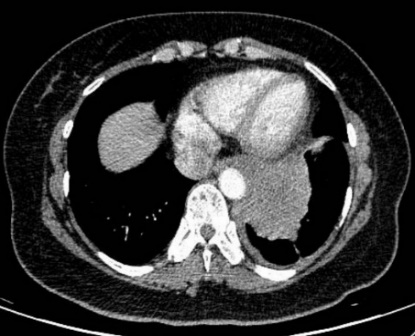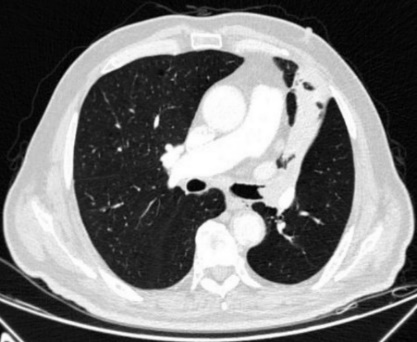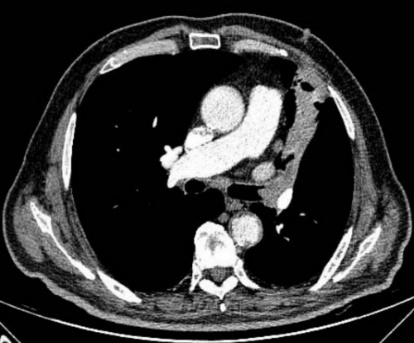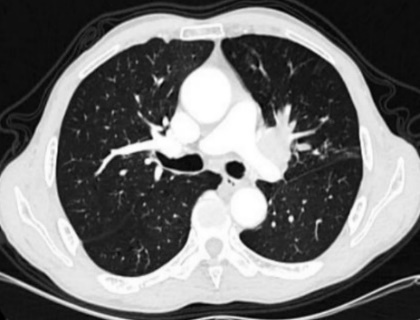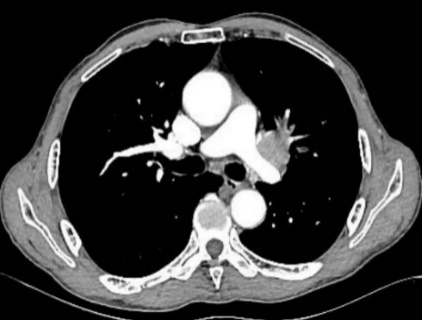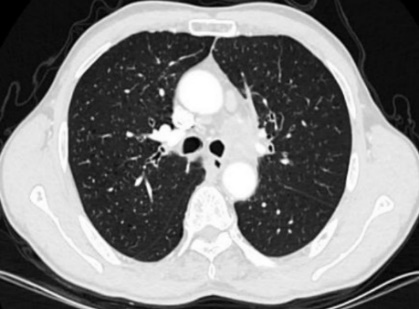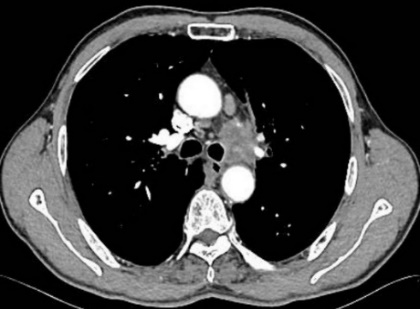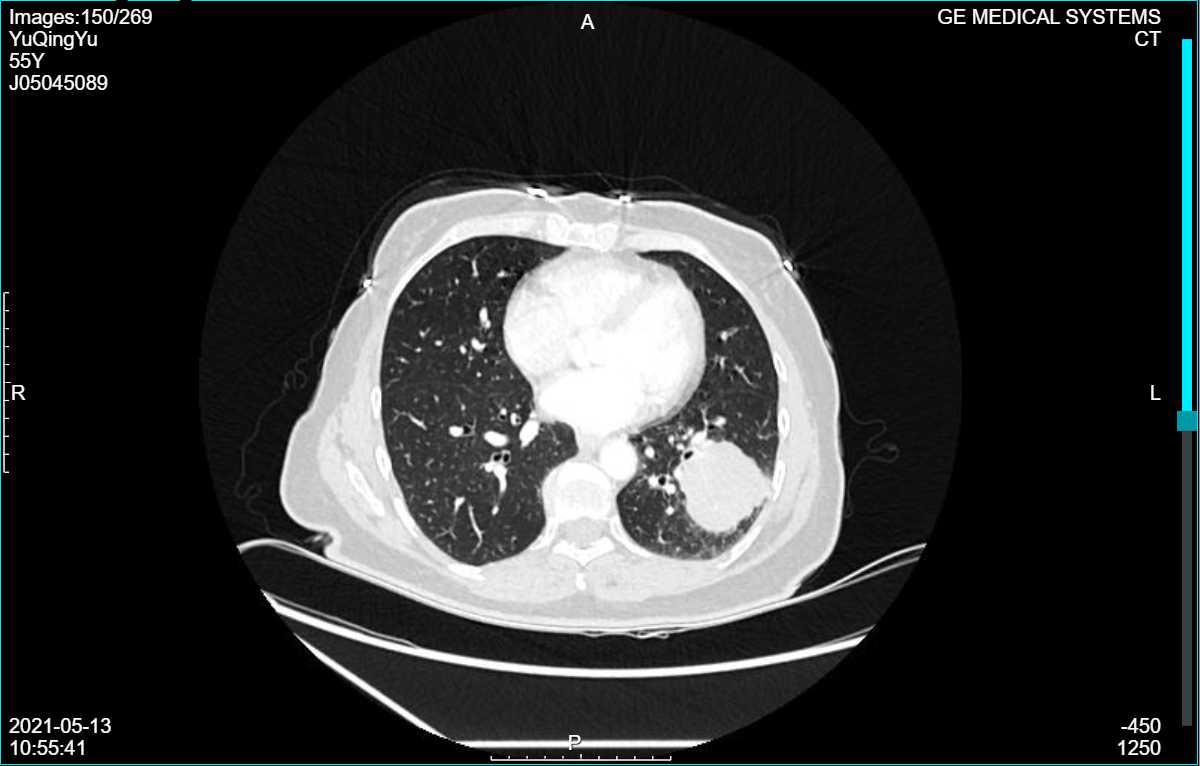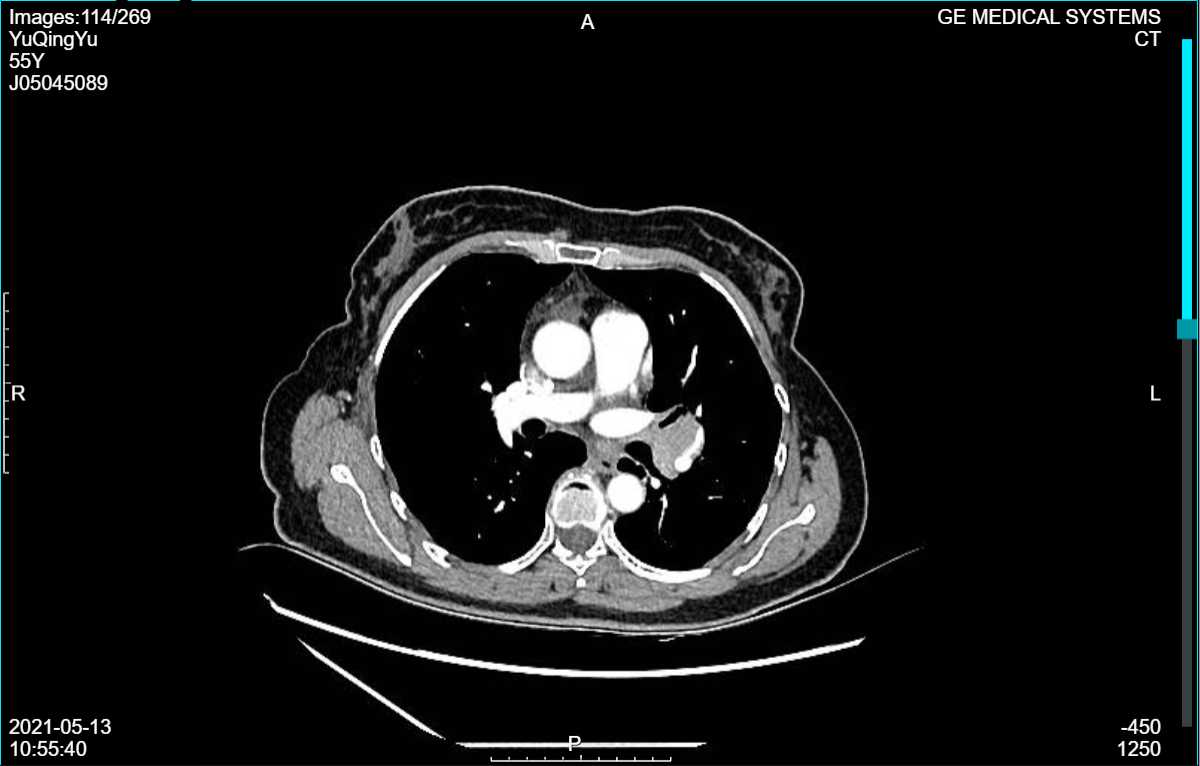 |
| Reasons for initial unresectability | Tumor invading the carina and the right primary bronchus. |
| Number of neoadjuvant treatment cycles | 3 |
| An enhanced computed tomography scan of the chest after neoadjuvant treatment | 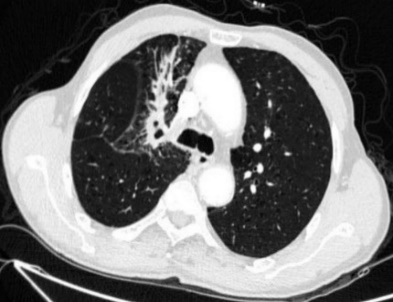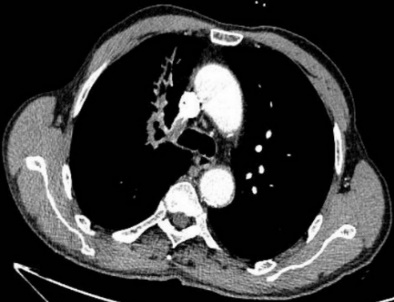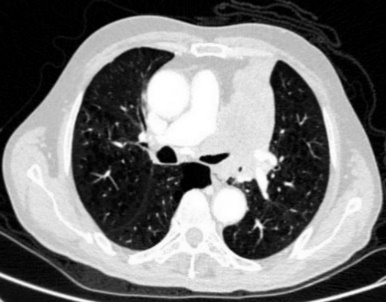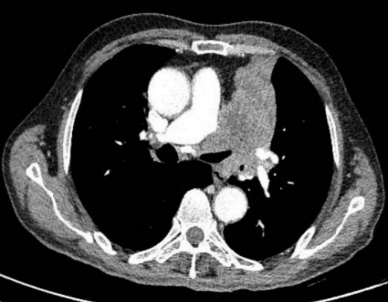 |
| Surgery | Yes |
| Reasons for unresectability after neoadjuvant treatment | - |

| Patient Number | 040 |
| --- | --- |
| Treatment arm | B |
| PD-L1 expression | ≥ 50% |
| Clinical stage | IIIA (cT3N1M0) |
| An enhanced computed tomography scan of the chest at baseline |  |
| Reasons for initial unresectability | Tumor invading the left pulmonary artery |
| Number of neoadjuvant treatment cycles | 4 |
| An enhanced computed tomography scan of the chest after neoadjuvant treatment |  |
| Surgery | No |
| Reasons for unresectability after neoadjuvant treatment | Assessed as incomplete resectability: tumor still invading the left pulmonary artery. |

| Patient Number | 044 |
| --- | --- |
| Treatment arm | B |
| PD-L1 expression | 1%-49% |
| Clinical stage | IIIA (cT2bN2M0) |
| An enhanced computed tomography scan of the chest at baseline |  |
| Reasons for initial unresectability | Tumor invading the carina and the superior vena cava |
| Number of neoadjuvant treatment cycles | 2 |
| An enhanced computed tomography scan of the chest after neoadjuvant treatment |  |
| Surgery | No |
| Reasons for unresectability after neoadjuvant treatment | Assessed as incomplete resectability: tumor still invading the carina. |

| Patient Number | 045 |
| --- | --- |
| Treatment arm | B |
| PD-L1 expression | ≥ 50% |
| Clinical stage | IIIA (cT2bN2M0) |
| An enhanced computed tomography scan of the chest at baseline |  |
| Reasons for initial unresectability | Multistation metastasis in mediastinal lymph nodes with bulky structures. |
| Number of neoadjuvant treatment cycles | 3 |
| An enhanced computed tomography scan of the chest after neoadjuvant treatment |  |
| Surgery | Yes |
| Reasons for unresectability after neoadjuvant treatment | - |
