# Supplementary material for: Neoadjuvant camrelizumab (an anti-PD-1 antibody) plus chemotherapy or apatinib (a VEGFR-2 inhibitor) for initially unresectable stage II–III non-small-cell lung cancer: a multicentre, two-arm, phase 2 exploratory study
Source: Signal Transduct Target Ther. 2024 Jun 14;9:145. doi: 10.1038/s41392-024-01861-w (PMC11176298; doi:10.1038/s41392-024-01861-w)
Supplement: Supplementary file 3 — Protocol [file 41392_2024_1861_MOESM3_ESM.pdf]

Neoadjuvant anti-PD-1 Drug Camrelizumab Combined with Apatinib or Chemotherapy for Resectable and Initially Unresectable Stage II-III NSCLC: A Multicenter, Phase II Clinical Trial

Program No.: MA-NSCLC-II-001

Principal Investigator: Prof. Gening Jiang, Prof. Peng Zhang

Leading Site: Shanghai Pulmonary Hospital

Study Period: September 2019 - May 2026

Version No.: 4.0

Version Date: July 16, 2020

Confidentiality statement

The information contained in this document, in particular the unpublished data, belongs to the sponsor of this study and is provided to you as confidential by virtue of treating you as an investigator, potential investigator, or consultant, and is available for review by you and your research team, as well as by independent ethics committees/institutional review boards. It must be made clear that this information may not be divulged to others without the written authorization of the sponsor, except for the purpose of obtaining informed consent from patients who may use the drug.

Version History/Revision History

Document	Version Date	Reason for revision and summary of changes
V1.0	July 19, 2019	Inapplicable
V2.0	August 7, 2019	● Revision of test groupings<br>● Subject population selection "Stage IB-IIIA (N2 single site)" revised to "Stage II-IIIA (N1)"<br>● Increase in Camrelizumab and Apatinib adverse effects
V3.0	March 18, 2020	● Revision of the purpose of the study<br>● Revision of emission standards<br>● Research visits were updated
V4.0	July 16, 2020	● Sample size revised<br>● Entry standards were revised<br>● Secondary endpoints were updated<br>● Research visits were updated<br>● Research Flowchart Update

Table of Contents

Protocol Synopsis **4**

Study Flow Chart **13**

List of Abbreviations **16**

1. Background and rationale for the study **18**

1.1 Epidemiology of lung cancer 18

1.2 Current status of neoadjuvant therapy for lung cancer 18

1.3 Immunotherapy of tumors 19

1.4 Advances in immuno-neoadjuvant therapy in lung cancer 20

1.5 Camrelizumab 22

1.6 Apatinib 23

2. Study Objectives **25**

3. Study Design **25**

3.1 Research design and program 25

3.2 Selection and Withdrawal of the Subject Population 25

3.2.1 Enrollment Criteria 25

3.2.2 Exclusion criteria 28

3.2.3 Subject exit criteria 31

3.2.4 Criteria for study termination 31

3.3 Research time 31

3.4 Treatment plan 31

3.4.1 Treatment arrangements 32

3.4.2 Neoadjuvant drug monitoring and management 33

3.4.3 Combined treatment and smoking 44

3.4.4 Concomitant medication 44

3.4.5 Treatment compliance 45

3.5 Research indicators 45

3.6 Research process 46

3.6.1 Screening phase 46

3.6.2 Preoperative treatment period 48

3.6.3 Preoperative assessment: 49

3.6.4 Surgical period 50

3.6.5 Postoperative assessment 50

3.6.6 Postoperative adjuvant therapy period (observational study)......................51
3.6.7 Postoperative follow-up period 51
3.6.8 Unplanned visits 52
3.6.9 Follow-up of subjects with disease recurrence or who have not relapsed at the end of the study (after 5 years postoperatively)...52
3.7 Quality of data 53
3.8 Archives 53
4. Ethical and legal aspects 53
4.1 Independent Ethics Committee (IEC) or Institutional Review Board (IRB) 53
4.2 Ethical guidance for this study 53
4.3 Information for Subjects and Informed Consent...54
4.4 Confidentiality..54
4.5 Maintenance of study documents, medical record report forms and records.....55
4.5.1 Researcher folder/file saving 55
4.5.2 Audits and inspections...55
4.5.3 Electronic medical record report form..55
5. Monitoring of research 56
6. Statistical analysis plan..56
6.1 Sample size calculation 56
6.2 Analysis set 56
6.3 Summary of study data...57
6.4 Efficacy analysis 57
6.4.1 Main efficacy analysis 57
6.4.2 Secondary efficacy analysis...57
6.5 Safety analysis..58
7. Adverse event reporting 59
7.1 Adverse Event (AE) 59
7.1.1 Definition of AE 59
7.1.2. Criteria for determining the severity of AEs 60
7.1.3 Determination of the relationship between AE and the trial drug 60
7.2 Serious Adverse Event (SAE) 61
7.2.1 Definition of SAE...61
7.2.2 Hospitalization...61
7.2.3 Disease progression and death...62

7.2.4 Potential drug-related liver damage 63
7.2.5 Perform other anti-tumor treatments 64
7.3 AE/SAE reporting 64
7.3.1 AE/SAE collection and follow-Up 64
7.3.2 SAE reporting procedures 65
7.4 Pregnancy Reporting 65
Reference 67

Protocol Synopsis

**Study Title**	Neoadjuvant anti-PD-1 Drug Camrelizumab Combined with Apatinib or Chemotherapy for Resectable and Initially Unresectable Stage II-III NSCLC: A Multicenter, Phase II Clinical Trial
**Program No.**	MA-NSCLC-II-001
**Version No.**	V4.0
**Organization in Charge**	Shanghai Pulmonary Hospital
**Principal Investigator**	Gening Jiang, Peng Zhang
**Center for Research**	6
**Study Objectives**	**Primary objective**<br>To evaluate the efficacy and safety of neoadjuvant therapy of the anti-PD-1 agent Camrelizumab in combination with Apatinib or chemotherapy for resectable and initially unresectable stage II-III NSCLC.
	**Exploratory objectives**<br>Explore potentially sensitive markers for immunotherapy, including but not limited to tumor-infiltrating lymphocytes and PD-L1 expression.
**Study Endpoints**	**Primary endpoint:**<br>The primary endpoint of this study was the major pathologic response (MPR) rate, which was the ratio of subjects experiencing major pathologic response to the total number of subjects undergoing surgery. Major pathologic response was defined as no more than 10% of residual viable tumor cells in the tumor bed in the postoperative specimen.<br>**Secondary endpoints:**<br>Secondary study endpoints included pathological complete response (pCR) rate, objective response rate (ORR), event-free survival (EFS), 1-year EFS rate, 2-year EFS rate, disease-free survival (DFS), 1-year DFS rate, 2-year DFS rate, overall survival (OS), quality of life (QoL), R0 resection rate, and safety.<br>**Other:** search for potentially sensitive markers for immunotherapy.
**Study Subjects**	Histopathologically confirmed EGFR/ALK wild-type, resectable or initially unresectable stage II-III NSCLC (as judged by the International Association for the Study of Lung Cancer [IASLC] Staging Manual in Thoracic Tumors, 8th edition).

**Study Design**	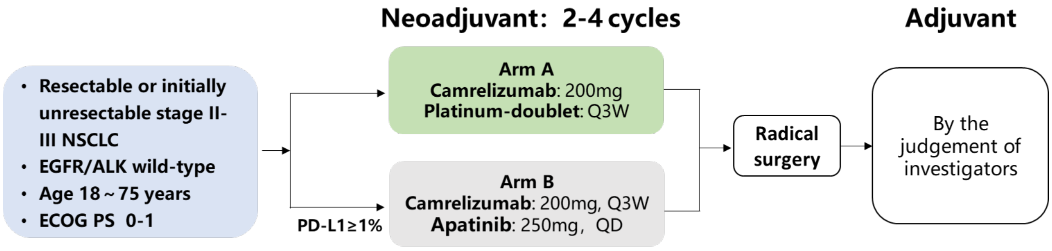
**Study Drug**	Camrelizumab for Injection<br>(Manufacturer: Suzhou Suncadia Biopharmaceuticals Co., Ltd.)<br>Apatinib Mesylate Tablets (abbreviated as "Apatinib")<br>(Manufacturer: Jiangsu Hengrui Medicine Co., Ltd.)
**Dosing Regimen**	The study was divided into two treatment arms with separate dosing regimens:<br>**Arm A: Camrelizumab + Platinum-doublet chemotherapy**<br>Camrelizumab: a fixed dose of 200 mg, intravenous drip over a period of 30-60 min, d1;<br>Chemotherapy regimen: ① Squamous carcinoma: Carboplatin AUC5 d1 + Gemcitabine 1000mg/m^2 , d1, d8 (or Paclitaxel 135~175 mg/m^2 , d1; or Docetaxel 60~75 mg/m^2 , d1; or Albumin Paclitaxel 260 mg/m^2, d1) ② Non-squamous carcinoma: Carboplatin AUC5 d1, Pemetrexed 500mg/m^2, d1), 3 weeks as a treatment cycle, and 2-4 cycles of preoperative administration.<br>**Arm B: Camrelizumab + Apatinib**<br>Camrelizumab: a fixed dose of 200 mg, intravenous drip over a period of 30-60 min, d1;<br>Apatinib: 250 mg/day orally once daily for 3 weeks as a treatment cycle, 2-4 cycles of preoperative administration.
**Inclusion Criteria**	**Arm A: Camrelizumab + Platinum-doublet chemotherapy**<br>(1) Age ≥ 18 years and ≤ 75 years;<br>(2) ECOG PS scores of 0-1;<br>(3) Treatment-naïve patients with histologically or cytologically confirmed stage II-III NSCLC (according to the IASLC Staging Manual in Thoracic Oncology, 8th Edition);<br>(4) At least one measurable lesion (according to RECIST 1.1 criteria);<br>(5) Life expectancy of at least 12 weeks.<br>(6) Other major organs (liver, kidneys, blood system, etc.) are normal.<br>• Hemoglobin ≥ 90 g/L (no blood transfusion, no use of hematopoietic factors and no drug correction within 2 weeks prior to first dose);<br>• Absolute neutrophil count (ANC) ≥ 1.5 × 10^9 /L;<br>• Platelet count ≥ 100 × 10^9 /L;<br>• Total bilirubin ≤ 1.5 times the upper limit of normal;<br>• ≤ 2.5 times the upper limit of normal values for alanine aminotransferase, glutamine

aminotransferase, and alkaline phosphatase;

• Serum creatinine ≤ 1.5 times the upper limit of normal; and endogenous creatinine clearance ≥ 60 ml/min;

• International normalized ratio (INR) of prothrombinogen time ≤ 1.5 and activated partial thromboplastin time (APTT) ≤ 1.5 times the upper limit of normal in patients who have not received anticoagulation therapy;

(7) No systemic metastases (including M1a, M1b, M1c);

(8) Completely resectable or initially unresectable disease. Initially unresectable disease is evaluated by a multidisciplinary clinical team (MDT) and is defined as meeting one of the following three conditions: ① Tumor invading vital structures, such as large blood vessels, the trachea or primary bronchus, but curative resection after tumor downgrading by neoadjuvant therapy was possible as judged by a preoperative assessment; ② Clinically confirmed lymph nodes with multistation metastasis or bulky fusion, and patients could tolerate hilar and mediastinal lymph node dissection after tumor downgrading by neoadjuvant therapy as determined by a preoperative evaluation; ③ According to preoperative evaluation, even if pneumonectomy was performed, especially right pneumonectomy, R0 resection may not be achieved;

(9) Adequate lung function must be available for the intended pulmonary resection procedure;

(10) Female subjects of childbearing potential must have a negative pregnancy test (serum or urine) performed within 72 hours prior to initiation of study drug administration and be willing to use a medically approved effective method of contraception (e.g., IUD, birth control pills, or condoms) for the duration of the study and for 90 days after final study drug administration; for male subjects whose partner is a female of childbearing potential, agree to use an effective method of contraception or have been surgically sterilized for the duration of the study and for 90 days after last drug administration.

(11) Subjects voluntarily enrolled in this clinical study and signed an informed consent form.

Arm B: Camrelizumab + Apatinib

(1) Age ≥ 18 years and ≤ 75 years;

(2) ECOG PS scores of 0-1;

(3) Treatment-naïve patients with histologically or cytologically confirmed stage II-III NSCLC (according to the IASLC Staging Manual in Thoracic Oncology, 8th Edition);

(4) Sufficient tumor tissue is available to detect PD-L1 expression and PD-L1 is ≥1%;

(5) At least one measurable lesion (according to RECIST 1.1 criteria);

(6) Life expectancy of at least 12 weeks.

(7) Other major organs (liver, kidneys, blood system, etc.) are normal.

• Hemoglobin ≥ 90 g/L (no blood transfusion, no use of hematopoietic factors and no drug correction within 2 weeks prior to first dose);

	• Absolute neutrophil count (ANC) ≥ 1.5×10^9 /L;<br>• Platelet count ≥ 100×10^9 /L;<br>• Total bilirubin ≤ 1.5 times the upper limit of normal;<br>• ≤ 2.5 times the upper limit of normal values for alanine aminotransferase, glutamine aminotransferase, and alkaline phosphatase;<br>• Serum creatinine ≤ 1.5 times the upper limit of normal; and endogenous creatinine clearance ≥ 60 ml/min;<br>• International normalized ratio (INR) of prothrombinogen time ≤ 1.5 and activated partial thromboplastin time (APTT) ≤ 1.5 times the upper limit of normal in patients who have not received anticoagulation therapy;<br>(8) No systemic metastases (including M1a, M1b, M1c);<br>(9) Completely resectable or initially unresectable disease. Initially unresectable disease is evaluated by a multidisciplinary clinical team (MDT) and is defined as meeting one of the following three conditions: ① Tumor invading vital structures, such as large blood vessels, the trachea or primary bronchus, but curative resection after tumor downgrading by neoadjuvant therapy was possible as judged by a preoperative assessment; ② Clinically confirmed lymph nodes with multistation metastasis or bulky fusion, and patients could tolerate hilar and mediastinal lymph node dissection after tumor downgrading by neoadjuvant therapy as determined by a preoperative evaluation; ③ According to preoperative evaluation, even if pneumonectomy was performed, especially right pneumonectomy, R0 resection may not be achieved;<br>(10) Adequate lung function must be available for the intended pulmonary resection procedure;<br>(11) Female subjects of childbearing potential must have a negative pregnancy test (serum or urine) performed within 72 hours prior to initiation of study drug administration and be willing to use a medically approved effective method of contraception (e.g., IUD, birth control pills, or condoms) for the duration of the study and for 90 days after final study drug administration; for male subjects whose partner is a female of childbearing potential, agree to use an effective method of contraception or have been surgically sterilized for the duration of the study and for 90 days after last drug administration.<br>(12) Subjects voluntarily enrolled in this clinical study and signed an informed consent form.
**Exclusion criteria**	**Arm A: Camrelizumab + Platinum-doublet chemotherapy**<br>(1) Any systemic anticancer treatment for NSCLC, including surgical treatment, local radiotherapy, cytotoxic drug therapy, targeted therapy, immunotherapy, and traditional Chinese medicine antitumor therapy (excluding treatment for malignant tumors that have been previously eradicated and have been free of recurrence and metastasis for ≥5 years);<br>(2) Non-squamous cancer patients with EGFR activating mutations or ALK fusion mutations;<br>(3) Patients with other malignancies within five years prior to the start of this trial, excluding

cured carcinoma in situ of the cervix, basal cell carcinoma of the skin, or squamous cell carcinoma of the skin;

(4) Patients with any active autoimmune disease or history of autoimmune disease (e.g., uveitis, enteritis, hepatitis, pituitary gland inflammation, vasculitis, myocarditis, nephritis, hyperthyroidism, hypothyroidism (may be included after hormone replacement therapy), tuberculosis); patients with asthma in childhood that does not require any intervention in adulthood, and patients with a skin disorder that has been in complete response that does not require systemic therapy (e.g., vitiligo, psoriasis, or alopecia areata) may be included; patients requiring medical intervention with bronchodilators may not be included;

(5) Exclude those with the evidence of previous or current pulmonary fibrosis, interstitial pneumonitis, pneumoconiosis, radiation pneumonitis, drug-induced pneumonitis, imaging-confirmed active pneumonitis, and severely impaired lung function;

(6) Subjects who have been systemically treated with corticosteroids (>10 mg/day prednisone or other equivalent hormone) or other immunosuppressive agents within 2 weeks prior to the first dose of study drug. Inhaled or topical corticosteroids and adrenal hormone replacement therapy at doses ≤ 10 mg/day prednisone efficacy dose are permitted in the absence of active autoimmune disease;

(7) Allergy to the test drug;

(8) Comorbid HIV infection or active viral hepatitis;

(9) Pregnant or lactating women; subjects of childbearing potential who are unwilling or unable to use effective contraception;

(10) Those who suffer from neurological or mental illnesses who cannot cooperate;

(11) Other circumstances judged by the investigator to be inappropriate for enrollment.

Arm B: Camrelizumab + Apatinib

(1) Any systemic anticancer treatment for NSCLC, including surgical treatment, local radiotherapy, cytotoxic drug therapy, targeted therapy, immunotherapy, and traditional Chinese medicine antitumor therapy (excluding treatment for malignant tumors that have been previously eradicated and have been free of recurrence and metastasis for ≥5 years);

(2) Non-squamous cancer patients with EGFR activating mutations or ALK fusion mutations;

(3) Patients with other malignancies within five years prior to the start of this trial, excluding cured carcinoma in situ of the cervix, basal cell carcinoma of the skin, or squamous cell carcinoma of the skin;

(4) Patients with any active autoimmune disease or history of autoimmune disease (e.g., uveitis, enteritis, hepatitis, pituitary gland inflammation, vasculitis, myocarditis, nephritis, hyperthyroidism, hypothyroidism (may be included after hormone replacement therapy), tuberculosis); patients with asthma in childhood that does not require any intervention in adulthood, and patients with a skin disorder that has been in complete response that does not require systemic therapy (e.g., vitiligo, psoriasis, or alopecia areata) may be included; patients

requiring medical intervention with bronchodilators may not be included;
(5) Exclude those with the evidence of previous or current pulmonary fibrosis, interstitial pneumonitis, pneumoconiosis, radiation pneumonitis, drug-induced pneumonitis, imaging-confirmed active pneumonitis, and severely impaired lung function;
(6) Subjects who have been systemically treated with corticosteroids (>10 mg/day prednisone or other equivalent hormone) or other immunosuppressive agents within 2 weeks prior to the first dose of study drug. Inhaled or topical corticosteroids and adrenal hormone replacement therapy at doses ≤ 10 mg/day prednisone efficacy dose are permitted in the absence of active autoimmune disease;
(7) Imaging (CT or MRI) shows that the tumor invades large blood vessels or has an indistinct border with blood vessels;
(8) Those who had an arterial/venous thrombotic event such as cerebrovascular accident (including temporary ischemic attack, cerebral hemorrhage, and cerebral infarction), deep vein thrombosis, and pulmonary embolism in the 6 months prior to enrollment;
(9) Have experienced clinically significant bleeding symptoms or have a definite bleeding tendency, such as gastrointestinal bleeding, bleeding gastric ulcer, etc., within 3 months prior to enrollment, or are receiving thrombolytic or anticoagulant therapy;
(10) Significant hemoptysis symptoms or daily hemoptysis of 2.5mL or more within 1 month prior to enrollment;
(11) Hypertension that is not well controlled by antihypertensive medication (systolic blood pressure ≥140 mmHg or diastolic blood pressure ≥90 mmHg); or myocardial ischemia or myocardial infarction of grade II or above, or poorly controlled cardiac arrhythmia (including QTc interval ≥450ms for men and ≥470ms for women); or cardiac insufficiency of grade III-IV according to the NYHA criteria; or left ventricular ejection fraction (LVEF) of <50% according to Doppler echocardiography;
(12) Those who have had major surgery on other systems or severe trauma within 2 months prior to the start of this trial;
(13) Urine routine suggesting urinary protein ≥ (++), or 24h urine protein volume ≥ 1g or severe hepatic or renal insufficiency;
(14) Uncontrollable pleural effusion, pericardial effusion, or ascites requiring repeated drainage;
(15) Allergy to the test drug;
(16) Comorbid HIV infection or active viral hepatitis;
(17) Pregnant or lactating women; subjects of childbearing potential who are unwilling or unable to use effective contraception;
(18) Persons with neurological or mental illnesses who are unable to cooperate;
(19) Other circumstances that the investigator considers inappropriate for enrollment.

**Withdrawal Criteria**	Subjects who experience one or more of the following will withdraw from treatment:<br>(1) The subject requests to withdraw from treatment or withdraws the informed consent;<br>(2) Disease progression confirmed by imaging;<br>(3) Poor subject compliance;<br>(4) The subject is unable to tolerate the toxicity;<br>(5) Loss to follow-up, death, or pregnancy event;<br>(6) Other circumstances that, in the opinion of the investigator, necessitate termination of treatment.
**End of Treatment**	(1) Identified unintended, significant, or unacceptable risks to the subject.<br>(2) Research drug therapy is confirmed to be ineffective.<br>(3) Pre-existing efficacy results support early termination of the study.<br>(4) Significant mistakes in protocol are identified during the implementation of study.<br>(5) Completion of studies is extremely difficult due to significant lags in subject enrollment or frequent protocol deviations.<br>(6) Incomplete or unmeasurable data.
**Efficacy Evaluation**	**1. Primary Efficacy Endpoint:**<br>Major pathologic response (MPR) rate: the ratio of subjects experiencing major pathologic response to the total number of subjects undergoing surgery. Major pathologic response was defined as no more than 10% of residual viable tumor cells in the tumor bed in the postoperative specimen.<br>**2. Secondary Efficacy Endpoints:**<br>Pathological complete response (pCR) rate: the ratio of subjects who experienced pathological complete response to those who underwent surgery. Complete pathological response was defined as no residual viable tumor cells in the lung and lymph nodes (assessed by pathological review).<br>Objective response rate (ORR): the rate of subjects whose tumors shrank by a certain amount and remained a certain period of time, including complete response (CR) and partial response (PR) cases. Objective tumor response was assessed using the Response Evaluation Criteria in Solid Tumor (RECIST 1.1 criteria). The efficacy assessment criteria were classified as CR, PR, stable disease (SD), and progressive disease (PD) according to the RECIST 1.1 criteria.<br>Disease-free survival (DFS): defined as the time from the end of surgery to disease recurrence or death (due to any cause), 1- and 2-year DFS rates were counted.<br>Event-free survival (EFS): defined as the time from the start of the first dose to the occurrence of any of the following events. Events include: disease progression leading to inoperable disease, disease progression after surgery, or death from any cause, whichever occurs earliest.

	Overall Survival (OS): defined as the time from enrollment to death of participant due to any cause. Subjects who are alive at the time of analysis will have the date of their last contact as the cut-off date.<br>R0 resection rate: defined as the proportion of patients with margin-negative resection.<br>Quality of life score: refer to the questionnaire QOL-LC-13 of Shanghai Pulmonary Hospital.<br>**3. Exploratory Endpoints:** seeking for potentially sensitive markers for immunotherapy, including but not limited to tumor-infiltrating lymphocytes and PD-L1 expression.
**Safety Evaluation**	Observe any adverse events, including abnormal clinical symptoms and vital signs, abnormalities in laboratory tests, occurring in all subjects from the time of signing the informed consent form up to 90 days after the last dose of Camrelizumab or 30 days after the last dose of Apatinib (whichever is longer), record the characteristics of the clinical manifestations, severity, time of onset, duration, management, and prognosis, and determine the correlation between them and the test drug. The safety of the study drug will be evaluated using the National Cancer Institute Common Toxicity Criteria (NCI-CTCAE) version 5.0 criteria.
**Statistical methods**	**1. Statistical analysis of the population:**<br>Full Analysis Set (FAS): it includes all enrolled subjects who have received at least one dose of study drug according to the intention-to-treat (ITT) principle.<br>Efficacy Evaluable Analysis Set (EAS): it includes all subjects who have received at least one dose of study drug after enrollment and have undergone pathological assessment after surgical treatment.<br>Safety Analysis Set (SAS): it includes all enrolled subjects who have received at least one dose of study drug. The SS will be used for all safety analyses.<br>**2. Baseline statistical analysis**<br>Baseline, demographic characteristics, baseline tumor characteristics, medical history, comorbid medications, vital signs, and termination of the trial will be summarized for all enrolled subjects. For continuous data, the mean, standard deviation, range of distribution and median will be calculated; absolute values, frequencies and percentages will also be calculated.<br>**3. Validity analysis**<br>The primary endpoint of this study is MPR, the number of cases and percentage of subjects under each classification will be summarized and 95% confidence intervals for the rates will be calculated using the Clopper-Pearson method.<br>Secondary efficacy analysis: for secondary efficacy endpoints in the dichotomous categories (including pCR, ORR, R0 resection rate), the number of cases and percentage of subjects under each category will be summarized and 95% confidence intervals for the rates will be calculated using the Clopper-Pearson method.<br>For efficacy endpoints of the time-to-event type (including EFS, DFS, OS), the number and percentage of subjects with events and censored will be summarized, and summary statistics will

	be provided for the type of event and the reason for censoring. The censoring rules for time-to-event endpoints are detailed in the statistical analysis plan.<br>The median survival time and survival rate will be estimated using the Kaplan-Meier method and survival curve will be plotted. The 95% confidence interval for survival time will be calculated using the Brookmeyer-Crowley method and the 95% confidence interval for survival rate will be calculated using the log(-log) method.<br>**4. Security Analysis**<br>Treatment-emergent adverse events (TEAE) for this study are defined as any adverse event that occurs or worsens after the first dose of study drug through 90 days after the last dose. All summary tables are based on TEAE; adverse events in screening-period will be provided as a list only.
**Study Schedule**	Estimated first subject enrollment: 2020.07<br>Estimated completion of enrollment: 2021.05<br>Estimated end of study: 2026.05

Study Flow Chart

Study steps	Screening period (A)		Pre-operative treatment period (2-4 cycles)				Preoperative evaluation	Surgery	Postoperative evaluation	Survival follow-up [23]	End of study
			Cycle 1	Cycle 2	Cycle 3	Cycle 4					
**Visit Cycle**	**-D28-D0**	**-D7-D0**	**C1D1**	**C2D1±3**	**C3D1±10**	**C4D1±10**	**3-4 weeks after last dose**	**Within 30d of preoperative evaluation**	**Postoperative 30d±10**		
Informed consent [1]	X										
Demographics/medical history/family history/allergy history/smoking history	X										
Tumor diagnosis	X										
Checking of entry criteria		X									
Vital signs		X		X	X	X	X		X		
Physical examination		X		X	X	X	X		X		
Genetic testing (EGFR and ALK) [2]	X										
PD-L1 expression assay [3]	X										
ECOG PS		X		X	X	X	X		X		
Blood routine [4]		X		X	X	X	X		X		
Blood biochemistry [5]		X		X	X	X	X		X		
Myocardial enzyme profiles [6]		X					X		X		
Electrocardiogram [7]	X			X	X	X	X		X		
Echocardiography [8]	X						X				
Coagulation [9]		X			X		X		X		
Pulmonary function [10]	X						X				
Urine routine [11]		X		X	X	X	X		X		
Stool routine [12]		X			X		X				
HIV testing	X										
Hepatitis indicators [13]	X						X				
Thyroid function [14]	X			X	X	X	X		X		
Pituitary-adrenal axis test [15]		X			X		X				

**Study steps**	**Screening period (A)**		**Pre-operative treatment period (2-4 cycles)**				**preoperative valuation**	**Surgery**	**Postoperative evaluation**	**Survival follow-up [23]**	**End of study**
			**Cycle 1**	**Cycle 2**	**Cycle 3**	**Cycle 4**					
**visiting period**	**-D28-D0**	**-D7-D0**	**C1D1**	**C2D1±3**	**C3D1±10**	**C4D1±10**	**3-4 weeks after last dose**	**Within 30d of preoperative evaluation**	**Postoperative 30d±10**		
Pregnancy test (women of childbearing age) [16]		**X**							**X**		
Venous blood 10 ml (for research) [17]	**X**			**X**	**X**	**X**	**X**				
Quality of life scores [18]	**X**			**X**	**X**	**X**	**X**		**X**		
Imaging [19]	Screening period, pre-medication of 3rd and 4th cycle, pre-operative evaluation, and post-operative evaluation										
Adverse events [20]	**X**		**X**	**X**	**X**	**X**	**X**	**X**	**X**		
Concomitant medications	**X**		**X**	**X**	**X**	**X**	**X**	**X**	**X**		
Treatment assessment page [21]											
Surgical records								**X**			
Pathologic assessment [22]								**X**			
Evaluation at the end of the follow-up visit											**X**

Note: The results of all laboratory and imaging tests that have been done before the subject signed the informed consent form are accepted as screening period data. In addition to the examination items and time points listed in the table, the investigator may add items that are necessary at any time, and the results of the examination will be filled in the eCRF "Unplanned Visits".

[1] Prior written informed consent must be obtained prior to the performance of any clinical research procedure, except for tumor imaging and tumor tissue biopsy, which are available within a specified time frame prior to the first dose.

[2] The study center must be able to provide documentation of the subject's EGFR and ALK mutation testing prior to the first dose. If the center is unable to provide this source documentation, the subject will need to be retested.

[3] All subjects should have as many puncture specimens as possible for PD-L1 expression testing (Dako 22C3 antibody) prior to enrollment, and subjects enrolled in Arm B must be guaranteed PD-L1 ≥1%.

[4] Routine blood counts including complete blood counts and classifications: hemoglobin, red blood cell count, white blood cell count, neutrophil count, lymphocyte count, and platelet count; Screening is performed within 7 days prior to the first dose, prior to the 2nd, 3rd, and 4th cycle of administration, at the preoperative evaluation, and at the postoperative evaluation visit .

[5] Blood biochemistry tests include : Lactate dehydrogenase (LDH), Alkaline phosphatase (ALP), Total cholesterol (TC), Triglycerides (TG), Total Protein (TP), Albumin (ALB), Fasting Glucose (GLU), Glutamine transaminase (ALT), Glutamine transaminase (AST), Gamma-glutamyltranspeptidase (GTP), Total bilirubin (TBIL), Direct Bilirubin (DBIL), creatinine (Cr), urea nitrogen (BUN), potassium (K+), sodium (Na+), and chloride (Cl-); Screening was performed within 7 days prior to the first dose, prior to Cycles 2, 3, and 4, at preoperative evaluation, and at the postoperative evaluation visit.

[6] Myocardial enzyme profiles including creatine kinase (CK), creatine kinase isoenzyme (CK-MB), myoglobin (Mb), and troponin (cTnI); performed within 7 days prior to the first dose of the medication, preoperative evaluation, and postoperative evaluation.

[7] 12-lead electrocardiograms, screening period, prior to administration of medications in cycles 2, 3 and 4, at preoperative evaluation and postoperative evaluation visits.

[8] Doppler echocardiography: performed during the screening period and at the preoperative evaluation visit.

[9] Coagulation: including PT, APTT, INR, TT, FDP; within 7 days prior to the first dose, prior to cycle 3, at the preoperative evaluation and postoperative evaluation visits.

[10] Pulmonary function: normal lung function to tolerate resection of lung cancer; or lung function sufficient to tolerate surgery as assessed by the investigator; screened at screening, preoperative evaluation visit.

[11] Urine routine: urine protein (PRO), urine glucose (GLU), urine occult blood (urine erythrocytes, urine leukocytes); examined within 7 days prior to the first dose, prior to the 2nd, 3rd, and 4th cycle of administration, at the time of the preoperative evaluation, and at the postoperative evaluation visit.
[12] Stool routine: fecal occult blood OB test, if fecal occult blood is positive it needs to be reviewed, review of fecal occult blood positivity needs to be gastroenteroscopy; within 7 days prior to the first dose of the drug, prior to the 3rd cycle of the drug and at the preoperative evaluation visit.
[13] Hepatitis indicators, including hepatitis B testing (hepatitis B surface antigen, hepatitis B surface antibody, hepatitis B e antigen, hepatitis B e antibody, hepatitis B core antibody, hepatitis B core antibody Igm assay) and HCV antibody screening; screening is performed during the screening period and at the preoperative evaluation visit.
[14] Thyroid function: free triiodothyronine (FT3), free thyroxine (FT4), thyroid-stimulating hormone (TSH), triiodothyronine (T3), and thyroxine (T4); screened during the screening period, prior to administration of medications for cycles 2, 3, and 4, at preoperative evaluation, and at postoperative evaluation visits.
[15] Pituitary-adrenal axis (HPA) tests: These include hypothalamic corticotropin-releasing hormone (CRH), pituitary corticotropin (plasma ACTH) and adrenocorticotropic hormone assays. Adrenocorticotropic hormone assays include serum cortisol, urinary free cortisol (UFC), urinary 17-ketosteroids (17-KS), and urinary 17-ketogenic steroids (17-KGS). Hospitals that are able to do so recommend that the test be performed within 7 days prior to the first dose, prior to the 3rd cycle of administration, and at the preoperative evaluation visit. (Hospitals that are able to do so may perform this test or some of these indices).
[16] Pregnancy tests are performed only on women of childbearing age, during the screening period and at the postoperative evaluation visit.
[17] 10 ml of venous blood, 5 ml of EDTA anticoagulation tube and 5 ml of red separator gel procoagulant tube, totaling 10 ml; of which the EDTA anticoagulation tube blood sample processing: 1000 g, centrifuged for 10 minutes, then take the supernatant and put it into the freezing tube; red separator gel procoagulant tube blood sample processing: 3000 rpm, centrifuged for 10 minutes, then take the supernatant and put it into the freezing tube; the freezing tube will be put into the refrigerator at -80 degrees Celsius to store. Venous blood will be collected and stored during the screening period, before each cycle of drug administration and preoperative evaluation (hospitals with conditions can collect this specimen).
[18] Quality of life scores were scored using the scale in Annex 3 and were scored during the screening period, before administration of medication at cycles 2, 3 and 4, at the preoperative assessment, and at the postoperative assessment visit.
[19] Imaging test arrangements

- PET-CT was prioritized during the screening period (accepting PET-CT results from qualified outside hospitals within 28 days). If subjects refused PET-CT, enhanced CT of the chest, MRI of the head to rule out brain metastasis, ultrasound of the abdomen to rule out abdominal metastasis, and bone scanning was required when bone metastasis was suspected;
- Enhanced CT of the chest is required before starting drug therapy in cycles 3 and 4;
- Preoperative evaluation was performed with enhanced CT of the chest, along with MRI of the head to rule out brain metastasis, ultrasound of the abdomen to rule out abdominal metastasis, and bone scanning when bone metastasis was suspected.
- In Arm A, for patients with squamous cancer whose chemotherapy regimen was gemcitabine (d1 and d8 dosing regimens), each imaging study was scheduled 3 weeks after the last gemcitabine dose;
- Postoperative evaluation and postoperative follow-up should be performed by chest plain CT; postoperative follow-up should be performed by whole-body bone scanning if there is bone pain, and cranial MRI should be performed if brain metastasis is suspected.
- Imaging should continue for up to 5 years from the time of surgery or until imaging confirms disease progression or disease recurrence.

[20] Adverse events were collected from the time of signing the informed consent form up to 90 days after the last dose of Camrelizumab or 30 days after the last dose of Apatinib, whichever was longer.
[21] Cycle 2 will be completed with or without cycle 3 at the discretion of the investigator, and likewise cycle 4 will be based on the effectiveness of cycle 3.
[22] Pathologic evaluation: Detailed pathologic diagnosis and evaluation is required after lung cancer resection to document postoperative pathologic response.
[23] Subjects with disease recurrence within 5 years or who have not relapsed at the end of the study (after 5 years postoperatively) are contacted (by visit or by phone) every 6 months ± 14 days in years 1-2 after postoperative evaluation, and every 6 months ± 14 days in years 3-5 to obtain information about the subject's overall survival and subsequent treatment at the end of the study Information.

List of Abbreviations

Abbreviations and full spelling	Definition
AE (Adverse Event)	不良事件
AKP (Alkline Phosphatase)	碱性磷酸酶
ALT (Alanine Amiotransferase)	丙氨酸氨基转移酶（谷丙转氨酶）
ANC (Absolute Neutrophil Count)	中性粒细胞计数
APTT (Activated Partial Thromboplastin Time)	活化部分凝血活酶时间
AST (Aspartate Aminotransferase)	天冬氨酸氨基转移酶（谷草转氨酶）
BIL (Bilirubin)	胆红素
BUN (Blood Urea Nitrogen)	尿素氮
Cr (Creatinine)	肌酐
CCr (Creatinine Clearance)	肌酐清除率
CR (Complete Response)	完全缓解
CRF (Case Report Form)	病例报告表
CT (Computed Tomography)	计算机 X 射线断层扫描
CTC AE (Common Terminology Criteria for Adverse Events)	不良事件通用术语标准
CFDA (China Food and Drug Administration)	国家食品药品监督管理总局
ECOG (Eastern Cooperative Oncology Group)	东部肿瘤协作组身体状况评分标准
ECG (Electro Cardio Gram)	心电图
EORTC (The European Organization for Research and Treatment for Cancer)	欧洲癌症研究与治疗组织
Fbg (Fibrinogen)	纤维蛋白原
GCP (Good Clinical Practice)	药物临床试验质量管理规范
FAS (Full Analysis Set)	全分析集
Glu (Glucose)	葡萄糖
Hb (Hemoglobin)	血红蛋白
HR (Hazard Ratio)	风险比
INR (International Normalized Ratio)	国际标准化比率
MRI (Magnetic Resonance Imaging)	核磁共振
MPR (major pathologic response)	主要病理缓解
MTD (Maximum Tolerated Dose)	最大耐受剂量
NSCLC (Non-small Cell Lung Cancer)	非小细胞肺癌

**Abbreviations and full spelling**	**Definition**
ORR (Objective Response Rate)	客观缓解率
PD (Progressive Disease)	疾病进展
pCR (Pathological complete response)	病理完全缓解
PI (Principal Investigator)	主要研究者
PLT (Platelets)	血小板
PK (Pharmacokinetics)	药代动力学
PD (Pharmacodynamics)	药效学
PR (Partial Response)	部分缓解
PS (Performance Status)	体能状态
PT (Prothrombin Time)	凝血酶原时间
RBC (Red Blood Cell)	红细胞
RECIST (Response Evaluation Criteria in Solid Tumor)	实体瘤疗效评价标准
γ-GT (γ- Glutamyltransferase)	γ-谷氨酰转移酶
SAE (Serious Adverse Event)	严重不良事件
SAS (Safety Analysis Set)	安全性分析集
SCr (Serum Creatinine)	血清肌酐
SD (Stable Disease)	疾病稳定
TT (Thrombin Time)	凝血酶时间
ULN (Upper Limit of Normal)	正常值上限
WBC (White Blood Cell)	白细胞

1. Background and rationale for the study

1.1 Epidemiology of lung cancer

Lung cancer is one of the most common malignant tumors, and according to the latest data in 2018, lung cancer incidence (11.6%) and mortality (18.4%) ranked first among malignant tumors[1]. In China, the data in 2015 showed that the incidence rate (19.6%) and mortality rate (26.0%) of lung cancer also ranked the first among all malignant tumors, compared with the incidence rate (13.0%) and mortality rate (18.0%) in 2007, which ranked the first in male incidence rate and mortality rate, and the second in female incidence rate (lower than that of breast cancer), and the first in mortality rate[2-4]. About 25% of the world's lung cancer patients are in the Asia-Pacific region, and lung cancer has become the leading cause of cancer deaths.

1.2 Current status of neoadjuvant therapy for lung cancer

Non-small cell lung cancer (NSCLC) is the most common pathological type of lung cancer, accounting for 80-85% of all lung malignancies[5]. The most common histological types of NSCLC are adenocarcinoma and squamous cell carcinoma. Among NSCLC patients, 20% are stage I or II, 30% are stage III, and 50% are stage IV[6]. The 5-year survival rate for patients with stage I NSCLC is more than 60%, that for patients with stage II NSCLC is more than 50%, and that for patients with stage III NSCLC is about 30%[7]. Surgery is still the main treatment for stage I-III lung cancer. The surgical resection rate for stage II and IIIA NSCLC patients is less than 30%, and the 5-year survival rate after surgery is very low, with high recurrence rate in a short period of time after surgery. For stage II-III NSCLC, according to the NCCN guidelines, in addition to radical surgery with mediastinal lymph node dissection, patients should receive platinum-based adjuvant postoperative chemotherapy and/or radiotherapy. Despite the effectiveness of surgical treatment, approximately 50% of patients with stage IB and 70% of patients with stage II NSCLC will recur and ultimately die from the cancer[6]. The primary cause of postoperative recurrence is the presence of micrometastases, and the approach to eradicate micrometastatic disease and minimize the risk of recurrence is adjuvant or neoadjuvant chemotherapy. Overall data from several studies (Table 1) suggest that adjuvant platinum-based chemotherapy is beneficial to the survival of patients with stage II disease. Surgery generally precedes

adjuvant therapy for patients with resectable non-small cell lung cancer, but survival advantages of postoperative adjuvant therapy remain limited at present.

Neoadjuvant therapy has the effect of shrinking tumors, increasing the rate of complete surgical resection, and eradicating micrometastases, and has become one of the treatment schemes nowadays. A meta-analysis based on seven trials with a total of 988 patients showed that preoperative neoadjuvant chemotherapy improved overall survival (20% at 5 years compared with 14% without neoadjuvant chemotherapy), and this improvement in survival was similar to the results of the meta-analysis that focused on adjuvant chemotherapy[8]. The LACE meta-analysis of the adjuvant and neoadjuvant treatment trial, which used cisplatin-based chemotherapy, showed a 5% survival advantage over 5 years from the start of adjuvant chemotherapy, with the greatest survival advantage in stage II and stage IIIA patients[9]. This benefit is still limited and the toxicity is great (incidence >60%)[9]. So new treatment strategies are still needed to further improve survival.

Table 1. Adjuvant studies in non-small cell lung cancer

Study	Stage	Treatment	Sample Size	5-year OS	HR	P-value
**ALPI**	I - III	Surgery + (mitomycin + vincristine + cisplatin)	603 vs 601	45% vs 50%	0.96	0.59
**IALT**	I - III	Surgery + cisplatin combination chemotherapy	935 vs 932	40% vs 44.5%	0.86	<0.03
**ANITA**	IB - IIIA	Surgery + (cisplatin + vincristine)	433 vs 407	43% vs 51%	0.80	0.017
**BLT**	I - IIIA	Surgery + cisplatin combination chemotherapy	189 vs 192	58% vs 60%	1.02	0.90
**NCIC/JBR10**	IB - II	Surgery + (cisplatin + vincristine)	240 vs 242	54% vs 69%	0.69	0.03
**CALGB**	IB	Surgery + (carboplatin + paclitaxel)	171 vs 173	57% vs 59%	0.80	0.10

1.3 Immunotherapy of tumors

Host immunity is the basis for suppression of cancers, while evasion of host immunity by tumor cells is an important pathway in the development of cancers. The three stages of tumor immunoediting are: elimination (the host immune system responds to tumor neoantigens and destroys tumor cells), homeostasis (residual host

immunity suppresses the continued growth and metastasis of immune-evading tumor cells), and escape (tumor cells overcome immune control and develop into clinically visible cancers). The presence of clinically visible tumors indicates the inability of the host immune system to recognize and destroy early cancers due to the induction of immune tolerance among tumor-specific T cells and the expression of immunosuppressive ligand checkpoints[10]. These ligands bind to receptors on T cells, down regulating effects such as cytokine production and killing activity. Therefore, strategies to enhance host anti-tumor immunity have potential for long-term tumor control or even cure if a durable immune response can be generated.

Programmed cell death protein 1 (PD-1 or CD279), mainly expressed on activated T cells, B cells and myeloid cells, is a 55-kD type I transmembrane protein and a member of the CD28 family of T-cell co-stimulatory receptors, which also includes CD28, CTLA-4, ICOS and BTLA[11]. Two specific PD-1 ligands have been identified: PD-L1 (also known as B7-H1 or CD274) and PD-L2 (also known as B7-DC or CD273), which are predominantly expressed on antigen-presenting cells. Both PD-L1 and PD-L2 have been shown to down-regulate T-cell activation in both mouse and human systems upon binding to PD-1[12-14]. Aberrant expression of PD-L1 by tumor cells has been reported in many human NSCLC[15,16]. Patients with high tumor and/or lymphocyte PD-L1 expression are 4.5 times more likely to die from cancer than patients with low PD-L1 expression, possibly due to immune evasion resulting from high PD-L1 expression. This hypothesis is supported by independent studies that have shown that tumor cell expression of PD-L1 increases apoptosis of activated tumor-specific T cells in vitro[17], while PD-L1 expression protects tumor cells from effector T cell-induced apoptosis[18]. Preclinical data identified a blocking effect of PD-1 in both PD-L1 expressing positive and negative tumors, suggesting a possible role in immune initiation and the tumor microenvironment[19-21].

1.4 Advances in immuno-neoadjuvant therapy in lung cancer

Clinical studies have demonstrated that anti-PD-1 drugs can effectively improve the survival of patients with advanced NSCLC, and anti-PD-1 monotherapy or combination chemotherapy has become the first-line standard of care for patients with driver-negative NSCLC. Recent studies have shown that immunotherapy is also emerging as a new option for neoadjuvant treatment of lung cancer. The CheckMate159 study[22] showed that Nivolumab as a neoadjuvant therapy achieved major pathological

response (MPR) in 9 out of 20 cases of completely resected tumors in patients with resectable NSCLC, with the MPR rate of 45%. In 2019, LCMC3 study[23] showed that of the 77 evaluable patients who received Atezolizumab neoadjuvant therapy and underwent surgical resection, 4 (5%) patients achieved a pathological complete response (pCR), and 15 (19%) patients achieved an MPR. Another exploratory phase II study in Chinese population in 2019 included 40 patients with stage IA-IIIB NSCLC receiving 2 cycle Sintilimab neoadjuvant therapy[24]. Of the 37 patients who underwent surgical resection, 15 (40.5%) patients achieved MPR and 6 (16.2%) patients achieved pCR.

In addition, immune-combination chemotherapy or immune-combination immunization has also achieved good results in neoadjuvant treatment of lung cancer. In 2019, the phase II clinical study NEOSTAR[25-26] reported the results of patients treated with neoadjuvant Nivolumab monotherapy (N) or neoadjuvant Eptilizumab in combination with Nivolumab (NI). In surgically resected patients, the overall MPR + pCR was 30%, and was 19% in the N group and 44% in the NI combination group. The overall ORR was 20%, with an ORR of 22% in the N group and 19% in the NI combination group. Safety assessments showed that neoadjuvant therapy was well tolerated, with the majority of treatment-related adverse events (TRAEs) being grade 1-2. The incidence of grade 1-2 TRAEs in the overall population and in the two treatment groups were 59%, 27.1%, and 72.9%, respectively. Chemotherapy with Nivolumab in combination with Paclitaxel plus platinum showed excellent results, as reported at ASCO 2019. The NADIM study[27] was a phase II, single-arm, open, multicenter study enrolling patients with locally advanced resectable stage IIIA N2 NSCLC. Patients received Nivolumab plus chemotherapy as neoadjuvant therapy following Nivolumab adjuvant therapy for 1 year. A total of 46 patients were enrolled in the study, 46 with imaging response and 41 with pathologic response. Neoadjuvant chemotherapy combined with immunotherapy was well tolerated. No patients were inoperable due to disease progression or toxicity. In the overall clinical response rate assessment, there were 3 patients with CR (6.5%), 33 patients with PR (72%), and 8 patients with SD (17.5%). In the postoperative pathologic response rate assessment, 35 (85.36%) patients achieved MPR, 25 (71.4.0%) patients achieved pCR, and 6 (14.6%) patients achieved partial response. The overall ORR was 78.5% according to RECIST criteria. Chemotherapy in combination with immunotherapy during the neoadjuvant phase of treatment demonstrated superior anti-tumor efficacy in patients with locally

advanced, potentially resectable NSCLC, achieving pathologic complete response rates that exceeded expectations. In a recent study published in 2020[28], a total of 30 patients with stage IB-IIIA lung cancer were enrolled, receiving 2-4 cycles of neoadjuvant therapy with Atezolizumab in combination with paclitaxel plus carboplatin, with 10 patients ultimately achieving a pCR (33%) and 17 achieving an MPR (57%). In addition, several phase III clinical studies of neoadjuvant therapy PD-1/PD-L1 plus chemotherapy for lung cancer are underway.

1.5 Camrelizumab

Camrelizumab (code SHR-1210) is a humanized anti-PD1 IgG4 monoclonal antibody, an immune checkpoint inhibitor drug that binds with high affinity to PD-1 (B7- H1), blocks its interaction, and increases proliferation and cytokine secretion of tumor antigen-specific T cells[29]. At the World Conference on Lung Cancer (WCLC) in September 2019, Prof. Yilong Wu announced a phase II umbrella study of Camrelizumab for the treatment of treated advanced/metastatic NSCLC with different PD-L1 expression levels[30]. The study included 259 patients with EGFR and ALK mutation-negative NSCLC that progressed during or after platinum-based two-agent chemotherapy, of which 229 patients were available for pathologic evaluation and were assigned to one of four subgroups based on PD-L1 expression level. Patients who were positive for EGFR or ALK mutations and had ≥50% PD-L1 expression, treated with at least a first-line tyrosine kinase inhibitor (TKI), were also included in the study. The data showed an ORR of 18.5%, a median PFS of 3.2 months (2.0 to 3.4), and a median OS of 19.4 months (11.6 to not reached) for all patients with varying PD-L1 expression levels. In addition, responding patients had long sustaining responses with a median duration of response (DoR) of 15.1 months (5.5 months to not reached). Data from subgroup analyses showed a positive correlation between longer progression-free survival with PD-L1 expression, up to a maximum PFS of 7.1 months. In terms of safety, the incidence of treatment-related adverse events (TRAEs) of any grade was 87%, with a 17.1% incidence of grade ≥3 TRAEs, 13.7% of patients experienced serious TRAEs, 13.7% of patients experienced dose adjustments or interruptions due to TRAEs, and 4.8% of patients experienced treatment discontinuation due to TRAEs. In addition, Caicun Zhou announced the data from the first Phase III study of first-line immunochemotherapy for NSCLC (advanced/metastatic non-squamous non-small cell lung cancer) in the Chinese population.[31] The combination of Camrelizumab and

chemotherapy significantly prolonged patients' median progression-free survival (PFS) to 11.3 months, compared to 8.3 months in the chemotherapy group. The objective response rate was 60% in immunochemotherapy group, more than 20% higher than that of chemotherapy, and the risk of progression was reduced by nearly 39%. Meanwhile, in terms of treatment safety, the adverse events of first-line treatment of advanced/metastatic non-squamous non-small cell lung cancer with Camrelizumab in combination with chemotherapy are more controllable. Clinical results showed that the combination of Camrelizumab and chemotherapy had a significant improvement in the overall survival benefit of lung cancer patients. Based on the results of this study, in June 2020, the NMPA formally approved the use of Camrelizumab in combination with platinum-doublet chemotherapy for the first-line treatment of driver-negative advanced or recurrent non-squamous non-small cell lung cancer.

1.6 Apatinib

In recent years, the important role of angiogenesis for tumors begun to attract attention as the study of the biological lineage of tumors develops. Tumor angiogenesis is important for tumor growth, invasion and metastasis[32]. The abundant vascular beds on the pleural surface are the main pathway for tumor metastasis. Tumor angiogenic pathways, including NSCLC, are considered important therapeutic targets. Studies have identified vascular endothelial growth factor receptor (VEGFR), platelet-derived growth factor receptor (PDGFR), fibroblast growth factor receptor (FGFR) and the crosstalk between them are the main mechanisms to regulate tumor neovascularization. The role of VEGFR pathway is to promote vascular neovascularization, endothelial cell growth, and vascular permeability; the role of PDGFR is to promote the growth and differentiation of endothelial cells, smooth muscle cells and fibroblasts; and the role of FGFR is to promote cell growth and survival, vascular neovascularization, and recruitment of pericytes[33]. To address the three major pathways of tumor angiogenesis in NSCLC, researchers have developed antiangiogenic drugs to inhibit angiogenesis in NSCLC, including monoclonal antibodies, small molecule vascular endothelial growth factor receptor-tyrosine kinase inhibitors (VEGFR-TKIs), and VEGFR-trapping agents (VEGFR-trap)[34]. These drugs can block or inhibit angiogenic signaling pathways such as VEGFR to exert their anti-angiogenesis effects. Apatinib is a small molecule vascular endothelial growth factor receptor 2 (VEGFR-2) tyrosine kinase inhibitor that inhibits tumor angiogenesis. The current treatment regimen of combining anti-

angiogenic drugs has been used in a variety of malignancies, including advanced non-small cell lung cancer, metastatic colorectal cancer, metastatic breast cancer, metastatic renal cancer, and has achieved good efficacy[35]. A multicenter, randomized controlled phase II clinical study found that Apatinib significantly prolonged survival in patients with advanced non-squamous NSCLC who had failed second-line and beyond[36]. A retrospective study found that the combination of the antiangiogenic drug Apatinib with chemotherapy resulted in significant disease response and progression-free survival compared to chemotherapy alone[37,38]. Several prospective clinical studies of the combination of antiangiogenic drugs with chemotherapy are currently underway (NCT03256721, NCT03758677, NCT03201146, etc.). In addition, studies have found that combining antiangiogenic drugs with targeted therapies significantly improves progression-free survival compared to single reagent targeted therapies[39]. Current studies have shown that combining antiangiogenic drugs with other antitumor therapies has benefited many patients.

Results from a phase Ib clinical study of the anti-PD-1 drug Camrelizumab in combination with Apatinib in advanced nonsquamous NSCLC that failed multiple lines of therapy were presented at the 2018 American Society of Clinical Oncology (ASCO) conference[40]. 27 patients with NSCLC who failed second-line or systemic chemotherapy were treated with Camrelizumab (200 mg every 2 weeks) in combination with Apatinib (250 or 375 mg/d) until disease progression or toxicity intolerance. Results showed that patients had an objective response rate (ORR) of 41.2% and a disease control rate (DCR) of 94.1%. The latest data presented at the 2019 ASCO from a phase II clinical study[41] of Apatinib in combination with Camrelizumab in 96 patients with advanced NSCLC who had undergone ≥1 line of prior systemic chemotherapy revealed an ORR of 30.8% and a median PFS of 5.9 months. This study reveals that Apatinib combined with immunotherapy showed a powerful synergistic anti-cancer effect.

In summary, surgical resection is still the main treatment for patients with stage II-III lung cancer. However, nearly 50% of NSCLC patients will still relapse and die. Neoadjuvant immunotherapy can effectively reduce tumor micrometastasis and improve long-term survival. This study will explore the efficacy and safety of Camrelizumab in combination with chemotherapy or Apatinib in the neoadjuvant treatment of patients with stage II-III resectable and initially unresectable lung cancer, providing new evidence for neoadjuvant immunotherapy in locally advanced NSCLC.

2. Study Objectives

To evaluate the efficacy and safety of neoadjuvant anti-PD-1 agent Camrelizumab in combination with Apatinib or chemotherapy for resectable and initially unresectable stage II-III NSCLC.

The primary endpoint of this study was the MPR rate, which was the ratio of subjects experiencing major pathologic response to the total number of subjects undergoing surgery. MPR was defined as no more than 10% of residual viable tumor cells in the tumor bed in the postoperative specimen.

Secondary study endpoints included pCR rate, ORR, EFS, 1-year EFS rate, 2-year EFS rate, DFS, 1-year DFS rate, 2-year DFS rate, OS, quality of life, R0 resection rate, and safety.

3. Study Design

3.1 Research design and program

This is a prospective, open, multicenter study divided into two treatment arms, arm A with neoadjuvant Camrelizumab in combination with chemotherapy and arm B with neoadjuvant Camrelizumab in combination with Apatinib.

3.2 Selection and Withdrawal of the Subject Population

3.2.1 Enrollment Criteria

Arm A: Camrelizumab + platinum-doublet chemotherapy

(1) Age ≥ 18 years and ≤ 75 years;

(2) ECOG PS scores of 0-1;

(3) Treatment-naïve patients with histologically or cytologically confirmed stage II-III NSCLC (according to the IASLC Staging Manual in Thoracic Oncology, 8th Edition);

(4) At least one measurable lesion (according to RECIST 1.1 criteria);

(5) Life expectancy of at least 12 weeks.

(6) Other major organs (liver, kidneys, blood system, etc.) are normal.

• Hemoglobin ≥ 90 g/L (no blood transfusion, no use of hematopoietic factors and no drug correction within 2 weeks prior to first dose);

• Absolute neutrophil count (ANC) ≥ 1.5×10^9 /L;

• Platelet count ≥ 100×10^9 /L;

• Total bilirubin ≤ 1.5 times the upper limit of normal;
• ≤ 2.5 times the upper limit of normal values for alanine aminotransferase, glutamine aminotransferase, and alkaline phosphatase;
• Serum creatinine ≤ 1.5 times the upper limit of normal; and endogenous creatinine clearance ≥ 60 ml/min;
• International normalized ratio (INR) of prothrombinogen time ≤ 1.5 and activated partial thromboplastin time (APTT) ≤ 1.5 times the upper limit of normal in patients who have not received anticoagulation therapy;

(7) No systemic metastases (including M1a, M1b, M1c);

(8) Completely resectable or initially unresectable disease. Initially unresectable disease is evaluated by a multidisciplinary clinical team (MDT) and is defined as meeting one of the following three conditions: ① Tumor invading vital structures, such as large blood vessels, the trachea or primary bronchus, but curative resection after tumor downgrading by neoadjuvant therapy was possible as judged by a preoperative assessment; ② Clinically confirmed lymph nodes with multistation metastasis or bulky fusion, and patients could tolerate hilar and mediastinal lymph node dissection after tumor downgrading by neoadjuvant therapy as determined by a preoperative evaluation; ③ According to preoperative evaluation, even if pneumonectomy was performed, especially right pneumonectomy, R0 resection may not be achieved;

(9) Adequate lung function must be available for the intended pulmonary resection procedure;

(10) Female subjects of childbearing potential must have a negative pregnancy test (serum or urine) performed within 72 hours prior to initiation of study drug administration and be willing to use a medically approved effective method of contraception (e.g., IUD, birth control pills, or condoms) for the duration of the study and for 90 days after final study drug administration; for male subjects whose partner is a female of childbearing potential, agree to use an effective method of contraception or have been surgically sterilized for the duration of the study and for 90 days after last drug administration.

(11) Subjects voluntarily enrolled in this clinical study and signed an informed consent form.

Arm B: Camrelizumab + Apatinib

(1) Age ≥ 18 years and ≤ 75 years;

(2) ECOG PS scores of 0-1;

(3) Treatment-naïve patients with histologically or cytologically confirmed stage II-III NSCLC (according to the IASLC Staging Manual in Thoracic Oncology, 8th Edition);

(4) Sufficient tumor tissue is available to detect PD-L1 expression and PD-L1 is ≥1%;

(5) At least one measurable lesion (according to RECIST 1.1 criteria);

(6) Life expectancy of at least 12 weeks.

(7) Other major organs (liver, kidneys, blood system, etc.) are normal.

• Hemoglobin ≥ 90 g/L (no blood transfusion, no use of hematopoietic factors and no drug correction within 2 weeks prior to first dose);

• Absolute neutrophil count (ANC) ≥ 1.5×10^9 /L;

• Platelet count ≥ 100×10^9 /L;

• Total bilirubin ≤ 1.5 times the upper limit of normal;

• ≤ 2.5 times the upper limit of normal values for alanine aminotransferase, glutamine aminotransferase, and alkaline phosphatase;

• Serum creatinine ≤ 1.5 times the upper limit of normal; and endogenous creatinine clearance ≥ 60 ml/min;

• International normalized ratio (INR) of prothrombinogen time ≤ 1.5 and activated partial thromboplastin time (APTT) ≤ 1.5 times the upper limit of normal in patients who have not received anticoagulation therapy;

(8) No systemic metastases (including M1a, M1b, M1c);

(9) Completely resectable or initially unresectable disease. Initially unresectable disease is evaluated by a multidisciplinary clinical team (MDT) and is defined as meeting one of the following three conditions: ① Tumor invading vital structures, such as large blood vessels, the trachea or primary bronchus, but curative resection after tumor downgrading by neoadjuvant therapy was possible as judged by a preoperative assessment; ② Clinically confirmed lymph nodes with multistation metastasis or bulky fusion, and patients could tolerate hilar and mediastinal lymph node dissection after tumor downgrading by neoadjuvant therapy as determined by a preoperative evaluation; ③ According to preoperative evaluation, even if pneumonectomy was performed, especially right pneumonectomy, R0 resection may not be achieved;

(10) Adequate lung function must be available for the intended pulmonary resection procedure;

(11) Female subjects of childbearing potential must have a negative pregnancy test

(serum or urine) performed within 72 hours prior to initiation of study drug administration and be willing to use a medically approved effective method of contraception (e.g., IUD, birth control pills, or condoms) for the duration of the study and for 90 days after final study drug administration; for male subjects whose partner is a female of childbearing potential, agree to use an effective method of contraception or have been surgically sterilized for the duration of the study and for 90 days after last drug administration.

(12) Subjects voluntarily enrolled in this clinical study and signed an informed consent form.

3.2.2 Exclusion criteria

Arm A: Camrelizumab + platinum-doublet chemotherapy

(1) Any systemic anticancer treatment for NSCLC, including surgical treatment, local radiotherapy, cytotoxic drug therapy, targeted therapy, immunotherapy, and traditional Chinese medicine antitumor therapy (excluding treatment for malignant tumors that have been previously eradicated and have been free of recurrence and metastasis for ≥5 years);

(2) Non-squamous cancer patients with EGFR activating mutations or ALK fusion mutations;

(3) Patients with other malignancies within five years prior to the start of this trial, excluding cured carcinoma in situ of the cervix, basal cell carcinoma of the skin, or squamous cell carcinoma of the skin;

(4) Patients with any active autoimmune disease or history of autoimmune disease (e.g., uveitis, enteritis, hepatitis, pituitary gland inflammation, vasculitis, myocarditis, nephritis, hyperthyroidism, hypothyroidism (may be included after hormone replacement therapy), tuberculosis); patients with asthma in childhood that does not require any intervention in adulthood, and patients with a skin disorder that has been in complete response that does not require systemic therapy (e.g., vitiligo, psoriasis, or alopecia areata) may be included; patients requiring medical intervention with bronchodilators may not be included;

(5) Exclude those with the evidence of previous or current pulmonary fibrosis, interstitial pneumonitis, pneumoconiosis, radiation pneumonitis, drug-induced pneumonitis, imaging-confirmed active pneumonitis, and severely impaired lung function;

(6) Subjects who have been systemically treated with corticosteroids (>10 mg/day prednisone or other equivalent hormone) or other immunosuppressive agents within 2 weeks prior to the first dose of study drug. Inhaled or topical corticosteroids and adrenal hormone replacement therapy at doses ≤ 10 mg/day prednisone efficacy dose are permitted in the absence of active autoimmune disease;

(7) Allergy to the test drug;

(8) Comorbid HIV infection or active viral hepatitis;

(9) Pregnant or lactating women; subjects of childbearing potential who are unwilling or unable to use effective contraception;

(10) Those who suffer from neurological or mental illnesses who cannot cooperate;

(11) Other circumstances judged by the investigator to be inappropriate for enrollment.

Arm B: Camrelizumab + Apatinib

(1) Any systemic anticancer treatment for NSCLC, including surgical treatment, local radiotherapy, cytotoxic drug therapy, targeted therapy, immunotherapy, and traditional Chinese medicine antitumor therapy (excluding treatment for malignant tumors that have been previously eradicated and have been free of recurrence and metastasis for ≥5 years);

(2) Non-squamous cancer patients with EGFR activating mutations or ALK fusion mutations;

(3) Patients with other malignancies within five years prior to the start of this trial, excluding cured carcinoma in situ of the cervix, basal cell carcinoma of the skin, or squamous cell carcinoma of the skin;

(4) Patients with any active autoimmune disease or history of autoimmune disease (e.g., uveitis, enteritis, hepatitis, pituitary gland inflammation, vasculitis, myocarditis, nephritis, hyperthyroidism, hypothyroidism (may be included after hormone replacement therapy), tuberculosis); patients with asthma in childhood that does not require any intervention in adulthood, and patients with a skin disorder that has been in complete response that does not require systemic therapy (e.g., vitiligo, psoriasis, or alopecia areata) may be included; patients requiring medical intervention with bronchodilators may not be included;

(5) Exclude those with the evidence of previous or current pulmonary fibrosis, interstitial pneumonitis, pneumoconiosis, radiation pneumonitis, drug-induced pneumonitis, imaging-confirmed active pneumonitis, and severely impaired lung function;

(6) Subjects who have been systemically treated with corticosteroids (>10 mg/day prednisone or other equivalent hormone) or other immunosuppressive agents within 2 weeks prior to the first dose of study drug. Inhaled or topical corticosteroids and adrenal hormone replacement therapy at doses ≤ 10 mg/day prednisone efficacy dose are permitted in the absence of active autoimmune disease;
(7) Imaging (CT or MRI) shows that the tumor invades large blood vessels or has an indistinct border with blood vessels;
(8) Those who had an arterial/venous thrombotic event such as cerebrovascular accident (including temporary ischemic attack, cerebral hemorrhage, and cerebral infarction), deep vein thrombosis, and pulmonary embolism in the 6 months prior to enrollment;
(9) Have experienced clinically significant bleeding symptoms or have a definite bleeding tendency, such as gastrointestinal bleeding, bleeding gastric ulcer, etc., within 3 months prior to enrollment, or are receiving thrombolytic or anticoagulant therapy;
(10) Significant hemoptysis symptoms or daily hemoptysis of 2.5mL or more within 1 month prior to enrollment;
(11) Hypertension that is not well controlled by antihypertensive medication (systolic blood pressure ≥140 mmHg or diastolic blood pressure ≥90 mmHg); or myocardial ischemia or myocardial infarction of grade II or above, or poorly controlled cardiac arrhythmia (including QTc interval ≥450ms for men and ≥470ms for women); or cardiac insufficiency of grade III-IV according to the NYHA criteria; or left ventricular ejection fraction (LVEF) of <50% according to Doppler echocardiography;
(12) Those who have had major surgery on other systems or severe trauma within 2 months prior to the start of this trial;
(13) Urine routine suggesting urinary protein ≥ (++), or 24h urine protein volume ≥ 1g or severe hepatic or renal insufficiency;
(14) Uncontrollable pleural effusion, pericardial effusion, or ascites requiring repeated drainage;
(15) Allergy to the test drug;
(16) Comorbid HIV infection or active viral hepatitis;
(17) Pregnant or lactating women; subjects of childbearing potential who are unwilling or unable to use effective contraception;
(18) Persons with neurological or mental illnesses who are unable to cooperate;
(19) Other circumstances that the investigator considers inappropriate for enrollment.

3.2.3 Subject exit criteria

Subjects who experience one or more of the following will withdraw from treatment:
(1) The subject requests to withdraw from treatment or withdraws the informed consent;
(2) Disease progression confirmed by imaging;
(3) Poor subject compliance;
(4) The subject is unable to tolerate the toxicity;
(5) Loss to follow-up, death, or pregnancy event;
(6) Other circumstances that, in the opinion of the investigator, necessitate termination of treatment.

All subjects who withdraw from the study should document the reason for withdrawal on the case report form and in the subject's medical record.

All subjects withdrawn due to an adverse event or abnormal laboratory test should be followed until recovery or stabilization of the adverse event and the subsequent outcome of the event should be recorded. The investigator should be notified of any subject who dies during or within 28 days of completion of trial treatment. The cause of death must be recorded in detail on the Serious Adverse Event (SAE) report form within 24 hours of being informed of the death and reported to the ethics department.

3.2.4 Criteria for study termination

(1) Identified unintended, significant, or unacceptable risks to the subject.
(2) Research drug therapy is confirmed to be ineffective.
(3) Pre-existing efficacy results support early termination of the study.
(4) Significant mistakes in protocol are identified during the implementation of study.
(5) Completion of studies is extremely difficult due to significant lags in subject enrollment or frequent protocol deviations.
(6) Incomplete or unmeasurable data.

3.3 Research time

Estimated first subject enrollment: 2020.07
Estimated completion of enrollment: 2021.05
Estimated end of study: 2026.05

3.4 Treatment plan

Arm A: Camrelizumab + Platinum-doublet chemotherapy

Arm B: Camrelizumab + Apatinib Treatment

Subjects in both groups were followed up for a long period of time. Follow-up included routine examinations, CT scan of the chest, systemic ECT in case of bone pain, and cranial MRI if brain metastasis was suspected in the judgment of the investigator.

3.4.1 Treatment arrangements

Preoperative treatment:

(1) Arm A: Camrelizumab + Platinum-doublet chemotherapy

Camrelizumab: a fixed dose of 200 mg, intravenous drip over a period of 30-60 min, d1, every 3 weeks as a cycle, preoperative use of 2-4 cycles;

Platinum-containing two-drug chemotherapy: ① Squamous carcinoma: Carboplatin AUC5 d1 + Gemcitabine 1000mg/m^2 , d1, d8 (or Paclitaxel 135~175 mg/m^2 , d1; or Docetaxel 60~75 mg/m^2 , d1; or Albumin Paclitaxel 260 mg/m^2, d1) ② Non-squamous carcinoma: Carboplatin AUC5 d1, Pemetrexed 500mg/m^2, d1), 3 weeks as a treatment cycle, and 2-4 cycles of preoperative administration.

All subjects are required to complete at least 2 cycles of neoadjuvant therapy with Camrelizumab in combination with chemotherapy, with assessment at the end of the 2nd cycle based on enhanced CT of the chest, and continuation of the 3rd cycle at the discretion of the investigator, and continuation of the 4th cycle at the end of the 3rd cycle based on enhanced CT of the chest. A preoperative evaluation is performed 3-4 weeks after the final dose, and subjects undergo surgery within 30 days of the preoperative evaluation. All subjects receive up to 4 cycles of neoadjuvant therapy with Camrelizumab in combination with chemotherapy, and subjects assessed as inoperable by the investigator after 4 cycles of neoadjuvant therapy are required to document in detail the reason for inoperation and alternative treatment options.

(2) Arm B: Camrelizumab + Apatinib

Camrelizumab: a fixed dose of 200 mg, intravenous drip over a period of 30-60 min, d1, every 3 weeks as a cycle, preoperative use of 2-4 cycles;

Apatinib: 250 mg/day, orally once daily for 3 weeks as a treatment cycle, 2-4 cycles of preoperative administration.

All subjects are required to complete at least 2 cycles of neoadjuvant therapy with Camrelizumab in combination with Apatinib, with assessment at the end of the 2nd cycle based on enhanced CT of the chest, and continuation of the 3rd cycle at the discretion of the investigator, and continuation of the 4th cycle at the end of the 3rd

cycle based on enhanced CT of the chest. A preoperative evaluation is performed 3-4 weeks after the final dose, and subjects undergo surgery within 30 days of the preoperative evaluation. All subjects receive up to 4 cycles of neoadjuvant therapy with Camrelizumab in combination with chemotherapy, and subjects assessed as inoperable by the investigator after 4 cycles of neoadjuvant therapy are required to document in detail the reason for inoperation and alternative treatment options.

Radical surgery:

Surgical resection with a procedure that meets non-palliative complete resection of all lesions.

Postoperative evaluation:

30 days ± 10 days postoperatively, a postoperative evaluation was performed and a determination was made as to whether adjuvant therapy would be performed.

Postoperative adjuvant therapy (observational study):

The investigators decide whether to carry out adjuvant chemoradiotherapy at 4-8 weeks postoperatively according to the subject's postoperative status and pathological results, or according to the response to neoadjuvant regimen (for those who have responded well to neoadjuvant therapy). Under the recommendations of the doctor and the subject's willingness, the adjuvant treatment scheme is conducted in trial hospital and/or outside hospitals.

Postoperative follow-up:

Postoperative visit is conducted every 3 months ± 14 days for first 2 years, and every 6 months ± 14 days for years 3-5, including routine examinations, CT scan of the chest, systemic ECT in case of bone pain, and cranial MRI if brain metastasis is suspected in the judgment of the investigator. Subjects with recurrence of disease within 5 years or without recurrence at the end of the study (5 years after surgery) will be contacted (by visit or by phone) every 6 months ± 14 days to obtain information about their survival and subsequent treatment after the end of the study. Follow-up includs survival status, time to relapse, date and cause of death if death occurs, and other antitumor therapy received after progression.

3.4.2 Neoadjuvant drug monitoring and management

MONITORING: All subjects should have complete blood cell count tests, including platelet count. Subjects should be monitored for baseline values and recovery, which should be checked before each dose administration and at the end of the cycle.

Biochemical tests should also be performed prior to each dose to assess hepatic and renal function. The next treatment cycle can be started only when the following conditions are met: neutrophil count ≥1.5 x 10^9 /L, platelet count ≥100 x 10^9 /L, creatinine clearance ≥45 mL/min, total bilirubin is ≤1.5 times the upper limit of normal values, and alkaline phosphatase (AP), aspartate aminotransferase (AST or GOT), and alanine aminotransferase (ALT or SGOT) ≤3 times the upper limit of normal values.

Dosage adjustments are made at the discretion of the investigator based on the degree of drug-related toxicity and possible efficacy experienced by the subjects:

(1) The occurrence of hepatic-related adverse events to Camrelizumab management recommendations:

- Immune-related hepatitis has been reported in subjects treated with Camrelizumab. Liver function (e.g., aminotransferases and total bilirubin) and symptoms associated with hepatitis should be monitored during the course of treatment, and rule out other infections and disease-related etiologies.
- Treatment with corticosteroids [0.5-1 mg/kg/d (for grade 2 hepatitis) and 1-2 mg/kg/d (for grade ≥3 hepatitis) prednisone or equivalent doses of other corticosteroids, followed by a gradual dose decrement] depending on the degree of hepatic function abnormality. Suspension or permanent discontinuation of the drug was adopted according to the severity of hepatic function abnormalities.

Degree	Symptoms/Signs	Treatment recommendations	Management
1	AST, ALT ≤ ULN ~3× ULN<br>Total bilirubin ≤ ULN ~1.5× ULN	Continue with the medication.	Continue to monitor liver function. If liver function is stable, reduce frequency of testing appropriately.
2	AST, ALT > 3× ULN ~ ≤ 5× ULN<br>Total bilirubin > 1.5× ULN ~ ≤ 3× ULN	Suspension of use. Monitor liver function every 3 days.	• Oral prednisone 0.5-1 mg/kg/day, slowly reduce if liver function improves, total course at least 4 weeks.<br>• Liver biopsy is optional.<br>• Prednisone dose reduced to ≤10mg/day and grade ≤1 hepatotoxicity, with reintroduction of Camrelizumab.
3	AST, ALT > 5× ULN ~ ≤ 20× ULN<br>Total bilirubin > 3× ULN ~ ≤10× ULN	Discontinue medication immediately. Permanent termination of medication.<br>• Monitor liver function every 1 to 2 days.	• When hepatic function improves to grade 2, equivalently switch to oral prednisone and slowly reduce; total course at least 4 weeks.<br>• If liver function does not improve even after 3 days, consider adding mescaline (500-1000 mg BID).<br>• Liver specialist consultation.

4	AST, ALT > 20× ULN<br>Total bilirubin > 10× ULN	• Hepatology specialist consultation.<br>• CT scan or ultrasound check of the liver.<br>• Intravenous prednisone at 1 to 2 mg/kg/day or other equivalent corticosteroids given intravenously	• CT scan or ultrasound check of the liver.<br>• Infliximab is not recommended.<br>• Liver biopsy is optional.

(2) The occurrence of Camrelizumab immune-related myocarditis and management recommendations:

- Immune-related myocarditis is a predictable adverse event resulting from PD-1 monoclonal antibody. The incidence of myocarditis is <1% according to the comparable drug instruction.
- The incidence of immune-related myocarditis with Camrelizumab was 0.2%, similar to that of its counterpart.

Degree	Symptoms/Signs	Treatment recommendations	Management
**1**	Asymptomatic with laboratory tests (e.g., BNP natriuretic peptide) or cardiac imaging abnormalities.	Continue with the medication.	• Monitoring of changes in test values and clinical manifestations of subjects.
**2**	Symptoms when mild to moderate physical activity or exertion.	Suspension of medication.<br>Recommend treatment with corticosteroids.	• Closely monitor changes in the subject's symptoms and signs.<br>• If treated with corticosteroids, follow-up refers to grade 3.
**3**	Symptoms occur at rest or with mild activity or exertion; treatment required.	Suspension of medication.<br>1 to 2 mg/kg/day of prednisone or other equivalent corticosteroids intravenously.	• Closely monitor changes in the subject's symptoms and signs.<br>• Tapering of corticosteroid dosage if symptoms improve.
**4**	Life-threatening, requiring urgent treatment (e.g., continuous fluid therapy or mechanically assisted circulation)	Permanent termination of medication.<br>1 to 2 mg/kg/day of prednisone or other equivalent corticosteroids intravenously.	• Tapering of corticosteroid dosage if symptoms improve.<br>• Consider prophylactic use of antibiotics against opportunistic infections.

(3) The occurrence of skin-related adverse events to Camrelizumab and management recommendations:

- In subjects receiving Camrelizumab monotherapy: the incidence of immune-related skin reactions including rash, pruritus, dermatitis, skin pigmentation abnormalities, and alopecia was 9.6%, and the incidence of immune-related skin reactions of reactive cutaneous capillary endothelial proliferation (RCCEP) was 74.1%. The severity of most cases was grade 1 or 2 with an incidence of 9.6% and grade 3 with an incidence of 0.4%, respectively, and there were no reports of grade 4 or higher immune-related adverse reactions. The median time to occurrence of immune-related skin adverse reactions was 0.8 months (0-11.2) and the median time to occurrence of RCCEP was 0.9 months (0-6.7).
- **Reactive capillary endothelial proliferation** (RCEP) occurs predominantly in the skin, and RCEP that occurs in the skin is RCCEP. The majority of subjects (**73.1%**, 106/145) developed RCCEP during the first cycle of treatment (2-4 w). All RCCEP was grade 1-2, with more than 80% grade 1. 55.2% (80/145) of subjects had improvement or resolution of RCCEP that was reversible. Combination of VEGF inhibitors (Apatinib, etc.) reduced the incidence of RCCEP to 5.4% while significantly improving efficacy. On the basis of clinical observation and expert opinion, and combining the CSCO guideline on the management of skin-related adverse reactions caused by immunotherapy, the grading and treatment of RCCEP are recommended as follows:

RCEP	Presentation	Treatment Recommendations
Grade 1	Multiple or single nodules ≤ 10 mm in maximum diameter with or without ulceration and bleeding	Continue medication, strengthen local treatment to prevent infection in patients with ulceration and bleeding
Grade 2	Multiple or single nodules ≥ 10 mm in maximum diameter with or without ulceration and bleeding	Continue medication, observe or take local treatment measures such as laser or surgical resection, etc., strengthen local treatment to prevent infection in patients with ulceration and bleeding
Grade 3	Generalized multiple nodules throughout the body, with concurrent skin infection	Suspend the drug, resume dosing after recovery to ≤ Grade 1, observe or take local treatment measures such as laser or surgical resection, etc., and give anti-infective therapy for patients with concurrent infection
Note: To date, there have been no Grade 4 life-threatening and Grade 5 fatal adverse events, so no classification was specified.		

In general principle, depending on the severity of the adverse reaction, the approach is to suspend treatment with Camrelizumab and consider reintroducing Camrelizumab when the severity of the AE returns to grade 1 or lower, and to

permanently discontinue Camrelizumab when a serious grade 3 or life-threatening grade 4 adverse reaction happens. Handling of immune-related adverse reactions should be in accordance with the medical practices and guidelines of the research institution. Adverse events associated with Camrelizumab (SHR-1210) may be immune-related (irAE) and may occur within a relatively short period of time after the first dose or several months after the last dose. Camrelizumab administration should be suspended if any of the conditions listed below occur. Common immune-related adverse reactions to Camrelizumab and their dose-adjustment criteria are listed in Table 2. A decision may be made on the basis of clinical practice if the investigator believes that Camrelizumab must be suspended or resumed if the investigator is unable to proceed as outlined in the table below or encounters a situation that is not outlined in the table considering the benefits/risks ratio to the subject during clinical practice.

Table 2: Dose modifications of Camrelizumab due to immune-related toxicities

**Camrelizumab-related Toxicities**	**Grade of Treatment Suspended (NCI CTC v5.0)**	**Time for Restarting Treatment**	**Permanent discontinuation**
Diarrhoea/Colitis	Grade 2-3	Toxicity is reduced to Grade 0-1.	Toxicity does not resolve within 4-6 weeks after the last dose, or the dose of corticosteroids cannot be reduced to 10 mg/day or lower dose of Prednisone (or equivalent) within 12 weeks.
	Grade 4	Permanent discontinuation.	Permanent discontinuation.
AST, ALT or bilirubin increased	Grade 2	Toxicity is reduced to Grade 0-1.	Toxicity does not resolve within 4-6 weeks after the last dose, or the dose of corticosteroids cannot be reduced to 10 mg/day or lower dose of Prednisone (or equivalent) within 12 weeks.
	Grade 3-4	Permanent discontinuation (Please refer to remark a below).	Permanent discontinuation.

Type I diabetes mellitus (if new) or hyperglycemia with evidence of pancreatic β-cell failure	Grade 3-4 or new type I diabetes mellitus	Treatment with SHR-1210 may not be restarted until the subject's clinical and metabolic status has stabilized.	
Hyperthyroidism	Grade 3	Toxicity is reduced to Grade 0-1.	Toxicity does not resolve within 4-6 weeks after the last dose, or the dose of corticosteroids cannot be reduced to 10 mg/day or lower dose of Prednisone (or equivalent) within 12 weeks.
	Grade 4	Permanent discontinuation.	Permanent discontinuation.
Thyroid function decreased	Grade 2-4	Treatment may be continued after initiation of thyroid hormone replacement therapy.	Treatment may be continued after initiation of thyroid hormone replacement therapy.
Pneumonitis	Grade 2	Toxicity is reduced to Grade 0-1.	Toxicity does not resolve within 4-6 weeks after the last dose, or the dose of corticosteroids cannot be reduced to 10 mg/day or lower dose of Prednisone (or equivalent) within 12 weeks.
	Grade 3-4	Permanent discontinuation.	Permanent discontinuation.
Immune-related hypophysitis	Grade 2-4	Toxicity is reduced to Grade 0-1; treatment may be continued after initiation of endocrine replacement therapy.	Toxicity does not resolve within 4-6 weeks after the last dose, or the dose of corticosteroids cannot be reduced to 10 mg/day or lower dose of Prednisone (or equivalent) within 12 weeks.
Infusion reactions	Grade 2 (please refer to remark b below)	Toxicity is reduced to Grade 0-1.	If symptoms recur after adequate administration of prophylactic medication, the drug should be permanently discontinued.
	Grade 3-4	Permanent discontinuation.	Permanent discontinuation.

Renal failure or nephritis	Grade 2	Toxicity is reduced to Grade 0-1.	Toxicity does not resolve within 4-6 weeks after the last dose, or the dose of corticosteroids cannot be reduced to 10 mg/day or lower dose of Prednisone (or equivalent) within 12 weeks.
	Grade 3-4	Permanent discontinuation.	Permanent discontinuation.
Other drug-related toxicities<br>(Please refer to remarks below)	Grade 3	Toxicity is reduced to Grade 0-1.	Toxicity does not resolve within 4-6 weeks after the last dose, or the dose of corticosteroids cannot be reduced to 10 mg/day or lower dose of Prednisone (or equivalent) within 4-6 weeks.
	Grade 4	Permanent discontinuation.	Permanent discontinuation.

Note: if any serious or ≥ Grade 3 (pneumonitis ≥ Grade 2) drug-related AE or any life-threatening AE reoccurs, the drug should be permanently discontinued.

a. For subjects with Grade 2 AST or ALT increased at baseline, the drug should be permanently discontinued if AST or ALT increases ≥ 50% from baseline during treatment and lasts for at least 1 week.

b. If symptoms resolve within 1 hour of temporary discontinuation, the infusion may be continued at 50% of the initial infusion rate. Otherwise, the drug must be restarted after the symptoms are completely resolved. Adequate prophylactic medication should also be administered to the subject at the next dose.

c. Camrelizumab may be suspended at the investigator's discretion for subjects with intolerable or persistent Grade 2 or higher drug-related AEs.

(4) Dose adjustment for platinum-doublet chemotherapy:

At the start of the next cycle, dose adjustments are required based on the lowest blood cell counts and most severe non-hematologic toxicities of previous cycles. Treatment may be delayed to allow adequate recovery time. Upon recovery, subjects should be re-treated according to the guidelines in Tables 3-5.

Table 3. Dose adjustments for pemetrexed due to toxicity

Toxicity associated with Pemetrexed (definition or refer to NCI CTC v5.0)	Restarting treatment standards	Restarting treatment dosages	Permanent discontinuation
**Hematologic toxicity**			
Absolute neutrophil minimum <500/mm^3 and platelet nadir ≥50,000/mm^3	The next cycle of therapy can only be initiated if the absolute neutrophil minimum is >1500/mm^3	75% of original dose	Treatment was discontinued if toxicity did not resolve within 9

Platelet nadir <50,000/mm^3. Regardless of the absolute neutrophil minimum	and the platelet minimum is ≥100,000/mm^3	75% of original dose	weeks of the last dose or recurred after experiencing 2 dose reductions.
Platelet nadir <50,000/mm^3 with bleeding[a]. Regardless of the absolute neutrophil minimum		50% of original dose	
**Non-hematological toxicity**[b]			
Diarrhea requiring hospitalization (regardless of grade) or grade 3/4 diarrhea (after adequate antidiarrheal therapy)	Toxicity reduced to level 0-1	75% of original dose	Treatment was discontinued if toxicity did not resolve within 9 weeks of the last dose or recurred after experiencing 2 dose reductions.
Other non-hematologic toxicity of grade 3/4	Nephrotoxicity: creatinine clearance ≥60 mL/min; Hepatotoxicity: total bilirubin ≤ 1.5 times the upper limit of normal, ALP, AST and ALT ≤ 3 times the upper limit of normal (ALP, AST and ALT ≤ 5 times the upper limit of normal if the tumor involves the liver); Other non-hematologic toxicity: reduction to grade 0-1 or return to baseline period levels; Subjects will only be able to start the next cycle of treatment.	75% of original dose	
**Neurotoxicity**			
Level 2	Toxicity reduced to level 0-1	75% of original dose	Treatment was discontinued if toxicity did not resolve within 9 weeks of the last dose or recurred after experiencing 2 dose reductions.
Level 3-4	Toxicity reduced to level 0-1	50% of original dose or permanent termination	

Table 4: Dose adjustments for carboplatin due to toxicity

Toxicity associated with Carboplatin (definition or refer to NCI CTC v5.0)	Restarting treatment standards	Restarting treatment dosages	Permanent withdrawal
**Hematologic toxicity**			
Absolute neutrophil minimum <500/mm^3 and platelet nadir ≥50,000/mm^3	The next cycle of therapy can only be initiated if the absolute neutrophil minimum is >1500/mm^3 and	75% of original dose	Treatment was discontinued if toxicity did not resolve within

Platelet nadir <50,000/mm^3. Regardless of the absolute neutrophil minimum	the platelet minimum is ≥100,000/mm^3	75% of original dose	9 weeks of the last dose or recurred after experiencing 2 dose reductions.
Platelet nadir <50,000/mm^3 with bleeding. Regardless of the absolute neutrophil minimum		50% of original dose	
Absolute neutrophil nadir< 1000/mm^3 with fever≥ 38.5°C		50% of original dose	
**Non-hematological toxicity**			
Diarrhea requiring hospitalization (regardless of grade) or grade 3/4 diarrhea (after adequate antidiarrheal therapy)	Toxicity reduced to level 0-1	75% of original dose	Treatment was discontinued if toxicity did not resolve within 9 weeks of the last dose or recurred after experiencing 2 dose reductions.
Grade 3 or 4 nausea/vomiting (with or without anti-nausea/vomiting treatment)	Toxicity reduced to level 0-1	75% of original dose	
Other non-hematologic toxicity of grade 3/4	Nephrotoxicity: creatinine clearance ≥60 mL/min; Hepatotoxicity: total bilirubin ≤ 1.5 times the upper limit of normal, ALP, AST and ALT ≤ 3 times the upper limit of normal (ALP, AST and ALT ≤ 5 times the upper limit of normal if the tumor involves the liver); Other non-hematologic toxicity: reduction to grade 0-1 or return to baseline period levels; Subjects will only be able to start the next cycle of treatment.	75% of original dose	
**Neurotoxicity**			
Level 2	Toxicity reduced to level 0-1	75% of original dose	Treatment was discontinued if toxicity did not resolve within 9 weeks of the last dose or recurred after experiencing 2 dose reductions.
Level 3-4	Toxicity reduced to level 0-1	50% of original dose or permanent termination	

Table 5. Dose adjustments for gemcitabine due to toxicity

Absolute neutrophil count (×10^6/L)		Platelet count (×106/L)	% of total dose
>1,000	and	>100,000	100

500~1,000	or	50,000~100,000	75
<500	or	<50,000	discontinuation

(5) Apatinib dose adjustment:

The dose of Apatinib was 250 mg, with dose pauses and dose adjustments allowed (one day on and one day off). The cumulative duration of dosing pauses must not exceed 1 week per dosing cycle to ensure that subjects in the trial receive the drug efficacy of the treatment.

Table 6 Principles of Dose Adjustment for Apatinib

**Classification of adverse reactions**	**NCI degree**	**Provisions for dose adjustments**
Hematologic Adverse Reactions	Level 1 to 2	Maintain original dose level;
	First 3~4 levels	Suspend the drug, wait for the adverse reactions to return to ≤ grade 1, and maintain the original dose to continue the drug;
	Second 3 to 4 levels	Discontinue medication;
Non-hematologic adverse reactions	Level 1-2	Maintain original dose level;
	First 3~4 levels	Suspend the drug, wait for the adverse reactions to return to ≤ grade 1, and maintain the original dose to continue the drug;
	Second 3 to 4 levels	Discontinue medication.

The measures that should be taken with Apatinib in the event of the following special circumstances are described below, and a specialist consultation with the relevant department should be requested if necessary:

Hemorrhage

• A grade 3 or 4 bleeding event should be treated accordingly and Apatinib therapy should be permanently discontinued

Thrombosis/Embolism

• Apatinib treatment should be permanently discontinued for any grade of arterial thrombotic event

• Grade 4 venous thrombosis, treatment with Apatinib should be permanently discontinued

• Grade 3 venous thrombosis, Apatinib therapy should be suspended. If the planned therapeutic dose of anticoagulation therapy is ≤ 2 weeks, Apatinib therapy should be suspended until anticoagulation therapy is completed. If the planned therapeutic dose of anticoagulation therapy for > 2 weeks, Apatinib therapy should be suspended for 2

weeks until the following criteria are met to restart study medication in the period of anticoagulation therapy:

- Before restarting study drug therapy, the INR ratio should be within the target range (typically 2 ~ 3).

- Subjects must not have a Grade 3 or Grade 4 bleeding event since entering the study.

- Subjects with no evidence of tumor invasion or adjacency to large vessels on prior tumor evaluation.

Note: Therapeutic-dose anticoagulation is defined as a dose of Warfarin or other anticoagulant medication that is increased incrementally from a small dose to a dose level that maintains an INR of no less than 1.5 (usually between 2 and 3). The dose of warfarin should be recorded in the eCRF and the INR should be monitored throughout the treatment period in subjects receiving anticoagulation.

High Blood Pressure

Subjects should have frequent blood pressure measurements in order to monitor the emergence and worsening of hypertension.

• Grade 1: Pre-hypertension (systolic blood pressure 120-139 mmHg, diastolic blood pressure 80-89 mmHg) without intervention.

• Grade 2: Stage 1 hypertension (systolic 140-159 mmHg, diastolic 90-99 mmHg) requiring medical intervention, recurrent or persistent (≥ 24 hours), symptomatic increase in systolic blood pressure of > 20 mmHg or previously in the normal range to > 140/90 mmHg. Apatinib therapy should be suspended. An antihypertensive drug is allowed to use. Once blood pressure is controlled to < 140/90 mmHg, subjects may continue to receive Apatinib treatment.

• Grade 3: Stage 2 hypertension (systolic blood pressure ≥ 160 mmHg, diastolic blood pressure ≥ 100 mmHg) requiring more than one antihypertensive medication or more intense treatment than previously. For persistent or symptomatic hypertension, Apatinib treatment should be suspended; if hypertension remains uncontrolled after 4 weeks of treatment, Apatinib treatment should be permanently discontinued.

• Grade 4: Life-threatening (malignant hypertension or persistent nerve damage, hypertensive crisis) Acute intervention, life-threatening (e.g., hypertensive crisis). If Grade 4 hypertension occurs, treatment with Apatinib should be permanently discontinued. The dose of antihypertensive drug used should be recorded at each visit.

Proteinuria

Perform routine urinalysis as per the study flowchart if:

• < 2+, continue Apatinib dosing as planned, no additional testing required.
• ≥ 2+, monitor 24-hour urine protein immediately. 24-hour urine protein:
  - < 1g: continue Apatinib treatment as planned;
  - 1 to 3.4 g, suspend Apatinib administration until 24-hour urinary protein < 1 g, then continue Apatinib therapy. If second occurrence, suspend Apatinib administration;
  - ≥3.5 g or development of nephrotic syndrome, then permanently discontinue Apatinib.

3.4.3 Combined treatment and smoking

All co-medications and treatments (including start/stop dates and indications) must be documented in the subject's original profile and in the appropriate section of the electronic case report form (eCRF). It is recommended that all subjects quit smoking during the course of treatment.

3.4.4 Concomitant medication

Drugs prohibited or used with caution during study drug treatment:

(1) Apatinib is mainly metabolized by the hepatic enzyme CYP3A4, and subjects using Apatinib should be cautious of using CYP3A4 inducers (Dexamethasone, Ketamine, Rifampicin, and Phenobarbital) and inhibitors (Ketoconazole, Itraconazole, Erythromycin, and Kratom), CYP3A4 substrates (Simvastatin, Cyclosporine, and Pimozide), other drugs metabolized by CYP3A4 (e.g., Benzodiazepines, Dihydropyridine, calcium antagonists, and HMG-COA reductase inhibitors), and CYP2C19 substrates (Diazepam, Promethazine, Lansoprazole).

(2) Due to the clinical toxicity of prolonging the QT interval of Apatinib-based drugs, subjects using Apatinib should use drugs that prolong the QT interval with caution during treatment. This includes, but is not limited to, the following classes of drugs: antimicrobials (Clarithromycin, Azithromycin, Erythromycin, Roxithromycin, Metronidazole, Moxifloxacin); antiarrhythmics (Quinidine, Sotalol, Amiodarone, Propyzamide, Procainamide); antipsychotics (Risperidone, Fluphenazine, Haloperidol, Haloperidol, Thioridazine, Pimozide, Olanzapine, Clozapine); anti-fungal drugs (Fluconazole, Ketoconazole); antimalarials (Mefloquine, Chloroquine); antidepressants (Amitriptyline, Promethazine, Clomipramine, Dutasteride, Doxepin).

(3) Subjects in both groups are prohibited from using NMPA-approved targeted agents for lung cancer, modern Chinese herbal preparations, and immunomodulators (e.g.,

Thymosin, Interferon, Interleukin-2, Zilongjin, and Lentinan) during the period of treatment with the study drug.

3.4.5 Treatment compliance

The drug, dose, and time of administration of Camrelizumab, Apatinib, and platinum-containing dual-agent chemotherapy administered for each course of treatment for each subject should be recorded on the eCRF form. Reasons for delayed administration, drug reduction, or missed doses should also be documented in the eCRF.

Subject adherence to treatment and regimen includes voluntary compliance with all aspects of the regimen. Subjects who do not return for visits or medication may be terminated at the discretion of the principal investigator.

3.5 Research indicators

Major pathologic response (MPR) Rate: the ratio of subjects experiencing major pathologic response to the total number of subjects undergoing surgery. Major pathologic response was defined as no more than 10% of residual viable tumor cells in the tumor bed in the postoperative specimen.

Pathological complete response (pCR) rate: the ratio of subjects who experienced pathological complete response to those who underwent surgery. Complete pathological response was defined as no residual viable tumor cells in the lung and lymph nodes (assessed by pathological review).

Objective response rate (ORR): the rate of subjects whose tumors shrank by a certain amount and remained a certain period of time, including complete response (CR) and partial response (PR) cases. Objective tumor response was assessed using the Response Evaluation Criteria in Solid Tumor (RECIST 1.1 criteria). The efficacy assessment criteria were classified as CR, PR, stable disease (SD), and progressive disease (PD) according to the RECIST 1.1 criteria.

Event-free survival (EFS): defined as the time from the start of the first dose to the occurrence of any of the following events. Events include: disease progression leading to inoperable disease, disease progression after surgery, or death from any cause, whichever occurs earliest.

Disease-free survival (DFS): defined as the time from the end of surgery to disease recurrence or death (due to any cause), 1- and 2-year DFS rates were counted.

Overall Survival (OS): defined as the time from random enrollment to death of participant due to any cause. Subjects who are alive at the time of analysis will have the date of their last contact as the cut-off date.

Safety: observe any adverse events, including abnormal clinical symptoms and vital signs, abnormalities in laboratory tests, occurring in all subjects from the time of signing the informed consent form up to 90 days after the last dose of Camrelizumab or 30 days after the last dose of Apatinib (whichever is longer), record the characteristics of the clinical manifestations, severity, time of onset, duration, management, and prognosis, and determine the correlation between them and the test drug. The safety of the study drug will be evaluated using the National Cancer Institute Common Toxicity Criteria (NCI-CTCAE) version 5.0 criteria.

R0 resection rate: defined as the proportion of patients with margin-negative resection.

Quality of life score: refer to the questionnaire QOL-LC-13 of Shanghai Pulmonary Hospital.

Exploratory metrics: seeking for potentially sensitive markers for immunotherapy, including but not limited to tumor-infiltrating lymphocytes and PD-L1 expression.

3.6 Research process

3.6.1 Screening phase

It should be completed in -D28 - D0 days:

- Sign the informed consent form.
- Collect demographic information: gender, year of birth, height, weight, etc.
- Pathologic diagnosis: mainly by puncture biopsy or bronchoscopy, the record includes the date of diagnosis, histologic typing, part of the lesion (primary or metastatic), TNM stage, and clinical stage;
- Genetic testing (EGFR & ALK).
- PD-L1 expression (Arm B only).
- Past medical history, family history, allergy history.
- Smoking history: duration of smoking, average amount smoked.
- Electrocardiogram: 12-lead electrocardiogram.
- Echocardiography: LVEF, etc.

- Pulmonary function: FEV1, FVC, FEV1/FVC, FEV1%.
- Hepatitis Tests: Hepatitis B Tests (Hepatitis B surface antigen, Hepatitis B surface antibody, Hepatitis B e antigen, Hepatitis B e antibody, Hepatitis B core antibody, Hepatitis B core antibody IgM measurement); HCV Antibody Tests.
- HIV antibody test.
- Thyroid function: free triiodothyronine (FT3), free thyroxine (FT4), thyroid stimulating hormone (TSH), triiodothyronine (T3) and thyroxine (T4).
- Concomitant medications and adverse events.
- Venous blood 10 ml (for research).
- Quality of life scores.
- Imaging.

It should be completed in D-7 - D0 days:

- Checks on criteria for admission to the platoon.
- Vital signs: heart rate, respiratory rate, temperature, blood pressure.
- Physical examination: including skin mucosa, lymph nodes, head and neck, chest, abdomen, musculoskeletal, nervous system, respiratory system, cardiovascular system, and urinary system.
- ECOG PS Rating.
- Routine blood counts: hemoglobin, red blood cell count, white blood cell count, neutrophil count, lymphocyte count, and platelet count.
- Blood biochemistry: lactate dehydrogenase (LDH), alkaline phosphatase (ALP), total cholesterol (TC), triglycerides (TG), total protein (TP), albumin (ALB), fasting glucose (GLU), alanine aminotransferase (ALT), glutamate aminotransferase (AST), gamma-glutamyl transpeptidase (GTP), total bilirubin (TBIL), direct bilirubin (DBIL), creatinine (Cr), urea nitrogen (BUN), potassium (K+), sodium (Na+), and chloride (Cl-);
- Cardiac enzyme profile: creatine kinase (CK)|, creatine kinase isoenzyme (CK-MB), myoglobin (Mb), troponin (cTnI).
- Pituitary-adrenal axis tests: these include hypothalamic corticotropin-releasing hormone (CRH), pituitary corticotropin (plasma ACTH), and adrenocorticotropic hormone assays. Adrenocorticotropic hormone measurements include serum cortisol, urinary free cortisol (UFC), urinary 17-ketosteroids (17-KS) and urinary 17-ketogenic steroids (17-KGS). (This test or some of these indicators may be performed in hospitals where available).

- Stool routine: fecal occult blood OB.
- Coagulation tests: PT, APTT, INR, TT, FDP.
- Urine routine: urine protein (PRO), urine glucose (GLU), urine occult blood (urine red blood cells, urine white blood cells).
- Pregnancy test (women of childbearing age).

3.6.2 Preoperative treatment period

All enrolled subjects received 2-4 cycles of combination drug therapy:

(1) Arm A: Camrelizumab + Platinum-doublet chemotherapy

Camrelizumab: a fixed dose of 200 mg, intravenous drip over a period of 30-60 min, d1, every 3 weeks as a cycle, preoperative use of 2-4 cycles;

Platinum-containing two-drug chemotherapy: ① Squamous carcinoma: Carboplatin AUC5 d1 + Gemcitabine 1000mg/m2 , d1, d8 (or Paclitaxel 135~175 mg/m2 , d1; or Docetaxel 60~75 mg/m2 , d1; or Albumin Paclitaxel 260 mg/m², d1) ② Non-squamous carcinoma: Carboplatin AUC5 d1, Pemetrexed 500mg/m2, d1), 3 weeks as a treatment cycle, and 2-4 cycles of preoperative administration.

(2) Arm B: Camrelizumab + Apatinib

Camrelizumab: a fixed dose of 200 mg, intravenous drip over a period of 30-60 min, d1, every 3 weeks as a cycle, preoperative use of 2-4 cycles;

Apatinib: 250 mg/day, orally once daily for 3 weeks as a treatment cycle, 2-4 cycles of preoperative administration.

Prior to the start of drug therapy in Cycles 2, 3, and 4 (C2D1±3, and C3D1±10 and C4D1±10 [if required]), all subjects will be required to undergo the following examinations:

- Vital signs: heart rate, respiratory rate, temperature, blood pressure.
- Physical examination: including skin mucosa, lymph nodes, head and neck, chest, abdomen, musculoskeletal, nervous system, respiratory system, cardiovascular system, and urinary system.
- ECOG PS Rating.
- Routine blood counts: hemoglobin, red blood cell count, white blood cell count, neutrophil count, lymphocyte count, and platelet count.
- Blood biochemistry: lactate dehydrogenase (LDH), alkaline phosphatase (ALP), total cholesterol (TC), triglycerides (TG), total protein (TP), albumin (ALB), fasting glucose (GLU), alanine aminotransferase (ALT), glutamate

aminotransferase (AST), gamma-glutamyl transpeptidase (GTP), total bilirubin (TBIL), direct bilirubin (DBIL), creatinine (Cr), urea nitrogen (BUN), potassium (K+), sodium (Na+), and chloride (Cl-);

- Coagulation tests: PT, APTT, INR, TT, FDP (performed only on C3D1±10d).
- Urine routine: urine protein (PRO), urine glucose (GLU), urine occult blood (urine red blood cells, urine white blood cells).
- Stool routine: fecal occult blood OB (only C3D1±10 was examined);
- Thyroid function: free triiodothyronine (FT3), free thyroxine (FT4), thyroid stimulating hormone (TSH), and triiodothyronine (T3) and thyroxine (T4);
- Pituitary-adrenal axis tests: these include hypothalamic corticotropin-releasing hormone (CRH), pituitary corticotropin (plasma ACTH), and adrenocorticotropic hormone assays. Adrenocorticotropic hormone measurements include serum cortisol, urinary free cortisol (UFC), urinary 17-ketosteroids (17-KS), and urinary 17-ketogenic steroids (17-KGS) (C3D1 ±10 only). (This test or some of these indicators can be performed in hospitals where available).
- Electrocardiogram: 12-lead electrocardiogram.
- Quality of life scores.
- Venous blood 10 ml (for research).
- Concomitant medications and adverse events.
- Imaging (no imaging prior to starting medication in cycle 2)

3.6.3 Preoperative assessment:

- Vital signs: heart rate, respiratory rate, temperature, blood pressure.
- Physical examination: including skin mucosa, lymph nodes, head and neck, chest, abdomen, musculoskeletal, nervous system, respiratory system, cardiovascular system, and urinary system.
- ECOG PS Rating.
- Routine blood counts: hemoglobin, red blood cell count, white blood cell count, neutrophil count, lymphocyte count, and platelet count.
- Blood biochemistry: lactate dehydrogenase (LDH), alkaline phosphatase (ALP), total cholesterol (TC), triglycerides (TG), total protein (TP), albumin (ALB), fasting glucose (GLU), alanine aminotransferase (ALT), glutamate aminotransferase (AST), gamma-glutamyl transpeptidase (GTP), total bilirubin

(TBIL), direct bilirubin (DBIL), creatinine (Cr), urea nitrogen (BUN), potassium (K+), sodium (Na+), and chloride (Cl-);

- Cardiac enzyme profile: creatine kinase (CK)|, creatine kinase isoenzyme (CK-MB), myoglobin (Mb), troponin (cTnI).
- Electrocardiogram: 12-lead electrocardiogram.
- Cardiac ultrasound: LVEF, etc.
- Lung function: FEV1, FVC, FEV1/FVC, FEV1%.
- Urine routine: urine protein (PRO), urine glucose (GLU), urine occult blood (urine red blood cells, urine white blood cells).
- Stool routine: fecal occult blood;
- Thyroid function: free triiodothyronine (FT3), free thyroxine (FT4), thyroid stimulating hormone (TSH), and triiodothyronine (T3) and thyroxine (T4);
- Pituitary-adrenal axis tests: these include hypothalamic corticotropin-releasing hormone (CRH), pituitary corticotropin (plasma ACTH), and adrenocorticotropic hormone assays. Adrenocorticotropic hormone measurements include serum cortisol, urinary free cortisol (UFC), urinary 17-ketosteroids (17-KS), and urinary 17-ketogenic steroids (17-KGS) (this test, or some of them, may be performed in hospitals where available).
- Coagulation tests: PT, INR, APTT, TT, FDP.
- Hepatitis indicators: Hepatitis B testing (Hepatitis B surface antigen, Hepatitis B surface antibody, Hepatitis B e antigen, Hepatitis B e antibody, Hepatitis B core antibody, Hepatitis B core antibody IgM assay); HCV antibody testing.
- Venous blood 10 ml (for research).
- Concomitant medications and adverse events.
- Imaging.

3.6.4 Surgical period

Within 30 days of preoperative evaluation, subjects in both groups underwent radical surgery.

3.6.5 Postoperative assessment

The following evaluations should be completed 30 days ± 10 days after surgery:

- Vital signs: heart rate, respiratory rate, temperature, blood pressure.

- Physical examination: including skin mucosa, lymph nodes, head and neck, chest, abdomen, musculoskeletal, nervous system, respiratory system, cardiovascular system, and urinary system.
- ECOG PS Rating.
- Routine blood counts: hemoglobin, red blood cell count, white blood cell count, neutrophil count, lymphocyte count, and platelet count.
- Blood biochemistry: lactate dehydrogenase (LDH), alkaline phosphatase (ALP), total cholesterol (TC), triglycerides (TG), total protein (TP), albumin (ALB), fasting glucose (GLU). Albumin transaminase (ALT), albumin transaminase (AST), gamma-glutamyl transpeptidase (GTP), total bilirubin (TBIL), direct bilirubin (DBIL), creatinine (Cr), urea nitrogen (BUN), potassium (K+), sodium (Na+), chloride (Cl-).
- Cardiac enzyme profile: creatine kinase (CK)|, creatine kinase isoenzyme (CK-MB), myoglobin (Mb), troponin (cTnI).
- Electrocardiogram: 12-lead electrocardiogram.
- Coagulation tests: PT, INR, APTT, TT, FDP.
- Urine routine: urine protein (PRO), urine glucose (GLU), urine occult blood (urine red blood cells, urine white blood cells).
- Thyroid function: free triiodothyronine (FT3), free thyroxine (FT4), thyroid stimulating hormone (TSH) and triiodothyronine (T3) and thyroxine (T4).
- Quality of life scores.
- Concomitant medications and adverse events.
- Pregnancy test (women of childbearing age).
- Imaging.

3.6.6 Postoperative adjuvant therapy period (observational study)

The investigators decide whether to carry out adjuvant chemoradiotherapy at 4-8 weeks postoperatively according to the subject's postoperative status and pathological results, or according to the response to neoadjuvant regimen (for those who have responded well to neoadjuvant therapy). Under the recommendations of the doctor and the subject's willingness, the adjuvant treatment scheme is conducted in trial hospital and/or outside hospitals.

3.6.7 Postoperative follow-up period

Postoperative visit is conducted every 3 months ± 14 days for first 2 years, and every 6 months ± 14 days for years 3-5, including routine examinations, CT scan of the chest, systemic ECT in case of bone pain, and cranial MRI if brain metastasis is suspected in the judgment of the investigator. Subjects with recurrence of disease within 5 years or without recurrence at the end of the study (5 years after surgery) will be contacted (by visit or by phone) every 6 months ± 14 days to obtain information about their survival and subsequent treatment after the end of the study. Follow-up includes survival status, time to relapse, date and cause of death if death occurs, and other antitumor therapy received after progression.

3.6.8 Unplanned visits

Unscheduled visits should be conducted according to clinical need, and corresponding clinically significant laboratory test abnormalities and adverse events should be documented in the eCRF and in the source material. If multiple laboratory tests are performed on the same day, only the most recent set of values from those tests should be recorded in the eCRF. However, all abnormal values from repeated laboratory tests should be recorded in the eCRF.

3.6.9 Follow-up of subjects with disease recurrence or who have not relapsed at the end of the study (after 5 years postoperatively)

Subjects will be contacted (by visit or by telephone) every 6 months ± 14 days to obtain information including overall survival and subsequent treatment at the end of the study until the death of the subject. The following information should be obtained during each follow-up visit:

- Survival of subjects.
- If deceased record the date of death and cause of death in detail.
- Disease status and whether it has recurred, and if it has recurred, details of the date of recurrence (for subjects who have not had a recurrence at the last follow-up visit).
- Detailed records of subsequent anticancer treatment (including reoperation status, chemoradiotherapy and immunotherapy after recurrence) after recurrence.

Note: Subjects who have developed tumor recurrence will be followed up according to local medical practice at the discretion of the clinician.

3.7 Quality of data

In order to follow the guidelines of the Good Clinical Practice (GCP), the supervisors will visit the center on a regular basis to ensure compliance with the study protocol, GCP and relevant laws. The visits will include on-site checking of the completeness and clarity of the electronic case report form (eCRF), cross-checking with the original documents, and clarification of administrative matters.

3.8 Archives

The information entered into the electronic case report form (eCRF) must match the original document, and in the case of direct recording into the eCRF, what is documented will be treated as the original source. The parameters of the source material must be validated, and information about the source must be documented. The study documentation and all source material should be retained until notification of destruction is received from the sponsor.

4. Ethical and legal aspects

4.1 Independent Ethics Committee (IEC) or Institutional Review Board (IRB)

In accordance with GCP, our laws and regulations, and the requirements of relevant organizations, all participating centers should obtain approval documents from the appropriate ethics committee/institutional review board before the study begins. If necessary, extension, amendment or re-examination of the ethics committee approval document must be obtained and forwarded to the investigator.

4.2 Ethical guidance for this study

The procedures involved in this study protocol regarding manipulation, evaluation, and preparation of documentation are designed to ensure that the investigators follow the guidelines of the clinical practice guidelines as well as the guiding principles detailed in the Declaration of Helsinki. The implementation of this study will also follow the appropriate laws and regulations of our country.

None of the researchers can make changes in the study protocol without consent. However, in emergency situations to remove risk factors to subjects, the investigator

may deviate from or change the study protocol without the consent/support of the ethics committee/institutional review board/sponsor. The deviation or change made and the reasons for it should be submitted to the Ethics Committee/Institutional Review Board/Sponsor as soon as possible and, if appropriate, a proposal for protocol modification should be submitted. All deviations or changes to the study protocol must be fully explained and justified by the investigator.

4.3 Information for Subjects and Informed Consent

Subjects should be provided with key information about the study and the informed consent form. Prior to the start of the study, the investigator must provide the subjects with the written ethics committee/institutional review board approval/favorable opinion of the informed consent form and all other written information. The Ethics Committee/Institutional Review Board approval and the approved Instructions to Subjects/Informed Consent Form must be filed together in the study file.

Informed consent must be obtained prior to performing any specific research step. The date of the subject's participation in the study and the signing of the informed consent should be recorded in the subject's appropriate documentation.

4.4 Confidentiality

All records relating to the identity of subjects are kept confidential and, to the extent permitted by relevant laws and/or regulations, such information will not be made available to the public.

Subjects' names will not be provided. Only the subject's number and initials will be recorded on the case report form. If the subject's name appears in any other document (e.g., pathology report), it must be blacked out in the copy of the document that will be made. Studies using computerized storage must comply with local laws regarding data protection. The identity of the subjects will also be kept confidential when the results of the study are published.

A list will be maintained by the investigator for the purpose of identifying subjects' records.

4.5 Maintenance of study documents, medical record report forms and records

4.5.1 Researcher folder/file saving

In order to ensure that the conduct of the study is fully documented and that the study data can subsequently be validated, it is essential that the investigator maintains comprehensive and accurate records of the conduct of the study, and these documents should be categorized into two distinct categories: (1) the investigator's study documents, and (2) the original clinical records of the subjects.

The investigator's study documents included trial protocols and revisions, letters from independent ethics committees/institutional review boards and government approval documents, sample informed consent forms, medication records, personnel biographies, authorizations, and other relevant documents/correspondence.

Original subject clinical records (usually pre-defined prior to the start of the project, documented outside of the chart report form, key efficacy/safety parameters), typically including subject inpatient/outpatient records, physician and nurse notes, appointment letters, original laboratory reports, ECGs, EEGs, x-rays, pathology and specialized evaluation reports, signed informed consent forms, counseling letters, subject screening and recruitment forms.

4.5.2 Audits and inspections

The investigator should be aware that, upon formal notification, the original records relating to the study should be prepared and made available to the appropriate qualified person from the Drug Study Quality Assurance Unit or his/her designee, or to an inspector from the health department. Reconciliation of data in the medical record report form must be based on direct inspection of the original records.

4.5.3 Electronic medical record report form

This study used an electronic data capture (EDC) system for the collection and management of clinical research data.

The data in the eCRF come from and should be consistent with the original documents, such as study charts and laboratory test reports. Any observation and examination results of the trial should be timely, correct, complete, standardized and truthful in the eCRF.

When making data corrections to the eCRF, follow the system prompts and fill in the reason for the data change. The investigator should complete, save and submit the eCRF in a timely manner at the end of this visit for each subject. The system logic verification program will check the completeness and logic of the data entered into the EDC system and issue challenges to problematic data, allowing the investigator or data entry personnel to modify or explain the problematic data, if necessary multiple times until the problematic data is resolved. Supervisors, data managers, and medical reviewers will also review eCRF data as necessary and issue challenges to questionable data. Investigators are expected to respond to queries from the system and from data reviewers in a timely manner. After data cleaning is complete, the PI electronically signs the completed eCRF.

5. Monitoring of research

If subject confidentiality meets local requirements, the responsible monitor (or designee) will contact and visit the investigator on a regular basis and will be permitted to inspect the various trial records (case report forms and other relevant data) as required.

The Supervisor is responsible for periodically reviewing the Case Report Form throughout the study period to verify compliance with the study protocol and to check the completeness, consistency, and accuracy of the data entered. The Supervisor shall be granted access to laboratory test reports and other subject records to verify inputs on the Case Report Form. The investigator (or his/her designee) agrees to cooperate with the monitor to ensure that any problems identified during these monitoring visits are resolved.

6. Statistical analysis plan

6.1 Sample size calculation

No inferential statistical tests are proposed in this study, sample size is determined by the pragmatics of recruitment, such as patient flow and budgetary constraints.

6.2 Analysis set

Full Analysis Set (FAS): it includes all enrolled subjects who have received at least one dose of study drug according to the intention-to-treat (ITT) principle.

Efficacy Assessable Analysis Set (EAS): it includes all subjects who have received at least one dose of study drug after enrollment and have undergone pathological assessment after surgical treatment.

Safety Analysis Set (SAS): it includes all enrolled subjects who have received at least one dose of study drug. The SS will be used for all safety analyses.

6.3 Summary of study data

Baseline demographic characteristics, baseline tumor characteristics, medical history, comorbid medications, vital signs, and termination of the trial will be summarized for all enrolled subjects. For category data, the number (N) and percentage (%) of subjects at each level of categorization, and the number and percentage of missing subjects will be provided. For continuous data, the number of evaluable subjects (N), mean (Mean) and standard deviation (Stand Deviation), median (Median), minimum (Min), maximum (Max) and other descriptive statistics will be provided.

6.4 Efficacy analysis

6.4.1 Main efficacy analysis

The primary endpoint of this study is MPR rate, the number of cases and percentage of subjects under each classification will be summarized and 95% confidence intervals for the rates will be calculated using the Clopper-Pearson method.

6.4.2 Secondary efficacy analysis

For secondary efficacy endpoints in the dichotomous categories (including pCR, ORR, R0 resection rate), the number of cases and percentage of subjects under each category will be summarized and 95% confidence intervals for the rates will be calculated using the Clopper-Pearson method.

For efficacy endpoints of the time-to-event type (including EFS, DFS, OS), the number and percentage of subject cases with events and censoring will be summarized, and summary statistics will be provided for the type of event and the reason for censoring. The censoring rules for time-to-event endpoints are detailed in the statistical analysis plan.

The median survival time and survival rate will be estimated using the Kaplan-Meier method and survival graphs will be plotted. The 95% confidence interval for

survival time will be calculated using the Brookmeyer-Crowley method and the 95% confidence interval for survival rate will be calculated using the log(-log) method.

6.5 Safety analysis

Treatment-emergent adverse events (TEAE)

TEAE for this study are defined as any adverse event that occurs or worsens after the first dose of study drug through 90 days after the last dose. All summary tables are based on TEAE; adverse events in screening-period will be provided as a list only.

Adverse events will be coded according to the MedDRA Dictionary and summarized by System Organ Classification (SOC) and Preferred Terminology (PT), relevance to the study drug, and CTCAE 5.0 severity grading.

Summary statistics will be provided for the following adverse event types:

- All TEAEs, all TEAEs related to investigational drugs;
- TEAE with CTCAE ≥ grade 3, TEAE with CTCAE ≥ grade 3 related to investigational drugs;
- Treatment-emergent serious adverse events (SAEs), SAEs related to investigational drugs;
- TEAE leading to permanent discontinuation, TEAE related to study drug leading to permanent discontinuation;
- TEAE leading to dose reductions, TEAE leading to dose reductions associated with study drugs;
- TEAE leading to medication suspension, TEAE leading to medication suspension related to investigational drugs;
- TEAE leading to withdrawal from the study, TEAEs related to the study drug that led to withdrawal from the study;
- TEAEs of special concern, TEAEs of special concern related to investigational drugs;
- TEAEs leading to death, TEAEs leading to death associated with study drugs.

The number and percentage of subjects experiencing each type of adverse event will be provided, and if a subject has multiple adverse events within the same SOC or PT, only the one with the highest severity will be counted. TEAEs related to the study drug include TEAEs that are definitely related to the study drug, possibly related, undeterminable, and for which correlation data are missing.

Laboratory tests, vital signs, electrocardiogram and physical examination

For continuous indicators, measurements and changes from baseline are summarized for each indicator at each program visit viewpoint. For categorical indicators, the number and percentage of subjects in each category will be provided for each program visit viewpoint.

For laboratory tests, ECG status, and physical examination results, positive and abnormal changes in clinically significant determinations for each indicator at baseline and after treatment will be summarized in a cross-tabulation. The results of the single most severe clinical significance determination after treatment will be selected for summarization, including data from unscheduled visits.

In addition, for laboratory test indicators, severity will be graded according to CTCAE 5.0 and the change in grading between baseline and post-treatment will be summarized in a cross-tabulation. The most severe post-treatment grading will be selected for summarization, including data from unscheduled visits.

All abnormal clinically significant laboratory tests, vital signs, electrocardiograms, and physical examination findings will be tabulated.

The above analysis will be based on the actual collection of visit points and corresponding content in the CRF form.

All analysis will be performed using the SAS® version 9.4 or above.

7. Adverse event reporting

7.1 Adverse Event (AE)

7.1.1 Definition of AE

An AE is any adverse medical event that occurs after a subject in a clinical trial has signed an informed consent form, but is not necessarily causally related to the treatment. An AE can be any unfavorable undesired symptom, sign, laboratory test abnormality, or disease, etc., and includes at least the following:

1) A medical condition/disease that existed prior to the initiation of study treatment is recorded as an adverse event only if it worsens after the initiation of study drug;

2) Any newly occurring AE: Any newly occurring adverse medical condition (including symptoms, signs, and newly diagnosed diseases);

3) Abnormal clinically significant laboratory test values or results that are not caused by concomitant disease.

The investigator should keep a detailed record of any AE that occurs in the subject, including: the name of the AE and a description of all associated symptoms, time of occurrence, severity, relevance to the trial medication, duration, measures taken with respect to the study medication, and final results and regression.

7.1.2. Criteria for determining the severity of AEs

Refer to NCI-CTC AE v5.0 for grading criteria for drug AEs. In the event of an AE not listed in the NCI-CTC AE v5.0 table refer to the following criteria:

Degree	Clinical description of severity
1	Mild; no clinical symptoms or mild clinical symptoms; abnormal clinical or laboratory tests only; no treatment required.
2	Moderate; requires minor, localized or non-invasive treatments; age-appropriate limitations in Activities of Daily Living (ADLs), which are defined as cooking, shopping, talking on the phone, counting money, etc.
3	A condition that is severe or has medically serious symptoms but is not life-threatening at this time; results in hospitalization or prolonged hospitalization; results in a disability; and limits self-care ADLs. Self-care ADLs are: bathing, dressing, undressing, eating, going to the bathroom, taking medication, etc., and are not bedridden.
4	Life-threatening; requires urgent medical treatment.
5	Lead to death.

7.1.3 Determination of the relationship between AE and the trial drug

Adverse events include all unintended clinical manifestations, as long as these events occur after the signing of the informed consent, regardless of whether they are related to the test drug, or even regardless of whether the drug is applied or not, they should be reported as adverse events, and all adverse events must be presented in the form of clinical reports. Any complaints of discomfort or abnormal changes in objective laboratory tests during the treatment period should be faithfully recorded, along with the severity, duration, treatment measures and regression of the adverse event. The investigator should comprehensively determine the relationship between the AE and the test drug, such as whether the occurrence of the AE has a reasonable time sequence with the drug, the characteristics of the study drug, the toxicological and pharmacological effects of the study drug, whether the subject is using other drugs, the subject's underlying disease, medical history, family history, and de-priming and re-priming reactions, etc. The study should be conducted in accordance with the principle of "definitely related, definitely related, definitely related, definitely related, and

definitely related". Possible associations between adverse events and the test drug were evaluated according to a five-level classification: definitely related, probably related, probably unrelated, definitely unrelated, and undetermined.

7.2 Serious Adverse Event (SAE)

7.2.1 Definition of SAE

SAE is defined as the following adverse medical event at any dose:

• Leads to death;

• Life-threatening. Life-threatening (the term "life-threatening" means that there is an immediate risk of death to the subject at the time of the event/reaction; it does not mean that death may occur only if the event/reaction deteriorates further);

• Hospitalization is required or existing hospitalization is prolonged;

• An event that results in permanent or severe disability/malfunction/impairment of ability to work;

• Congenital anomalies or birth defects;

• Other important medical events.

Medical and scientific judgment must be applied to determine whether other conditions should be considered serious events, such as significant medical events that may not be immediately life-threatening, result in death or hospitalization, but may endanger the patient or require intervention to prevent one of the outcomes listed in the definition above. Examples include allergic bronchospasm treated as an emergency in an emergency room or treated at home, malaise or convulsions not resulting in hospitalization, development of drug dependence or abuse.

7.2.2 Hospitalization

Adverse events in clinical trials that result in hospitalization (even if less than 24 hours) or prolongation of an existing hospital stay should be considered SAEs.

Hospitalizations or extended stays due to the following are not required to be reported as SAEs:

• Rehabilitation organization

• Nursing homes

• Routine emergency room admissions

• Same-day surgery (e.g., outpatient/same-day/ambulatory)

• Social reasons (health insurance reimbursement, etc.)

• Hospitalization or prolonged hospitalization unrelated to worsening AE is not itself an SAE. for example:

- Admission to the hospital for a pre-existing condition with no new adverse event or exacerbation of the pre-existing condition (e.g., in order to check for laboratory test abnormalities that have persisted to date prior to the trial);
- Hospitalization for management reasons (e.g., routine annual physicals);
- Hospitalization as defined by the trial protocol during the clinical trial (e.g., as required by the trial protocol);
- Elective hospitalization not related to an adverse event (e.g., elective surgery);
- Planned treatments or surgical procedures should be documented throughout the trial protocol and/or in the individual subject's baseline information;
- Admission for blood product use only.

Diagnostic or therapeutic invasive (e.g., surgical), noninvasive procedures should not be reported as an AE, but the disease condition that led to the procedure should be reported if it meets the definition of an AE, e.g., acute appendicitis that develops during the AE reporting period should be reported as an AE, and the appendectomy that results in it should be documented as the treatment for that AE.

7.2.3 Disease progression and death

Disease progression was defined as deterioration of the subject's condition caused by the indication for the study. This included imaging progression and progression of clinical signs and symptoms. New metastases from the primary tumor, or progression of pre-existing metastases were considered disease progression. Events that are life-threatening, require hospitalization or prolonged hospitalization, or result in permanent or severe disability/malfunction/impairment of ability to work, congenital anomalies, or birth defects due to signs and symptoms of disease progression are not reported as SAE. If there is any uncertainty as to whether the SAE is due to disease progression, it should be reported as an SAE.

In the study population of this trial, "disease progression" is an expected occurrence and should not be reported as an AE term. When disease progression occurs, the event used to confirm disease progression should be reported as an AE. e.g., a subject develops epilepsy that is determined to be related to brain metastases, and the AE terminology should be documented as "epilepsy" rather than "disease progression" or "brain metastases".

If a subject dies during the trial, it must be reported as an SAE, regardless of whether or not new antitumor therapy has been received (refer to AE/SAE Collection and Follow-Up Duration Principles). Deaths assessed by the Investigator to be due to signs and symptoms of disease progression should be recorded in the eCRF and reported as an SAE. The term "death" should not be used as an AE or SAE term, but rather as the result of an event, and the event that caused or contributed to the death should be recorded as an AE or SAE. If the cause of death is unknown and cannot be determined at the time of reporting, the AE or SAE terminology should be recorded as "unexplained death". ".

7.2.4 Potential drug-related liver damage

Abnormal AST and/or ALT levels combined with abnormally elevated total bilirubin levels will be considered drug induced liver injury if the following conditions (1) (2) (3) are met and there is no other cause of liver injury. Such conditions should always be considered as significant medical events.

**Conditions**	**Standard of judgment**
(1) Abnormal ALT or AST	Baseline normal: ALT or AST ≥ 3 × ULN during treatment;<br>Abnormal baseline: treatment-period ALT or AST ≥ 2 × baseline level and value ≥ 3 × ULN; or value ≥ 8 × ULN.
(2) TBIL abnormalities	Baseline normal: TBIL > 2 x ULN during treatment;<br>Baseline abnormality: increase in TBIL ≥ 1×ULN or its value > 3×ULN during the treatment period.
(3) No hemolysis and alkaline phosphatase < 2 x ULN (or no information obtained).	

If a subject has an abnormally elevated total bilirubin level in conjunction with an abnormally elevated AST and ALT level during treatment or follow-up, the subject should return to the Study Center for evaluation as soon as possible, preferably within 48 hours, after learning of the abnormal result. The evaluation should include laboratory tests, a thorough medical history and physical assessment, and should consider the possibility of a liver tumor (primary or secondary).

In addition to repeat AST and ALT tests, laboratory tests should include albumin, creatine kinase, total bilirubin, direct and indirect bilirubin, gamma-glutamyltransferase, prothrombin time (PT)/international normalized ratio (INR), and alkaline phosphatase.), alkaline phosphatase, etc. A detailed history should be taken that includes a history of alcohol consumption, acetaminophen, soft drugs, supplements of all types, family history, occupational exposures, sexual behavior, travel, contact with jaundiced patients,

surgery, blood transfusions, liver disease, or allergic disease. Further testing may also include testing for acute hepatitis A, B, C, and E and liver imaging (e.g., biliary tract). If repeat testing still confirms compliance with the above laboratory criteria as defined, the possibility of potential drug-related liver injury should be considered in the absence of other causes of abnormal liver function tests, without waiting to make all liver function etiologic test results. Such cases of potential drug-related liver damage should be reported as SAE.

7.2.5 Perform other anti-tumor treatments

If a subject begins other antineoplastic therapy before the end of the safety reporting period, the reporting period for SAEs that are not deaths, unless suspected to be related to the study drug, ends at the start of the new antineoplastic therapy. If the death occurs within the safety reporting period, it must be reported as an SAE regardless of whether the subject is receiving other therapy.

7.3 AE/SAE reporting

7.3.1 AE/SAE collection and follow-Up

AE/SAE information was collected from the time the subject signed the informed consent form until the end of the safety reporting period.

The investigator should ask at each visit about AE/SAEs that have occurred since the last visit and provide timely follow-up information based on challenge requests received. Refer to the table below for details.

Principles of AE/SAE collection and follow-up periods

Category	Collection requirements	Follow-up requirements
No drug-related AE/SAE	Up to the end of the safety reporting period or initiation of new antitumor therapy (whichever is reached first).	Until the end of the security reporting period.
AE with drug-related	Until the end of the security reporting period.	Follow-up to resolution, response, or to baseline level, or ≤ grade 1, or to steady state, or with reasonable explanation (e.g., lost to visit, death).
SAEs with drug-related	Unlimited duration.	Follow-up to resolution, response, or to baseline level, or ≤ grade 1, or to steady state, or with reasonable explanation (e.g., lost to visit, death).

Note: 1. The safety reporting period is defined as the period from the signing of the informed consent form up to 90 days after the last dose of Camrelizumab or 30 days after the last dose of Apatinib, whichever is longer.

2. If the death occurs within the safety reporting period, it must be reported as SAE.

7.3.2 SAE reporting procedures

In the event of an SAE, whether reported for the first time or as a follow-up report, the investigator must immediately fill in the Serious Adverse Event Reporting Form, sign and date it, and report it immediately to the principal investigator and Hengrui drug safety department within 24 hours of the investigator being notified of it, and report it to the relevant supervisory and regulatory authorities and ethics committees in a timely manner in accordance with the requirements of the regulation. SAEs occurring after the safety reporting period should be collected from those suspected to be related to the study drug.

All SAEs should be documented in detail with respect to symptoms, severity (CTCAE classification), association with all trial medications, time of onset, time of management, measures taken with respect to all study medications individually, time and mode of follow-up, and regression. If an SAE is not considered by the investigator to be related to the trial drug, but is potentially related to a study condition (e.g., termination of the original treatment, or comorbidities during the course of the trial), this relationship should be detailed in the narrative portion of the SAE report form. If the intensity of an ongoing SAE or its relationship to the test drug changes, a follow-up report should be submitted immediately. If the investigator believes that information was misreported in a previously reported SAE, a correction, withdrawal, or downgrading statement may be made in the follow-up report and reported in accordance with the SAE reporting procedures.

The email address for receiving SAE reports for this project for the Hengrui Drug Safety Department is hengrui_drug_safety@hengrui.com.

7.4 Pregnancy Reporting

If a female subject becomes pregnant during the clinical study, the subject will be discharged from the group, and if the partner of a male subject becomes pregnant during the clinical study, the subject will continue the clinical study. The investigator should report the pregnancy to the sponsor and the Hengrui Drug Safety Department by

completing the Hengrui Clinical Study Pregnancy Report/Follow-Up Form within 24 hours of notification and report the ethics in a timely manner.

The investigator will follow the pregnancy to final outcome and report the results to the sponsor and the Hengrui Drug Safety Department, and must follow the pregnancy until 1 month after the mother has given birth.

If the pregnancy results in stillbirth, spontaneous abortion, or fetal malformation, it is considered an SAE and needs to be reported according to the SAE timeline requirements.

If a subject has a concurrent SAE during pregnancy, a Serious Adverse Event Report Form will also be completed and the SAE reporting procedures must be followed for reporting.

Reference

1. Bray F, Ferlay J, Soerjomataram I, et al. Global cancer statistics 2018: GLOBOCAN estimates of incidence and mortality worldwide for 36 cancers in 185 countries. CA Cancer J Clin. 2018.

2. Chen WQ, Zheng RS, Zhang SW, et al. Report of incidence and mortality in china cancer registries, 2008. Chin J Cancer Res. 2012 Sep;24(3):171-80.

3. Duan JJ, Yan YQ, Yang NN, et al. International comparison analysis of China's cancer incidence and mortality. Chinese Journal of the Frontiers of Medical Science (Electronic Version). 2016;8(07):17-23.

4. Chen Wanqing, Zheng Rongshou, Baade Peter D, et al. Cancer statistics in China, 2015. CA: a cancer journal for clinicians,2016,66(2).

5. Herbst RS, Heymach JV, Lippman SM. Lung cancer. New England Journal of Medicine, 2008, 359(13): 1367-1380.

6. www.seer.cancer.gov. Accessed 08/08/2012.

7. Goldstraw P, Chansky K, Crowley J, et al. The IASLC Lung Cancer Staging Project: Proposals for Revision of the TNM Stage Groupings in the Forthcoming (Eighth) Edition of the TNM Classification for Lung Cancer. J Thorac Oncol. 2016;11(1):39-51.

8. Paz-Ares L, Horn L, Borghaei H, et al. Phase III, Randomized Trial (CheckMate 057) of Nivolumab versus Docetaxel in Advanced Non-squamous (non-SQ) Cell Non-small Cell Lung Cancer (NSCLC). Presented at ASCO Annual Meeting 2015, May 29-June 2, 2015.

9. NSCLC Meta-analyses Collaborative Group. Preoperative chemotherapy for non-small-cell lung cancer: a systematic review and meta-analysis of individual participant data. The Lancet,2014,383(9928).

10. Dunn GP, Bruce AT, Ikeda H et al. Cancer immunoediting: from surveillance to tumor escape Nat Immunol. 2002 Nov;3(11):991-8.

11. Nishimura H, Honjo T, PD-1: an inhibitory immunoreceptor involved in peripheral tolerance. Trends Immunol. 2001 May; 22(5):265-8.

12. Carter L, Fouser LA, Jussif J et al. PD-1: PD-L inhibitory pathway affects both CD4 (+) and CD8 (+) T cells and is overcome by IL-2. Eur J Immunol. 2002 Mar;32(3):634-43.

13. Langer Corey J, Gadgeel Shirish M, Borghaei Hossein et al. Carboplatin and pemetrexed with or without pembrolizumab for advanced, non-squamous non-small-cell lung cancer: a randomised, phase 2 cohort of the open-label KEYNOTE-021 study. The Lancet. Oncology,2016,17(11).

14. Rittmeyer Achim, Barlesi Fabrice, Waterkamp Daniel et al. Atezolizumab versus docetaxel in patients with previously treated non-small-cell lung cancer (OAK): a phase 3, open-label, multicentre randomised controlled trial. Lancet,2017,389(10066).

15. Konishi J, Yamazaki K, Azuma M et al B7-H1 expression on non-small cell lung cancer cells and its relationship with tumor-infiltrating lymphocytes and their PD-1 expression Clin Cancer Res. 2004 Aug 1;10(15):5094-100.

16. Dong H, Chen L B7-H1 pathway and its role in the evasion of tumor immunity J Mol Med (Berl). 2003 May;81(5):281-7.

17. Joseph RW, Sullivan RJ, Harrell R et al Correlation of NRAS mutations with clinical response to high-dose IL-2 in patients with advanced melanoma J Immunother. 2012 Jan; 35(1):66-72.
18. Azuma T, Yao S, Zhu G et al B7-H1 is a ubiquitous antiapoptotic receptor on cancer cells Blood. 2008 Apr 1;111(7):3635-43.
19. Dong H, Strome SE, Salomao DR et al Tumor-associated B7-H1 promotes T-cell apoptosis: A potential mechanism of immune evasion. Nat Med. 2002 Aug;8(8):793-800.
20. Iwai Y, Terawaki S, Honjo T PD-1 blockade inhibits hematogenous spread of poorly immunogenic tumor cells by enhanced recruitment of effector T cells. Int Immunol. 2005 Feb;17(2):133-44.
21. Hirano F, Kaneko K, Tamura H et al Blockade of B7-H1 and PD-1 by monoclonal antibodies potentiates cancer therapeutic immunity. Cancer Res. 2005 Feb 1;65(3):1089- 96.
22. Forde PM, Chaft JE, Smith KN, et al., Neoadjuvant PD-1 Blockade in Resectable Lung Cancer. N Engl J Med. 2018 May 24;378(21):1976-1986.
23. Rusch V W, Chaft J E, Johnson B, et al. Neoadjuvant atezolizumab in resectable non-small cell lung cancer (NSCLC): Initial results from a multicenter study (LCMC3) [J]. Journal of Clinical Oncology, 2018, 36, 8541.
24. Li N,Ying JM,Tao XL,et al.Efficacy and Safety of Neoadjuvant PD-1 Blockade with Sintilimab in Resectable Non-Small Cell Lung Cancer[EB/OL].WCLC 2019, abstract JCSE01.10.
25. Cascone T, William W N, Weissferdt A, et al. LBA49 Neoadjuvant nivolumab (N) or nivolumab plus ipilimumab (NI) for resectable non-small cell lung cancer (NSCLC)[J]. Annals of Oncology, 2018, 29(suppl_8): mdy424. 059.
26. Cascone T, William WN, Weissferdt A,et al.Neoadjuvant nivolumab (N) or nivolumab plus ipilimumab (NI) for resectable non-small cell lung cancer (NSCLC): Clinical and correlative results from the NEOSTAR study[J].J Clin Oncol ,2019,37 (suppl; abstr 8504).
27. Provencio-Pulla M, Nadal-Alforja E, Cobo M, et al. Neoadjuvant chemo/immunotherapy for the treatment of stages IIIA resectable non-small cell lung cancer (NSCLC): A phase II multicenter exploratory study—NADIM study-SLCG[J]. Journal of Clinical Oncology, 2018, 36, 8521.
28. Shu CA, Gainor JF, Awad MM, et al. Neoadjuvant atezolizumab and chemotherapy in patients with resectable non-small-cell lung cancer: an open-label, multicentre, single-arm, phase 2 trial. Lancet Oncol. 2020;21(6):786-795. doi:10.1016/S1470-2045(20)30140-6.
29. J Heymach, L Krilov, A Alberg, et al. Clinical cancer advances 2018: annual report on progress against cancer from the American Society of Clinical Oncology. J Clin Oncol, 36 (2018), pp. 1020-1044.
30. Wu et. A phase II umbrella study of camrelizumab in different PD-L1 expression cohorts in pre-treated advanced/metastatic NSCLC. 2019 WCLC JCSE01.09.

31. Zhou et. A randomized phase 3 study of camrelizumab plus chemotherapy as 1st line therapy for advanced/metastatic non-squamous non-small cell lung cancer. 2019 WCLC A04.03.
32. Wong ML, Prawira A, Kaye AH, et al. Tumour angiogenesis: its mechanism and therapeutic implications in malignant gliomas. Journal of Clinical Neuroscience Official Journal of the Neurosurgical Society of Australasia, 2009, 16(9): 1119-1130.
33． Lin B, Song X, Yang D, et al. Anlotinib inhibits angiogenesis via suppressing the activation of VEGFR2, PDGFRβ and FGFR1. Gene, 2018.
34． Hall RD, Le TM, Haggstrom DE, et al. Angiogenesis inhibition as a therapeutic strategy in non-small cell lung cancer (NSCLC). Transl Lung Cancer Res, 2015, 4(5): 515-523.
35. Tian Shu, Quan Haitian, Xie Chengying, et al. YN968D1 is a novel and selective inhibitor of vascular endothelial growth factor receptor-2 tyrosine kinase with potent activity in vitro and in vivo. Cancer Science,2011,102(7).
36. Zhang L, Shi M, Huang C, et al. A phase II multicenter placebocontrolled trial of apatinib in patients with advanced nonsquamous non-small cell lung cancer(NSCLC) after two previous treatment regiments． J Clin Oncol,2012,30: S7548.
37. Chen EH, Chen ZC, Chen ML, et al. Clinical evaluation of Apatinib mesylate tablets in the treatment of advanced non-small cell lung cancer. China Modern Medicine, 2017, 24 (13) :91-93
38. Guo YJ, Jing XH. Efficacy and safety analysis of docetaxel combined with Apatinib in second-line treatment of non-squamous carcinoma non-small cell lung cancer. Chinese Journal of Clinical Oncology, 2017, 44(11) :544-546.
39. Haruhiro Saito, Tatsuro Fukuhara, Naoki Furuya, et al. Erlotinib plus bevacizumab versus erlotinib alone in patients with EGFR -positive advanced non-squamous non-small-cell lung cancer (NEJ026): interim analysis of an open-label, randomised, multicentre, phase 3 trial. The Lancet Oncology, 2019,20(5).
40. Zhou et. al.Phase Ib study of apatinib combined with SHR-1210 (PD-1) in patients with advanced NSCLC. 2018 ASCO e21017.41. Zhou et. Efficacy of PD-1 monoclonal antibody SHR-1210 plus apatinib in patients with advanced non-squamous NSCLC with wild-type EGFR and ALK. Journal of Clinical Oncology 37, no. 15_suppl (May 20, 2019) 9112-9112.
